# Dual‐Peptide Nanoplatform: Mesoporous Silica Nanoparticles Functionalized With a Cell‐Penetrating Peptide and Loaded With Rationally Designed Antimicrobial Peptides for Tuberculosis Therapy

**DOI:** 10.1002/adhm.202504285

**Published:** 2026-03-29

**Authors:** Christian S. Carnero Canales, Cesar Augusto Roque‐Borda, Ana Carolina Cerqueira Negri, Oswaldo Ramirez Delgado, Letícia Oliveira Catarin Nunes, Flávia Aparecida Resende, Hernane Barud, Norival Alves Santos‐Filho, Saulo Santesso Garrido, Karen Cristina Oliveira, Alexandra Ivo de Medeiros, Rafael Miguel Sábio, Fernando Rogério Pavan

**Affiliations:** ^1^ Tuberculosis Research Laboratory School of Pharmaceutical Sciences São Paulo State University (UNESP) Araraquara Brazil; ^2^ Institute of Chemistry São Paulo State University (UNESP) Araraquara Brazil; ^3^ Department of Biological and Health Sciences University of Araraquara (UNIARA) Araraquara São Paulo Brazil; ^4^ Department of Biological Sciences School of Pharmaceutical Sciences São Paulo State University (UNESP) Araraquara Brazil

**Keywords:** antimicrobial peptides, cell‐penetrating peptide, galleria mellonella, in silico, mesoporous silica nanoparticles, mycobacterium tuberculosis

## Abstract

Tuberculosis remains a major infectious disease worldwide and demands new therapeutic approaches capable of targeting intracellular *Mycobacterium tuberculosis*. Using a machine learning (ML)–guided design strategy based on the plectasin scaffold, a peptide library was generated and DC05 was identified as the most active analog. As a free peptide, DC05 inhibited the H37Rv strain with an MIC_90_ of 12.46 ± 0.06 µm, displayed low cytotoxicity in MRC‐5 fibroblasts (IC_50_ >250 µm) and J774A.1 macrophages (115.18 ± 8.49 µm), and increased ethidium bromide accumulation to levels comparable to verapamil, with minimal hemolysis up to 256 µm. DC05 was then encapsulated into mesoporous silica nanoparticles (MSN), yielding (MSN@DC05) and further functionalized with tuftsin, a cell‐penetrating peptide (CPP) (MSN‐CPP@DC05). In simulated pulmonary fluid at 0.5 mg mL^−1^, all formulations remained colloidally stable, with hydrodynamic diameters between 122 and 164 nm and low polydispersity. Adsorption onto MSN followed a Langmuir‐type profile (qmax 39.5 ± 4.9 mg g^−1^). Release was strongly dependent on the surrounding medium, showing rapid diffusion in simulated pulmonary fluid, slower release in simulated intestinal fluid, and almost no detectable peptide in simulated gastric fluid, where acid‐driven degradation is likely. Nanoencapsulation enhanced antimycobacterial potency, reducing the MIC_90_ to 5.74 ± 0.15 µm for MSN@DC05 and 4.13 ± 0.64 µm for MSN‐CPP@DC05, while maintaining high viability in mammalian cells up to 500 µg mL^−1^ in macrophages and 2000 µg mL^−1^ in fibroblasts. Ames testing showed mutagenicity indices below 2, and *Galleria mellonella* assays at 2000 mg kg^−1^ confirmed high survival. Scanning electron microscopy combined with molecular dynamics supported a membrane‐disruptive mechanism for MSN‐CPP@DC05. These results indicate that integrating ML‐guided peptide design with a functionalized mesoporous carrier improves intracellular delivery and antimycobacterial activity while maintaining favorable safety profiles.

## Graphical abstract

Machine learning–guided engineering of a plectasin‐derived peptide yields DC05, a potent antimycobacterial candidate. Encapsulation into tuftsin‐functionalized mesoporous silica nanoparticles enhances intracellular delivery, stability, and activity against *Mycobacterium tuberculosis* while maintaining low cytotoxicity and minimal hemolysis. The combined design–nanocarrier strategy advances peptide‐based therapeutics and offers a promising platform for treating drug‐resistant tuberculosis.

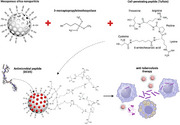

## 1 Introduction

Tuberculosis (TB) remains a major global health problem, aggravated by the growing prevalence of multidrug‐resistant (MDR) and extensively drug‐resistant (XDR) strains of *Mycobacterium tuberculosis* (*Mtb*) [1, 2]. Current treatment regimens depend on prolonged combinations of antibiotics, which are frequently associated with adverse effects, poor adherence, and further selection of resistant mutants [3, 4]. These limitations have driven the search for alternative therapeutic strategies that directly target *Mtb* and circumvent conventional resistance mechanisms, including new drug delivery platforms and antimicrobial peptides (AMPs) with improved specificity and potency [4, 5].

AMPs are attractive candidates because they display broad antimicrobial activity and often act through membrane‐disrupting mechanisms that differ from those of standard antibiotics [5, 6, 7]. However, their application is constrained by susceptibility to proteolysis, limited stability in physiological fluids, and the difficulty of achieving efficient intracellular delivery to pathogens such as *Mtb* [8, 9]. Overcoming these barriers requires carriers able to protect the peptide cargo, preserve its activity in biological media, and promote accumulation at the intracellular niches where *Mtb* resides.

Among available delivery platforms that address some of these requirements, mesoporous silica nanoparticles (MSNs) offer protection of peptide cargo and tunable release under physiological conditions [10, 11]. Their silanol‐rich surface enables the attachment of ligands that facilitate cellular uptake, while thiol functionalization allows disulfide‐based peptide conjugation that is responsive to intracellular redox environments [12, 13]. Incorporating cell‐penetrating motifs such as tuftsin further promotes macrophage targeting, which is relevant because macrophages constitute the main intracellular reservoir of *Mtb* [14].

In parallel with advances in nanocarrier design, artificial intelligence (AI) and, in particular, machine learning (ML) have transformed peptide discovery workflows by enabling high‐throughput screening, sequence optimization, and in silico prediction of bioactivity profiles [15, 16]. ML‐based tools can be trained to predict antimicrobial activity, cytotoxicity, hemolysis, and antibiofilm properties, among other features, which allows the construction of focused peptide libraries prior to experimental validation [17].

In this study, we combined ML‐guided peptide optimization with an MSN‐based nanoplatform tailored for macrophage delivery. ML tools were used to generate a library of plectasin‐derived AMP variants optimized for anti‐*Mtb* activity and biocompatibility, and the most active sequence was conjugated to tuftsin‐functionalized MSNs via disulfide chemistry. The goal was to obtain AMP analogs with improved activity and selectivity against *Mtb* and to develop a CPP‐conjugated MSN system designed to improve peptide stability, promote intracellular uptake by macrophages, and modulate the apparent release behavior according to the surrounding medium.

## 2 Materials and Methods

### 2.1 Chemical Reagents

Middlebrook 7H9 broth supplemented with oleic acid, albumin, dextrose and catalase (OADC) was purchased from Difco Laboratories (Detroit, MI, USA). Dulbecco's modified Eagle's medium (DMEM) and Roswell Park Memorial Institute medium (RPMI‐1640) were obtained from Vitrocell (São Paulo, Brazil). Fetal bovine serum (FBS), gentamicin sulfate (BioReagent), amphotericin B (BioReagent), resazurin sodium salt (certified by the Biological Stain Commission), ethidium bromide (EtBr, 95%), verapamil hydrochloride (Pharmaceutical Secondary Standard), dimethyl sulfoxide (DMSO, 99.9%), fluorescamine (98%), hydroxybenzotriazole (HOBt, 98%), diisopropylcarbodiimide (DIC, 99%), trifluoroacetic acid (TFA, 99%), triisopropylsilane (TIS, 98%), 1,2‐ethanedithiol (EDT 98%), tetraethyl orthosilicate (TEOS, 99%), cetyltrimethylammonium bromide (CTAB, 98%), sodium hydroxide (97%), ethanol (99%), ammonium nitrate (98%), 3‐aminopropyltriethoxysilane (APTES, 99%), fluorescein isothiocyanate (FITC, 90%), 3‐mercaptopropyltrimethoxysilane (MPTMS, 95%), 4‐(2‐hydroxyethyl)‐1‐piperazineethanesulfonic acid (HEPES, 99.5%), hydrogen peroxide (30%) sodium chloride (99%), and sodium iodide (99.5%) were obtained from Sigma–Aldrich (St. Louis, MO, USA). Magnesium chloride (MgCl_2_, 98%), sodium chloride (NaCl, 99%), potassium chloride (KCl, 99%), sodium phosphate dibasic (Na_2_HPO_4_, 99%), sodium bicarbonate (NaHCO_3_, 99.5%), sodium sulfate (Na_2_SO_4_, 99%), calcium chloride (CaCl_2_, 99%), sodium acetate (99%), sodium citrate (99%), hydrochloric acid (HCl, 37%), ethanol (99.5%), and Tween 80 (99%) were supplied by Merck KGaA (Darmstadt, Germany). Dimethylformamide (DMF, 99.8%) was purchased from Neon Comercial (São Paulo, Brazil), and dichloromethane (DCM, 99.8%) was purchased from Anidrol Products Laboratories (São Paulo, Brazil). Nutrient broth No. 2 was purchased from Oxoid Ltd. (Basingstoke, UK). The lyophilized S9 fraction, derived from the liver microsomes of Sprague–Dawley rats induced with Aroclor 1254, was purchased from Moltox Molecular Toxicology, Inc. (Boone, NC, USA). All buffer solutions, including phosphate‐buffered saline (PBS) and Tris‐buffer (0.01 m Tris‐HCl, 0.15 m NaCl, pH 7.4), were freshly prepared in‐house via analytical‐grade reagents and ultrapure water. All amino acids used for peptide synthesis were purchased from AAPPTEC (Louisville, KY, USA).

### 2.2 ML‐Guided Peptide Discovery and Optimization

#### 2.2.1 ML‐Based Design of an AMP Library

A search was conducted in the AntiTBPdb database to identify peptides with anti‐*Mtb* activity, with a focus on their cytotoxic and therapeutic potential [18]. Plectasin was chosen as the reference sequence because of the known activity of its analogs against *Mtb* [19]. To increase the structural accuracy, the reference sequence was aligned with its analogs via the Clustal tool in Jalview software, enabling the identification of conserved and variable regions [20]. Nonrelevant fragments were discarded, and regions with the highest potential for activity were retained for further study.

#### 2.2.2 Bioactivity Prediction and AMP Optimization

To pinpoint the region of plectasin that interacted most effectively with *Mtb*, the full sequence was separated into two portions that preserved its main structural elements, one dominated by the α‐helical segment and the other by the β‐sheet region. Each portion was examined with AntiTBpred to estimate its activity against *Mtb*. The segment that showed the strongest response served as the basis for producing a small set of variants generated through targeted amino‐acid substitutions, mainly replacing cysteine or glycine to reduce instability associated with those residues. The modified sequences were then reassessed with AntiTBpred and PeptideRanker, using previously characterized peptide features to anticipate which variants were more likely to retain or improve activity against *Mtb* [21].

#### 2.2.3 Pharmacokinetic and Stability Predictions

The pharmacokinetic properties of the selected peptide variants were predicted via SMILES sequences via the pKCSM web server [22]. The analysis covered:

- Absorption: Water solubility, intestinal absorption, skin permeability, and Caco‐2 permeability.
- Distribution: Central nervous system permeability, blood‒brain barrier (BBB) permeability, Vdss, and plasma protein binding.
- Metabolism: Identification of CYP enzyme substrates and inhibitors.
- Excretion: Total clearance and renal OCT2 substrate prediction.
- Toxicity: AMES toxicity, maximum tolerated dose, hERG channel inhibition, and acute oral toxicity.

Additionally, the half‐life of four peptide sequences was predicted via ProtParam, which was used to evaluate the stability across mammalian reticulocytes, *Escherichia coli*, and yeast models [23].

#### 2.2.4 Molecular Docking and Binding Interaction Analysis

To construct the structural model of the DC05 AMP, both I‐TASSER and AlphaFold3 were used, with model selection guided by tool‐specific metrics (C‐scores for I‐TASSER, pLDDT and PAE for AlphaFold3) [24, 25, 26]. Validation via a Ramachandran plot indicated that the AlphaFold3 model was optimal, with approximately 90% of residues in allowed regions [27, 28]. This model was thus chosen for molecular docking, following energy minimization with the Amber force field in Avogadro to ensure structural integrity and biological relevance. Molecular docking of the DC05 peptide was carried out with *Mtb* target proteins involved in essential processes, following the panel proposed by Primo et al. [29], and included Ag85B, GyrB, LDT2, GlfT2, PknB, CmaA2, and EmbC. For the molecular docking stage, peptide‐protein complexes were generated with LightDock, taking advantage of its capacity to sample receptor flexibility through the anisotropic network model rather than relying on a fixed backbone. After the search procedure, the highest‐scoring poses were examined with the Protein‐Ligand Interaction Profiler to map the contacts that remained consistently across the best solutions. Estimates of binding free energy (∆G) and the corresponding dissociation constants (Kd) were obtained with PRODIGY, which enabled a quantitative comparison of complex stability [30]. This set of analyses outlined how DC05 engages the selected *Mtb* proteins and clarified the interaction patterns that support its activity.

#### 2.2.5 Molecular Dynamics Simulations

The interaction of DC05 with a mycolic‐acid membrane was examined through molecular dynamics (MD) simulations [31] The system was prepared by positioning the peptide above a pre‐equilibrated α‐mycolic acid bilayer, solvating it with explicit water and adding counterions for charge neutrality. Energy minimization was carried out for 20,000 steps using the steepest‐descent method, followed by a 10 ns equilibration under NPT conditions at 310 K and 1 bar with the Berendsen thermostat and barostat. A 100 ns production run was then generated under the same ensemble. All MD simulations were performed in GROMACS 2024 with the GROMOS 54A7 force field. Particle Mesh Ewald was applied to treat long‐range electrostatics, and both van der Waals and Coulomb interactions used a 1.2 nm cutoff. RMSD, RMSF and the center‐of‐mass distance were computed to track changes in peptide conformation and its engagement with the membrane. Since DC05 interacts with *Mtb* after being released from the MSN‐CPP carrier, only the free peptide was included in the MD system [44].

### 2.3 Peptide—In Vitro Analyses

#### 2.3.1 Synthesis of Selected AMPs, Purification, and Characterization

Peptides (AMP and CPP) were synthesized by solid‐phase Fmoc chemistry following the procedure described by Merrifield et al. [32]. Rink amide resin was first swollen in a DMF/DCM mixture, after which the Fmoc groups were removed with 20% 4‐methylpiperidine in DMF. Amino acids were introduced sequentially using HOBt/DIC and stirred for 2 h at room temperature, with DMF and DCM washes between couplings. Coupling progress was monitored with the Kaiser test; a clear resin indicated completion, whereas a blue coloration signaled an incomplete step. Once chain assembly was finished, the peptides were released from the resin using a cleavage mixture of TFA, TIS, EDT, and water (94:2.5:1:2.5) and then lyophilized. Purification and analytical characterization were carried out by HPLC on a Shimadzu Prominence system equipped with a C18 reversed‐phase column, using aqueous TFA and acetonitrile/TFA at 1 mL/min for 30 min. Fractions identified as pure were pooled, lyophilized, and kept at −20°C. Peptide identity was verified by LC‐MS (Shimadzu/Bruker) under conditions comparable to those used for HPLC, employing a 5%–95% gradient over 30 min and monitoring at 220 and 280 nm.

#### 2.3.2 Evaluation of Antimycobacterial Activity

The MIC_90_ against *Mtb* was determined using the resazurin microtiter assay (REMA) following the procedure adapted from Palomino et al. [33]. Bacterial cultures were adjusted to a 1 McFarland standard (3 × 10^8^ CFU/mL), then diluted 1:100 in PBS. Peptide dilutions were arranged in 96‐well plates by combining 100 µL of each peptide solution with an equal volume of Middlebrook 7H9 medium supplemented with OADC and sodium chloride. Afterward, 100 µL of the diluted bacterial suspension was dispensed into each well, resulting in a total volume of 200 µL. Plates were incubated at 37°C for seven days. At the end of this period, 30 µL of a 0.01% resazurin solution was added, and the plates were returned to the incubator for another 24 h. Fluorescence was recorded with a Synergy H1 microplate reader (BioTek, Winooski, VT, USA) using excitation and emission wavelengths of 530 and 590 nm. All assays were conducted independently in triplicate.

#### 2.3.3 Cytotoxicity Evaluation

In vitro cytotoxicity assays followed the procedure described by Silva et al. [34], with minor adjustments. MRC‐5 cells were maintained in DMEM, whereas J774A.1 macrophages were grown in RPMI‐1640 supplemented with 10% fetal bovine serum, gentamicin sulfate (50 mg/L), and amphotericin B (2 mg/L). Cultures were kept at 37°C in a 5% CO_2_ atmosphere until reaching confluence. Cells were then adjusted to 2.5 × 10^5^ cells/mL, seeded at 100 µL per well in 96‐well plates, and allowed to attach for 24 h. After this period, the cultures were exposed to peptide concentrations ranging from 16 to 256 µm for an additional 24 h. Viability was assessed by adding 30 µL of a 0.01% resazurin solution to each well and incubating for 3 h. Fluorescence was recorded on a Synergy H1 reader (BioTek) at 530/590 nm. All assays were carried out in triplicate, and viability values were expressed relative to untreated controls.

#### 2.3.4 Efflux Pump Inhibition Assay

The efflux pump activity was assessed following the methodology described by Caleffi‐Ferracioli et al. [35]. *Mtb* H37Rv was grown for 15–21 days in Middlebrook 7H9 broth supplemented with OADC. Cultures were diluted to an optical density (OD_600_) between 0.6 and 0.8, washed, and resuspended in PBS with 0.05% Tween 80 to a final OD_600_ of 0.44. Bacterial suspensions (100 µL) were incubated with peptides at 1/2 MIC_90_ in the presence of EtBr. Fluorescence readings were taken at three‐min intervals for 2 h via black microplates. Relative fluorescence fractions (RFFs) were calculated compared with those of the control samples, and the assays were performed in triplicate.

#### 2.3.5 Hemolytic Activity Evaluation

Hemolytic activity was assessed following the procedure of Serrano et al. [36]. Human erythrocytes were rinsed three times with Tris‐buffered saline (0.01 m Tris‐HCl, 0.15 m NaCl, pH 7.4) and adjusted to a 1% suspension. Peptides were first prepared at 1024 µm, then serially diluted and mixed with erythrocytes in equal volumes (100 µL peptide solution and 100 µL erythrocytes). Samples were incubated at 37°C for 1 h and centrifuged at 3000 x g for 2 min. A 100‐µL aliquot of each supernatant was transferred to 96‐well plates, and absorbance at 540 nm was recorded with a Bio‐Rad model 3550 reader. Triton X‐100 (1%) was used as the positive control, yielding complete hemolysis. HC_50_ values were obtained by logarithmic regression. All measurements were carried out in triplicate, and results are reported as mean ± standard deviation.

#### 2.3.6 Reverse Gene Mutation Assay

The mutagenic potential of the peptides was examined using the reverse gene mutation assay (Ames test), following the procedure first described by Ames et al. [37]. *Salmonella typhimurium* strains TA98, TA97a, TA100 and TA102 were revived from frozen stocks by inoculating each strain into 30 mL of Oxoid No. 2 nutrient broth and incubating the cultures at 37°C with shaking at 160 rpm for 12–16 h, yielding cell densities in the range of 1–2 × 10^9^ cells/m. For assay preparations, 100 µL of overnight bacterial cultures were preincubated for 30 min at 37°C with different concentrations of the peptides, which were previously selected on the basis of preliminary toxicity screening (ranging typically from 16 to 256 µm). This preincubation step included DC05 solutions, bacterial suspensions, and either the presence or absence of an exogenous metabolic activation system (S9 mix). The S9 microsomal fraction was freshly prepared from rat liver homogenates induced with Aroclor‐1254, and its metabolic activation mixture contained a 4% fraction, 0.4 m MgCl_2_, 1.65 m KCl, 1 m glucose‐6‐phosphate, and 0.1 m β‐nicotinamide adenine dinucleotide phosphate (NADP). After incubation, the mixtures were combined with 2 mL of top agar and spread evenly onto minimal glucose agar plates, which were incubated at 37°C for 48 h, and after incubation, revertant colonies (His^+^ revertants) were counted manually. Standard mutagens, including 4‐nitro‐o‐phenylenediamine, 2‐aminofluorene, and 2‐aminoanthracene, were used as positive controls for strains with or without the S9 fraction, while DMSO served as the negative control. All the assays were performed independently in triplicate, and the statistical significance of the revertant frequencies was evaluated via Salanal statistical software.

### 2.4 Nanostructure Formulation

#### 2.4.1 Synthesis of Mesoporous Silica Nanoparticles

The synthesis was performed according to the methodology of Sábio et al. [38, 39]. Briefly, 0.606 g of CTAB was dissolved in 180 mL of deionized water under stirring at 60°C in a nitrogen atmosphere. Then, 55.8 mL of octane and 105 µL of styrene (previously purified with NaOH) were added. Subsequently, 0.1381 g of L‐lysine, 6.42 mL of TEOS, and 0.2017 g of AIBA were incorporated. The mixture was stirred for 3 h at 60°C under an inert atmosphere. After the reaction, the suspension was subjected to phase separation, and the light blue phase was centrifuged at 15 000 ×g for 15 min. The MSNs were washed five times with ethanol and water, and the organic agents were removed by treatment with methanol, HCl, and chloroform under reflux at 58°C for 6 h. Finally, the MSNs were centrifuged, washed again with ethanol and water, and dried in an oven at 60°C for 12 h.

#### 2.4.2 Synthesis of MSN‐FITC

FITC was modified following an adapted method from Wang et al., [40]. Briefly, 6.1 µL of APTES was added to 1 mL of anhydrous ethanol under a nitrogen atmosphere at room temperature with magnetic stirring. Then, 11.11 mg of FITC was introduced under light protection and stirred for 12 h. The resulting FITC‐APTES was incorporated through simultaneous condensation of TEOS, following the method by Cheng et al., [41]. The FITC‐labeled nanoparticles (MSN‐FITC and MSN‐FITC‐CPP) were prepared for confocal laser scanning microscopy (CLSM) assays [40, 41].

#### 2.4.3 Synthesis of MPTMS‐Conjugated MSNs

The conjugation of MSNs with 3‐mercaptopropyltrimethoxysilane (MPTMS) was performed following a modified methodology described by Mauline et al. [42]; 1 mL of MPTMS was added to 100 mL of anhydrous acetonitrile, into which 500 mg of MSNs were incorporated. The reaction was carried out under reflux and a protective nitrogen atmosphere at 80°C for 24 h. Subsequently, the obtained nanostructures were centrifuged and subjected to multiple washes, at least three times with ethanol and twice with distilled water, before being dried in an oven at 65°C for 12 h.

#### 2.4.4 Fabrication of CPP‐Conjugated MSN‐MPTMS (MSN‐MPTMS‐CPP)

The CPP‐conjugated MSN‐MPTMS was fabricated via thiol coupling to generate disulfide bonds between the two components. This strategy was based on the use of hydrogen peroxide (H_2_O_2_) as an oxidizing agent and sodium iodide (NaI) as a catalyst, adapted from the methodology of Wang et al. [40]. Initially, a HEPES buffer (20 mm in H_2_O, pH 7.0, containing 3 mm NaCl) and a NaI solution (0.03 mg/mL) were prepared. Ten milligrams of MSN‐MPTMS was added to 1 mL of HEPES buffer, followed by sonication for 5 min. To this solution, 1000 µL of CPP (Cys‐Ahx‐Tuftsin) (1 mg/mL), 50 µL of NaI, and 110 µL of 0.3% H_2_O_2_ were added to 5 mL Eppendorf tubes. The mixture was kept under orbital stirring for 2 h. After this period, the samples were centrifuged at 5000 x g for 10 min and washed at least six times with HEPES buffer (20 mm in H_2_O, pH 7.0).

#### 2.4.5 Determination of CPP Conjugation Efficiency

To quantify CPP conjugation, 1 mg of MSN‐MPTMS‐CPP was suspended in 1 mL of HEPES buffer (20 mm, pH 7.0). Aliquots of 90 µL were transferred to a 96‐well plate and combined with 10 µL of fluorescamine solution (5 mg/mL in acetone). Each condition was prepared in triplicate. The plate was incubated for 5 min at 37°C to allow the reaction between fluorescamine and accessible NH_2_ groups on the CPP‐modified surface. Fluorescence was then recorded at 465 nm with an excitation wavelength of 360 nm [43].

#### 2.4.6 Determination of the Encapsulation Efficiency and Loading Capacity

The encapsulation efficiency (EE%, Equation (1) [44]) and loading capacity (LC%, Equation (2) [45]) of the MSNs were determined via the methodology described previously, with slight modifications. Briefly, 10 mg of the nanoparticles were dispersed in 100 µL of HEPES buffer (10 mm, pH 7.0). The mixture was incubated for 3 h at room temperature with different peptide concentrations (0.14, 0.29, 0.43, 0.714, and 1 mg) to assess the adsorption capacity as a function of the amount of peptide present. After incubation, the nanoparticles were separated via centrifugation and washed to remove excess non‐encapsulated peptide. The amount of non‐adsorbed peptide was determined by analyzing 90 µL of the supernatant mixed with 10 µL of fluorescamine, which indirectly allowed the quantification of the effectively encapsulated peptide through the resulting fluorescence.

(1) $$EE\left(\%\right)=\frac{Total\;peptide-available\;peptide}{Total\;peptide}\times 100$$

(2) $$LC\left(\%\right)=\frac{Peptide\;loaded\;amount}{Total\;weight\;of\;nanparticles}\times 100$$

To characterize the adsorption behavior, the equilibrium concentration of non‐adsorbed peptide (𝐶_𝑒_, mg/mL) was calculated from the supernatant fluorescence values. The amount of peptide adsorbed per mass of MSN (𝑞, mg/g) was determined using:

(3) $$q=\frac{\left(C_{0}-C_{e}\right)V}{m}$$

where 𝐶*_0_* is the initial peptide concentration, 𝑉 the solution volume, and 𝑚 the mass of MSN. Adsorption isotherms were constructed by plotting 𝑞 as a function of 𝐶_𝑒_. The maximum adsorption capacity was obtained by fitting the experimental data to the Langmuir model:

(4) $$q=\frac{q_{max}KC_{e}}{1+KC_{e}}$$

Fitting was performed in OriginPro 2025 using a user‐defined two‐parameter Langmuir function. Only the concentration range consistent with monolayer adsorption was included in the model.

#### 2.4.7 MSNs Characterization

Morphological characterization of the MSNs, MSN‐CPP, and MSN‐CPP@DC05 were performed via high‐resolution transmission electron microscopy (HR‐TEM) on a JEOL TEM‐FEG microscope. For sample preparation, the powders were first dispersed in ethanol via an ultrasonic bath for 15 min to ensure homogeneous dispersion. Then, 3 µL of this suspension was carefully deposited on a copper grid support, allowing the sample to dry at room temperature. This process ensures optimal adhesion of the nanoparticles to the grid and enhances image quality, facilitating a detailed evaluation of the structure and morphology of the MSNs [46]. To assess the mean hydrodynamic diameter and zeta potential of the MSNs, MSN@DC05, MSN‐CPP, and MSN‐CPP@DC05, a Malvern Zetasizer Nano ZS was used. Briefly, 0.5 mg of each nanoplatform was dispersed in 1 mL of HEPES buffer (10 mm, pH 7.0) and sonicated for 30 s, and 700 µL of each dispersion was transferred to measurement cuvettes.

FT‐IR spectra of the nanoplatforms were collected using a Bruker Vertex 70 FT‐IR spectrometer. IR spectra, in absorbance mode, were obtained in the spectral region of 400–4000 cm^−1^ [47]. Thermogravimetric analysis (TGA) of the nanoplatforms were performed to assess the thermal stability and organic content of the bare MSNs and detect each stage of conjugation onto the MSNs. Differential scanning calorimetry (DSC) was conducted in parallel to detect thermal transitions and analyze the heat flow associated with decomposition or phase changes. The samples were placed in alumina crucibles under a constant nitrogen flow (100 mL/min) and heated from 0°C to 600°C at a uniform rate of 5°C/min. Measurements were carried out via an integrated DSC‐TGA system (SDT Q600 V20.9 Build 20, TA Instruments) operated with Universal Analysis 2000 software.

To quantify the free thiol (‐SH) groups in the MSN‐MPTMS, 250 µL of Ellman's reagent (5,5’‐dithiobis‐(2‐nitrobenzoic acid) (0.3 mg/mL dissolved in 0.5 m phosphate buffer, pH 8.0) was added to 250 µL of MSN‐MPTMS (1 mg/mL), and absorbance measurements were taken at 412 nm after a 2‐h incubation period, keeping the samples protected from light throughout the process. The analyses were performed in triplicate to ensure reproducibility. A calibration curve was generated using MPTMS concentrations ranging from 0.125 to 1.25 mm for accurate quantification of thiol groups [48, 49].

#### 2.4.8 In Vitro Release in Simulated Biological Media

Release assays of the DC05 were conducted using simulated biological fluids. Simulated pulmonary fluid (SPF) was prepared by dissolving the following components in 100 mL of ultrapure water: MgCl_2_ (9.85 mg), NaCl (603.2 mg), KCl (40.4 mg), Na_2_HPO_4_ (43.6 mg), NaHCO_3_ (259.6 mg), Na_2_SO_4_ (8.3 mg), CaCl_2_ (35.9 mg), sodium acetate (60.7 mg), and sodium citrate (11.5 mg), adjusting the pH to 7.4 [50]. Simulated gastric fluid (SGF) was prepared as a 0.1 M hydrochloric acid (HCl) solution adjusted to a pH of 1.2, and simulated intestinal fluid (SIF) was prepared using Sorensen's phosphate buffer at pH 6.8 [51].

The methodology was adapted from Tenland et al. [19]. Briefly, MSN‐CPP@DC05 (1 mg) was accurately weighed in triplicate for each biological mixture and dispersed in 1 mL of the corresponding medium. Subsequently, aliquots of 90 µL were transferred to the wells of a 96‐well microplate, to which 10 µL of fluorescamine solution was added. The fluorescence intensity was measured continuously in kinetic mode, initially every min for 30 min, and subsequently every 15 min over an 8‐h period, with excitation/emission wavelengths of 360/465 nm.

#### 2.4.9 Confocal Laser Scanning Microscopy

Cellular internalization of the nanoparticles was evaluated using CLSM. Cells were seeded in 24‐well plates and incubated for 24 h under standard cell culture conditions to allow adhesion. Subsequently, the cells were incubated with MSN‐FITC or MSN‐FITC‐CPP at a final concentration of 25 µg mL^−1^ for 2 h at 37°C in a humidified atmosphere containing 5% CO_2_. After incubation, the cells were washed three times with PBS to remove non‐internalized nanoparticles and analyzed by confocal microscopy using a Carl Zeiss LSM 800 equipped with Airyscan (Carl Zeiss, Germany). Images were obtained by recording only the FITC fluorescence signal associated with the nanoparticles, without the use of additional fluorophores or cellular stains. Fluorescence was detected using the FITC channel with excitation at 488 nm [52].

#### 2.4.10 Flow Cytometry Analysis of Nanoparticle Internalization

To evaluate the internalization of MSN‐FITC and MSN‐FITC‐CPP by RAW 264.7 cells, 2 × 10^5^ cells were seeded in 24‐well plates and incubated for 24 h in 1 mL of DMEM supplemented with 10% fetal bovine serum and 1% antibiotics at 37°C and 5% CO_2_. After this period, the medium was replaced with unsupplemented RPMI‐1640 containing nanoparticles at 25 µg mL^−1^. The experimental negative control consisted of cells cultured in medium alone, without nanoparticles. After 2 h of incubation, the wells were washed with PBS, and the cells were collected using a cell scraper and transferred into FACS tubes. Cell viability was assessed using Fixable Viability Stain 780 (BD Horizo, #565388) at a 1:1000 dilution for 10 min at room temperature. The cells were then fixed with 4% paraformaldehyde (PFA) in PBS for 15 min at 4°C and stored until acquisition. The internalization of MSN‐FITC‐CPP and MSN‐FITC was evaluated by detecting FITC fluorescence (excitation/emission at 488/533 nm) within the viable cell population (RAW 264.7 FVS^−^). Internalization results were expressed as the percentage of FITC^+^ cells and median fluorescence intensity (MFI). Flow cytometry acquisition was performed using a CytoFLEX SRT (Beckman Coulter), and data were analyzed with CytExpert SRT software (Beckman Coulter).

### 2.5 Morphological Evaluation of Mtb

The morphological effects of free DC05 and MSN‐CPP@DC05 on *Mtb* were examined using a JSM‐7001F scanning electron microscope (SEM) (15 kV, Jeol, Tokyo, Japan), following an adapted version of the protocol described by Derengowski et al. [53]. *Mtb* cultures were grown in Middlebrook 7H9 broth supplemented with OADC until they reached the logarithmic growth phase. For each condition (peptide alone, nanoparticles, and untreated control), the cells were harvested via centrifugation at 3000 × g for 10 min, washed twice with sterile phosphate‐buffered saline (PBS, pH 7.4), and resuspended in PBS. Fixation was carried out by incubating the samples in Karnovsky's fixative (2.5% glutaraldehyde and 4% paraformaldehyde in 0.1 m sodium cacodylate buffer, pH 7.4) at 4°C for 8 h. After fixation, the samples were washed three times with cacodylate buffer and postfixed with 1% osmium tetroxide and 0.8% potassium ferrocyanide in cacodylate buffer for 1 h at room temperature. The postfixed samples were washed again three times, placed onto polylysine‐coated coverslips, and dehydrated through a graded acetone series (30%, 50%, 70%, 90%, 100%). Critical point drying was performed via a CPD‐030 critical point dryer (BAL‐TEC, USA). The samples were then sputter‐coated with a thin layer of gold‐palladium via a Balzers Union SCD‐040 sputter coater (Electron Microscopy Sciences, USA) and imaged via a JSM‐7001F SEM. Comparative analysis of untreated *Mtb*, DC05‐treated *Mtb*, and MSN‐CPP@DC05‐treated *Mtb* allowed visualization of structural alterations such as cell wall disruption, surface irregularities, and possible morphological deformation induced by the treatments.

### 2.6 In Vivo Assays

#### 2.6.1 Toxicity in *Galleria Mellonella* Larvae

The toxicity assay in *Galleria mellonella* was adapted from Allegra et al. [54]. Larvae weighing approximately 0.2 g were selected for the administration of 10 µL doses of the compounds of interest, as well as PBS for the control group, by injection into the lower left hemocoel via precision microsyringes (Hamilton Ltd.). All nanoparticle‐based groups (bare MSN, MSN‐CPP, MSN@DC05, MSN‐CPP@DC05) were evaluated at a dose of 2000 mg kg^−1^ of nanoparticles, calculated on larval body weight. Given the average larval weight (0.2 g), this dose corresponds to 400 µg of nanoparticles per larva. For MSN‐CPP@DC05, the loading capacity (37.8 mg DC05 g^−1^ MSN) results in an effective peptide dose of approximately 15.1 µg of DC05 per larva (75.6 mg kg^−1^). The group treated with free DC05 received the same peptide dose, 15.1 µg per larva (75.6 mg kg^−1^), dissolved in PBS without nanoparticles. Each formulation was administered to groups of 10 larvae, with three replicates per treatment. Larvae were incubated at a controlled ambient temperature (28°C), and mortality was recorded at 24, 48, 72, and 96 h post‐injection. The parameters assessed to determine toxicity included melanization, mobility, and mortality of the larvae, allowing for a comprehensive evaluation of the effects of the compounds on the viability and physiological responses of the larvae.

### 2.7 Statistical Analysis

All the experiments were conducted in triplicate, and the statistical analyses were performed via GraphPad Prism software, version 8.2.1 and OriginPro, Version 2025. OriginLab Corporation, Northampton, MA, USA. Tukey's test was applied to compare means when the overall *p* value of the experiment was below the significance threshold (*p* < 0.05). For the analysis of efflux pump inhibition, one‐way ANOVA was used, with a significant difference set at *p* < 0.0001.

## 3 Results and Discussion

### 3.1 ML‐Drug Design Based on Plectasin Analogs

Plectasin is a 40–amino acid peptide known for its activity against gram‐positive bacteria, and several of its derivatives have also shown activity against *Mtb* [43]. Its relatively large size, however, complicates chemical synthesis and increases production costs. To address this, 3D structural modeling was used to identify the regions of the peptide that contribute most directly to its activity against *Mtb*. The analysis indicated that Plectasin contains two conserved elements, a β‐sheet and an α‐helix (Figure S1). When the complete sequence was evaluated computationally, the predicted activity against *Mtb* was low, with a score of −0.47. In contrast, fragment analysis showed that the β‐sheet alone reached a score of 0.37, whereas the α‐helix scored −1.24. Negative values reflect an absence of activity, while scores above 0.5 are associated with strong antimicrobial potential. These observations guided the decision to modify the β‐sheet region in order to enhance activity while shortening the molecule. Further in silico work introduced substitutions at cysteine and glycine residues to reduce the likelihood of unwanted cyclization or dimer formation, issues frequently encountered in peptide engineering (**Annex 1**). More than ten thousand variants were generated, and those with the most favorable predictions for antimicrobial activity, general bioactivity, and *Mtb*‐focused potential were selected for synthesis (Table 1).

**TABLE 1:** Peptide sequences selected for synthesis since the ML results.

Selected sequences				
Code	Sequence	Anti‐*Mtb*	Bioactivity	Antimicrobial activity
DC05	GWYRAKRGFVWKRY	1.819	0.783	0.965
DC08	GEYRAKRGFVWKRY	1.705	0.613	0.867
DC09	GRYRAKEGFVWKRY	1.576	0.617	0.585
DC10	GRYRAKMGFVWKRY	1.629	0.663	0.576
Plectasin	GFGCNGPWDEDDMQCHNHCKSIKGYKGGYCAKGGFVCKCY	−0.47	0.999	0.900

DC05 was of particular interest owing to modifications at position eight (arginine in DC05). These changes significantly enhanced their antimicrobial properties. According to Sant et al. [55], leucine‐rich peptides have a relatively high affinity for lipid bilayers and can disrupt bacterial membranes effectively. However, this hydrophobicity also increases the degree of cytotoxicity. In contrast, arginine contributes to stronger electrostatic and hydrogen bonding with lipid phosphate heads, improving bacterial membrane disruption [56].

### 3.2 Is DC05 a Promising Analog IN SILICO?

**FIGURE 1:**
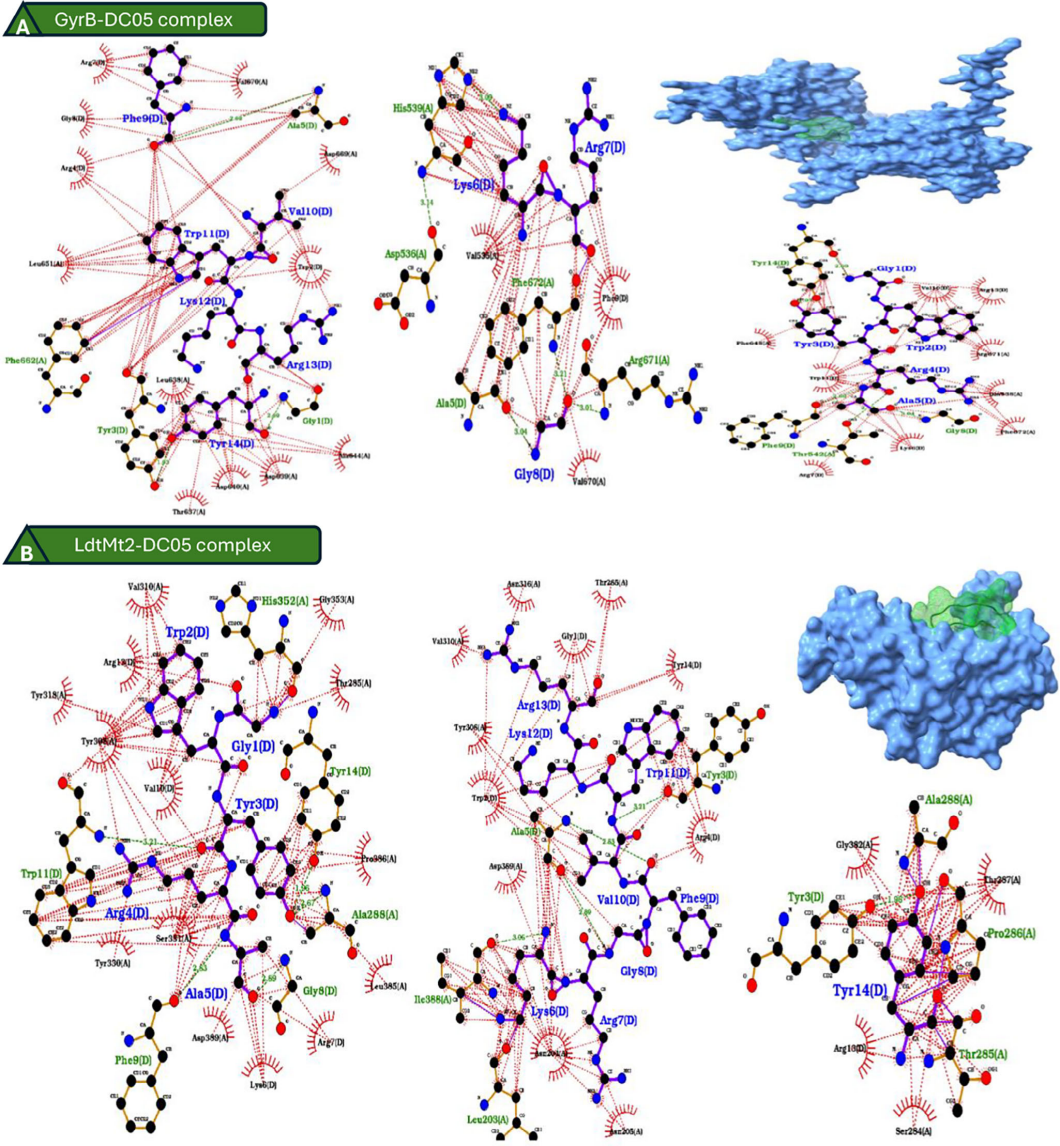
Interaction analysis of DC05 with *Mtb* targets. (A) GyrB–DC05 complex and (B) LdtMt2–DC05 complex display extensive hydrogen bonding and hydrophobic interactions.

The model selected from I‐TASSER provided a DC05 structure characterized by a C score of −1.11. On the other hand, the structure generated by AlphaFold3 presented pLDDT values above 90 for most residues, with slightly lower confidence for GLY1 [38, 68, 69, 70, 71, 72, 73, 74, 75, 76, 77, 78, 79, 80, 81, 82, 83, 84, 85, 86, 87] and a maximum PAE of 7 Å for the same residue. Ramachandran plot analysis revealed that the AlphaFold3 model had approximately 90% of the residues in the most favored or additionally allowed regions (Figure S2), and it was therefore selected for subsequent docking and interaction studies (Tables S1 and S2). Energy minimization was conducted via the Amber force field in Avogadro. DC05 had the highest ΔG value for LdtMt2 (−10.5 kcal/mol) and GyrB (−10.0 kcal/mol), suggesting strong and specific interactions with these targets, and the binding pocket residues and affinity values are presented in Table S3. Detailed molecular interaction analysis revealed that DC05 formed 25 stabilizing contacts with GyrB and 20 with LdtMt2 (Table S4), involving hydrogen bonds, hydrophobic contacts, salt bridges, and pi‐cation interactions. Individual receptor–peptide interactions are provided in Table S5, and the key peptide residues contributing to these interactions included Trp2, Arg4, Lys12, Arg13, and Tyr14, as summarized in Table S6. 2‐ and 3D visualizations of the top receptor‒peptide complexes were generated via PyMOL, LigPlot+, and ChimeraX (Figure 1; Figure S3). These models help visualize key binding contacts and peptide orientation within the receptor pockets.

The superior structural model from AlphaFold3, validated via Ramachandran plots and G factor analysis, laid a robust foundation for reliable docking predictions. Among all the receptors, GyrB exhibited the most extensive and stable interaction network, including 13 hydrophobic contacts, 9 hydrogen bonds, and multiple *π*‐cation and salt bridge interactions. The involvement of Tyr14 as a central anchoring residue for hydrophobic stacking and Trp2 in *π*‐cation and hydrogen bond interactions indicates that these residues are critical for receptor affinity and should be preserved in future analogs. In contrast, LdtMt2, although lacking salt bridges, formed strong hydrophobic and H‐bond networks through Pro286, Tyr308, and Asn204, resulting in the best binding score. The absence of certain interactions suggests that its binding strength is driven more by geometric complementarity and van der Waals stabilization.

Interestingly, EmbC presented two salt bridges and many hydrogen bonds, yet the predicted ΔG was lower, which emphasizes the importance of the interaction position and geometry, not just the quantity. These findings also reveal that residues such as Trp2, Arg4, Lys12, Arg13, and Tyr14 act as functional hotspots across all complexes, contributing to hydrophobic, electrostatic, and directional hydrogen bond networks. This consistency highlights their importance in maintaining broad‐spectrum activity while preserving specificity. The flexibility of LightDock's GSO algorithm allowed exploration of alternative docking poses beyond rigid body constraints, revealing previously unreported binding pockets. The divergence from earlier pocket definitions (e.g., Primo et al., [29]) may also reflect dynamic conformational changes relevant to physiological conditions.

LdtMt2 plays a critical role in the formation of 3‐3 cross‐links in the peptidoglycan layer of the mycobacterial cell wall, a structural feature particularly enriched during latent infection, and the inhibition of this enzyme compromises the integrity and stability of the bacterial cell wall, increasing the susceptibility of the pathogen to external stress and antibiotics [57]. The interaction of DC05 with LdtMt2 therefore suggests a potential bactericidal effect through disruption of cell wall biosynthesis, highlighting its relevance in combating persistent or drug‐tolerant forms of *Mtb*. Moreover, the observed interaction between DC05 and GyrB supports a complementary mechanism of action. GyrB is an essential component of the DNA gyrase complex that is responsible for DNA supercoiling and replication and is a validated target of fluoroquinolone antibiotics, such as moxifloxacin, and inhibiting GyrB function interferes with the bacterial replication process, effectively halting proliferation [58]. These observations indicate that DC05 may be able to engage both structural and functional pathways in *Mtb*, although this interpretation remains hypothetical. Additional biochemical validation, target‐specific inhibition assays and intracellular infection models are still required to determine whether these predicted interactions translate into measurable antibacterial effects or influence resistance development.

The in silico pharmacokinetic properties (Table S7) show that all the selected analogs exhibit low intestinal permeability (0% absorption), a common characteristic of therapeutic peptides due to their size and physicochemical properties [59]. Despite this, all analogs are substrates for P‐glycoprotein, which could influence their bioavailability when P‐glycoprotein is administered systemically [60]. However, this interaction could be exploited to enhance site‐specific action. The distribution characteristics are also notable: all analogs show low permeability across the BBB and CNS, an advantageous feature for avoiding potential off‐target effects. Additionally, the low unbound fraction in plasma suggests a prolonged release profile, which could be beneficial for dosing in chronic infections such as TB [61].

The predicted half‐lives in the biological models presented in Table S8 indicate that analogs DC05 and DC09 have prolonged half‐lives in mammalian reticulocytes (30 h) and yeast and *E. coli* models (over 20 and 10 h, respectively). This stability is essential for controlling peptide activity against *Mtb*, justifying their selection for further studies. In contrast, analogs DC08 and DC10 have shorter half‐lives in prokaryotic and eukaryotic environments, which could limit their effectiveness in long‐term therapies. However, this shorter half‐life may be advantageous in treatments where rapid clearance is desired post therapy to reduce toxicity [62].

### 3.3 Antimicrobial Activity and Biocompatibility of Analogs

**FIGURE 2:**
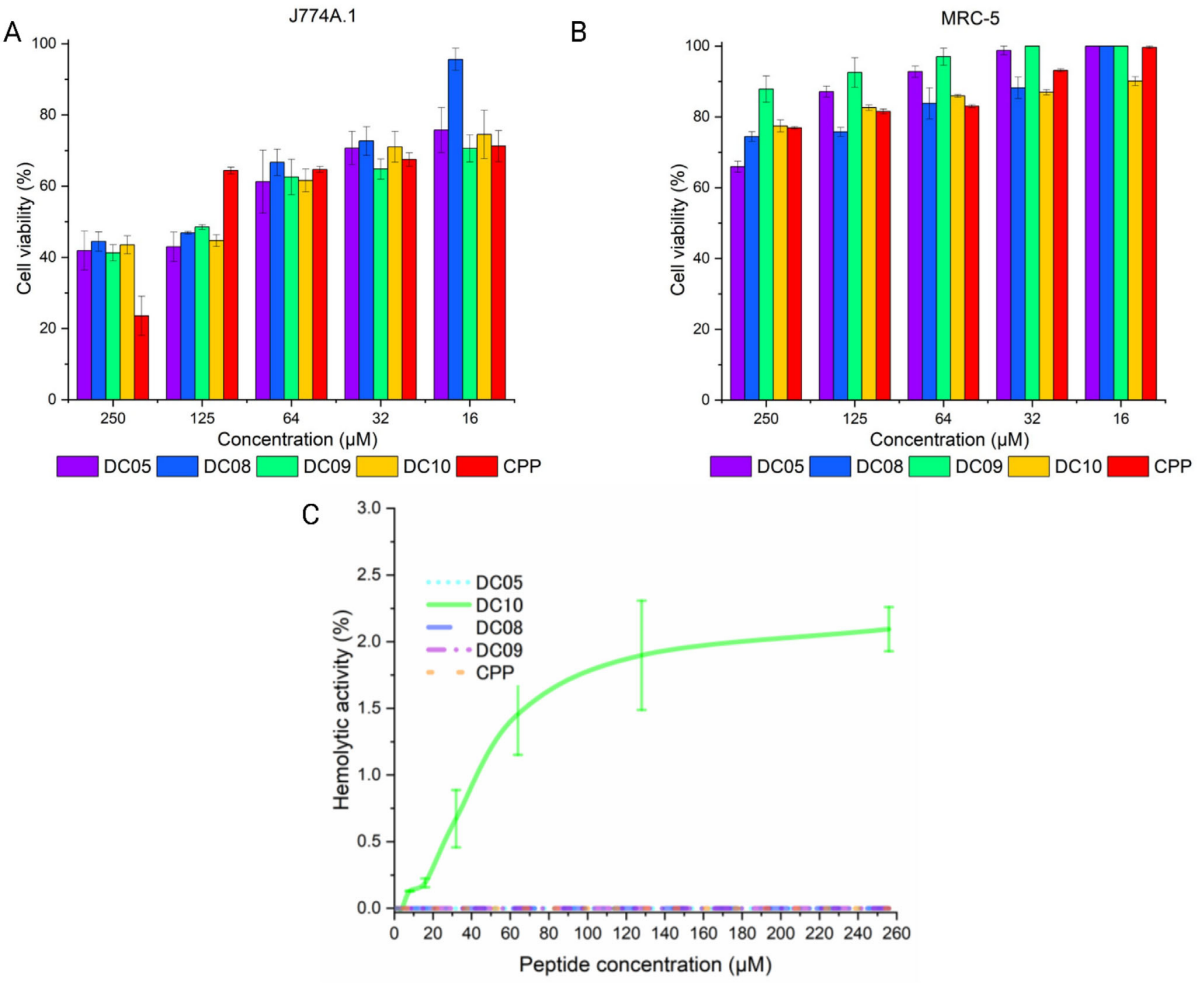
Cytotoxicity evaluation of AMPs and CPPs in (A) J774A.1 macrophages, (B) MRC‐5 fibroblasts, and (C) human erythrocytes. Cells were exposed to 16–250 µm of peptides (DC05, DC08, DC09, DC10 and CPP). Viability was determined via resazurin assays in macrophages and fibroblasts, while hemolytic activity in erythrocytes was measured by absorbance. Data are expressed as a percentage relative to untreated control cells and represent the mean values from three independent experiments (*n* = 3).

Analysis of the designed peptides (Figure S3) was performed against *Mtb* H37Rv and two mammalian cell lines (MRC‐5 and J774A.1). The results revealed differences in both antimicrobial potency and selectivity. DC05 displayed the most favorable profile, with an MIC_90_ of 12.46 ± 0.06 µm and low cytotoxicity (IC_50_>250 µm in human fibroblasts and 115.18 ± 8.49 µm in macrophages) (Figure 2A,B). Its selectivity index (SI) was >20.05 in MRC‐5, exceeding the high‐selectivity threshold (SI >10) proposed by Campos et al. [63], and 9.24 ± 0.68 in J774A.1, which is above the SI >2.5 value considered acceptable according to Elisha et al. [64].

To contextualize the antimicrobial potency, Campos et al. [63] reported MIC_90_ values of 5.12 ± 1.71 µm for amikacin, 6.93 ± 3.14 µm for isoniazid, and 1.48 ± 0.59 µm for linezolid, whereas Degiacomi et al. [65] reported a MIC_90_ of 0.108 µm for bedaquiline under comparable experimental conditions. Although these reference drugs remain more potent than the peptides evaluated here, the transition from an inactive peptide to an analog with a MIC_90_ of 12.46 µm indicates that the structural modifications introduced were effective in generating measurable activity against *Mtb*. This creates a basis for further fine‐tuning of the sequence to reduce the MIC_90_, and it is reasonable to expect that nanoformulation strategies could further improve potency, as documented for other peptide‐based systems.

Among the other peptides, DC10 showed an SI of >10.41 in MRC‐5 and 8.79 ± 0.65 in J774A.1, indicating a good therapeutic window; the main difference from DC05 lies in its lower antimicrobial potency (MIC_90_ 24 vs 12.46 µm). DC08 and DC09 exhibited SI values of >6.76 and >5.10 in MRC‐5, respectively, whereas their values in macrophages (6.21 ± 0.36 and 4.83 ± 0.30) reflected moderate selectivity. Finally, CPP, another analog, displayed an intermediate profile, with an undefined SI in MRC‐5 and ≤4.12 in J774A.1, which was consistent with moderate selectivity (Table 2). Compared with the literature, it is worth noting that most classical antimicrobial peptides, such as CAMEL, temporin A, pexiganan, or LL‐37, generally exhibit much lower selectivity indices, often <2 even in optimized formulations [66]. For example, Sikora et al. [66]. reported maximum SI values of 1.25 and 1.34 for CAMEL and temporin A, respectively, underscoring the inherent challenge of developing peptides that are both effective and selective. Overall, DC05 clearly exceeds the selectivity benchmarks described for most AMPs and has emerged as a peptide with high pharmaceutical potential, particularly in applications where cytotoxicity represents a major limitation. Nevertheless, further sequence optimization is required to improve its MIC_90_.

**TABLE 2:** Antitubercular activity, cytotoxicity in mammalian cells, and selectivity indices for peptides. All values are expressed in micromolar (µm).

AMPs	H_37_Rv	IC_50_ MRC‐5	IC_50_ J774A.1	SI MRC‐5	SI J774A.1
DC05	12.46 ± 0.06	>250	115.18 ± 8.49	>20.05	9.24 ± 0.68
DC08	37 ± 2.07	>250	229.78 ± 4.08	>6.76	6.21 ± 0.36
DC09	49 ± 2.89	>250	236.77 ± 4.57	>5.10	4.83 ± 0.30
DC10	24 ± 1.72	>250	211.08 ± 3.63	>10.41	8.79 ± 0.65
CPP	>50	>250	205.9 ± 6.5	n.d	≤ 4.12

SI: Selectivity index, n.d. = not determined.

The DC10 (GRYRAKMGFVWKRY‐NH_2_), which contains a methionine residue in its sequence, exhibited a slight increase in hemolytic activity (2.08% ± 0.15%), whereas all variants with substitutions at this position displayed 0% hemolysis. This result suggests that methionine, by contributing hydrophobic characteristics, promotes less selective interactions with erythrocyte membranes and reduces the discrimination between bacterial and eukaryotic membranes [67, 68]. This finding is in line with the report by Lata et al. [69], who reported that in the peptide YRA‐15, the presence of methionine at critical positions increased hydrophobicity and was associated with increased cytotoxicity and hemolysis, whereas its substitution with fewer hydrophobic residues significantly reduced these effects without compromising antimicrobial activity.

The minimal hemolysis observed for the DC05, DC08, DC09 and CPP, despite their positive net charge, is particularly promising (Figure 2C). The ability of these peptides to discriminate selectively between bacterial and erythrocyte membranes is likely due to their optimized balance of hydrophilic and hydrophobic regions, which allows targeted disruption of bacterial membranes without compromising host cell integrity [70]. This selectivity is critical for clinical translation, as it reduces the risk of off‐target effects and potential side effects associated with peptide therapies. Given these observations, DC05 has emerged as a strong candidate for further investigation and development because of its potent antimycobacterial activity and outstanding hemolytic safety profile [71].

### 3.4 Ethidium Bromide Accumulation Indicates Efflux Pump Inhibition

**FIGURE 3:**
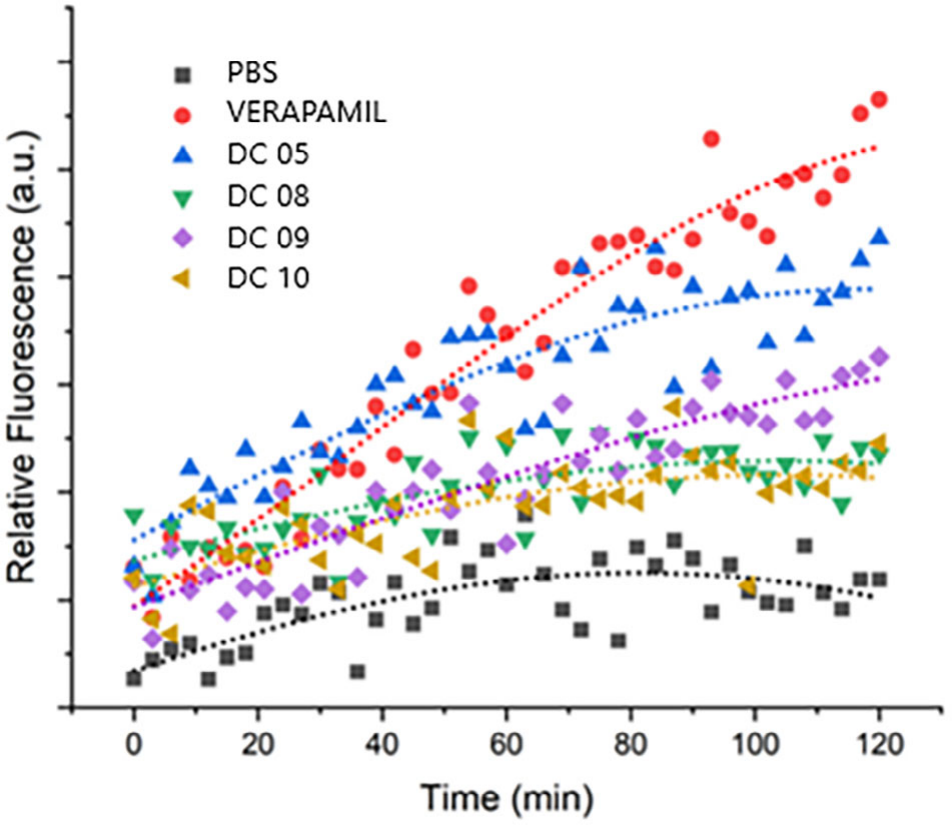
Ethidium bromide (EtBr) accumulation assay evaluating the efflux pump inhibitory activity of AMPs (DC05, DC08, DC09 and DC10) and verapamil (positive control) against *Mtb*. The relative fluorescence intensity was measured over 120 min, indicating that intracellular EtBr accumulation occurred because of efflux pump inhibition. Higher fluorescence signals correlate with increased inhibition of efflux activity. Data represent the mean values from three independent experiments (*n* = 3).

Efflux pumps significantly contribute to bacterial resistance by actively expelling antibiotics and other therapeutic agents from bacterial cells, thereby reducing their intracellular concentrations and overall efficacy [72]. In this study, the efflux pump inhibitory activity of the DC05, DC08, DC09, and DC10 was investigated via EtBr accumulation via a reporter assay (Figure 3). EtBr is a recognized substrate of various bacterial efflux pumps, particularly those belonging to the ATP‐binding cassette (ABC) and resistance‐nodulation‐cell division (RND) transporter families, which are known to be widely distributed and functionally significant in gram‐negative bacteria and mycobacteria, including *Mtb* [73, 74]. In mycobacteria, efflux pump inhibition is evaluated by observing changes in intracellular EtBr accumulation, which significantly fluoresces upon intercalation with bacterial DNA [75]. The enhanced fluorescence thus indicates successful inhibition of efflux activity, as more EtBr remains available intracellularly to interact with bacterial nucleic acids. Figure 3 shows that DC05 markedly increased intracellular EtBr accumulation in *Mtb* cells, indicating robust inhibition of efflux pumps, with effectiveness closely comparable to that of verapamil, a well‐established efflux inhibitor known to target primarily ABC transporters.

This pronounced inhibition suggests that DC05 likely interacts with and disrupts the activity of mycobacterial ABC efflux pumps, which are prominently implicated in antibiotic resistance mechanisms. DC10 displayed moderate inhibitory effects, which, while notable, were inferior to those of DC05, and DC08/DC09 demonstrated only mild inhibitory potential, suggesting limited interactions with efflux pumps or differential targeting specificity. Understanding the precise molecular interaction between DC05 and ABC‐ or RND‐type transporters could inform future optimization, potentially allowing rational design strategies for enhancing specificity and therapeutic potency.

Further studies should employ molecular modeling and targeted inhibition assays to definitively identify the specific efflux pump targets of DC05. Detailed mechanistic investigations will provide valuable insights into peptide‒efflux pump interactions and their implications for overcoming antibiotic resistance. In summary, the demonstrated efflux pump inhibitory activity positions DC05 as an attractive candidate for further therapeutic development, especially in combination treatments for combating multidrug‐resistant *Mtb*.

### 3.5 DC05 Mutagenicity Assessment

The mutagenic potential of DC05 was assessed via the Ames test, a standard reverse mutation assay employing the bacterial strains *Salmonella* Typhimurium TA97a, TA98, TA100, and TA102, which possess defined mutations sensitive to different classes of mutagens. The results presented in Table 3 demonstrate that DC05 did not induce a statistically significant increase in revertant colonies across all the tested concentrations (25–200 µm) compared with the negative controls, both in the presence (+S9) and absence (‐S9) of metabolic activation. The mutagenicity index (MI), a critical indicator where a value equal to or greater than 2 typically indicates mutagenic activity, consistently remained below this threshold for DC05, ranging from 0.65 to 1.23. The absence of dose‐dependent trends in the number of revertants further supports the conclusion that DC05 lacks mutagenic activity, reinforcing its favorable safety profile. The slight fluctuations observed across concentrations can be attributed to inherent biological variability, which is common in assays involving bacterial metabolic and replication processes. Additionally, the inclusion of metabolic activation (the S9 fraction derived from rat liver microsomes) did not significantly alter revertant frequencies, confirming that DC05 metabolites, if produced, do not possess mutagenic potential.

**TABLE 3:** Mutagenic activity is expressed as the mean and standard deviation of the number of revertants/plate and mutagenicity index in S. Typhimurium strains TA97a, TA98, TA100, and TA102 after treatment with different concentrations of DC05 in experiments without (−S9) and with (+S9) metabolic activation.

Number of revertants (M±SD)/plate and MI								
Treatments	TA97a		TA98		TA100		TA102	
	−S9	+S9	−S9	+S9	−S9	+S9	−S9	+S9
C‐	136 ± 12	140 ± 26	27 ± 4	20 ± 6	91 ± 13	108 ± 19	296 ± 42	299 ± 41
C+	1439 ± 102^a^	1228 ± 140^d^	682 ± 75^a^	837 ± 53^d^	1109 ± 118^b^	1301 ± 120^d^	1042 ± 99^c^	1423 ± 81^e^
DC05 (µm/plate)								
25	140 ± 11 (1.03)	164 ± 27 (1.17)	30 ± 6 (1.11)	22 ± 4 (1.08)	102 ± 18 (1.12)	112 ± 24 (1.04)	344 ± 26 (1.16)	368 ± 49 (1.23)
50	138 ± 18 (1.01)	150 ± 12 (1.07)	28 ± 5 (1.04)	22 ± 6 (1.10)	104 ± 11 (1.14)	120 ± 18 (1.11)	300 ± 18 (1.01)	350 ± 28 (1.17)
100	124 ± 22 (0.91)	122 ± 20 (0.87)	27 ± 1 (0.98)	21 ± 4 (1.05)	93 ± 24 (1.02)	114 ± 12 (1.06)	302 ± 31 (1.02)	316 ± 39 (1.06)
150	131 ± 10 (0.96)	112 ± 11 (0.80)	29 ± 2 (1.07)	20 ± 2 (0.98)	99 ± 8 (1.09)	110 ± 11 (1.02)	242 ± 25 (0.82)	292 ± 31 (0.98)
200	98 ± 8 (0.72)	102 ± 18 (0.73)	28 ± 6 (1.04)	21 ± 5 (1.03)	76 ± 13 (0.83)	82 ± 6 (0.76)	192 ± 30 (0.65)	220 ± 23 (0.74)

* *p* < 0.05 (ANOVA), M ± SD = mean and standard deviation; MI = mutagenicity index; Negative control: dimethyl sulfoxide (DMSO—100 µL/plate); C+ = positive control.

^a^ 4‐nitro‐o‐phenylenediamine (TA98 and TA97a, 10.0 µg/plate);

^b^ sodium azide (TA100, 1.25 µg/plate);

^c^ mitomycin (TA102, 0.5 µg/plate), in the absence of S9;

^d^ 2‐anthramine (TA98, TA100, TA97a, 1.25 µg/plate);

^e^ 2‐aminofluorene (TA102, 10.0 µg/plate), in the presence of S9.

The strains used in this evaluation specifically detect different classes of mutagens: TA98 detects frameshift mutations, TA100 identifies base‐pair substitutions, and TA102 is particularly sensitive to oxidative mutagens and DNA intercalating agents [76, 77]. The lack of mutagenic activity observed for DC05 is important for therapeutic development, particularly given its intended use against resistant pathogens such as MDR *Mtb*. Therapeutic agents targeting such severe infections must demonstrate not only high antimicrobial efficacy but also excellent safety profiles to ensure patient safety and compliance [78]. DC05 meets both criteria, showing robust antimicrobial and efflux pump inhibitory activities, combined with negligible cytotoxicity and no mutagenic risk. These findings substantially enhance the preclinical profile of DC05, supporting its potential progression to further stages of drug development, including detailed in vivo studies and eventual clinical trials. Future evaluations might consider more comprehensive genotoxicity assays, such as mammalian cell gene mutation tests or in vivo comet assays, to further confirm genomic safety [79].

### 3.6 MSN as a Promising Therapy Against *Mtb*

#### 3.6.1 Physicochemical Characterization

**FIGURE 4:**
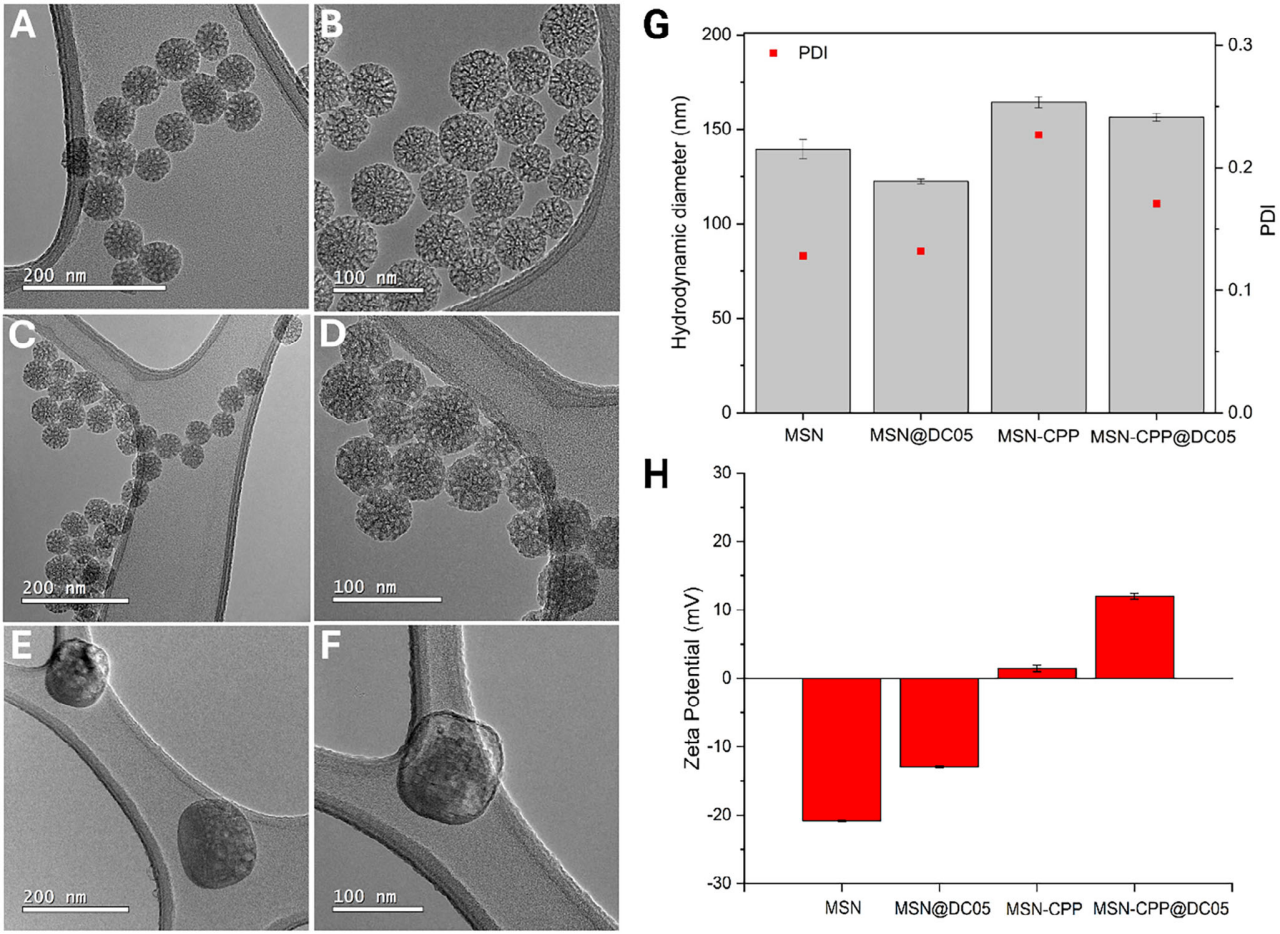
HR‐TEM images of MSN (A, B), MSN@DC05, (C, D), MSN‐CPP (E, F) at different magnifications. (G) Hydrodynamic diameter in SBF medium at 0.5 mg mL^−1^. (H) ZP of the nanoplatforms in HEPES 10 mm pH 7.0 (concentration: 0.5 mg mL^−1^, *n*  =  3).

The MSN were fabricated as previously described and characterized via high‐resolution transmission electron microscopy (HR‐TEM) (Figure 4A,B). MSN exhibits homogeneous spherical morphology, monodisperse with a well‐defined mesopores network, and no evidence of aggregation or structural collapse, which is essential for ensuring reproducibility and the success of subsequent peptide functionalization steps. These observations are consistent with previous reports indicating that MSN retain uniform morphology, stable mesostructure, and high porosity and surface area, characteristics that support their performance as nanocarriers in biomedical systems [38, 80, 81].

Dynamic light scattering analysis in simulated body fluid at 0.5 mg mL^−1^ showed that all nanoplatforms remained within a constrained hydrodynamic size interval. Bare MSN exhibited a mean hydrodynamic diameter of 139.6 ± 5.2 nm, whereas MSN@DC05 measured 122.5 ± 1.2 nm, MSN‐CPP 164.3 ± 2.9 nm and MSN‐CPP@DC05 156.4 ± 2.1 nm. The corresponding polydispersity indices were 0.128 ± 0.041, 0.132 ± 0.005, 0.227 ± 0.051 and 0.171 ± 0.017, respectively, consistent with single, well‐defined particle populations and absence of marked aggregation in SBF. Additional measurements in 10 mm HEPES, are provided in Figure S4. This increase in particle size is consistent with the incorporation of components onto the surface [41]. The literature further highlights that nanoparticle size plays a critical role in cellular uptake efficiency, biodistribution, and blood circulation time [82, 83]. Particles within the 100–250 nm range are considered optimal for biomedical applications that require efficient cellular internalization and prolonged retention, minimizing rapid renal clearance and enhancing the enhanced permeability and retention (EPR) effect in target tissues [83, 84]. In fact, particles smaller than 6 nm tend to be rapidly filtered by the kidneys, whereas those above 200–250 nm may be cleared by the mononuclear phagocyte system. Several studies have reported that maximum cellular uptake for functionalized MSN typically occurs with particles between 40 and 150 nm, although particles up to 250 nm can still maintain efficient internalization and enhance drug loading and release profiles [80, 82].

Zeta potential (ZP) analysis was used to monitor surface‐charge variations on the MSNs throughout the functionalization steps (Figure 4H). Pristine MSNs exhibited a markedly negative zeta potential (–20.9 ± 0.1 mV), attributable to the abundance of deprotonated silanol groups (–SiO–) on the surface, a typical feature of native silica that contributes to colloidal stability in aqueous media [85, 86]. After incubation with the cationic peptide DC05 (MSN@DC05), the ZP shifted to –13.0 ± 0.2 mV. This partial neutralization is consistent with electrostatic interactions between positively charged DC05 residues and negatively charged silanols, in agreement with previous reports describing the adsorption of cationic peptides onto silica surfaces [87]. A more pronounced shift was observed after CPP conjugation, as MSN‐CPP displayed a slightly positive zeta potential (+1.4 ± 0.5 mV). Literature reports indicate that grafting cationic peptides can shift the net surface charge toward neutral or positive values and facilitate interactions with cellular membranes [88]. Dual functionalization and loading (MSN‐CPP@DC05) further increased the ZP to +12.0 ± 0.4 mV, reflecting the cumulative contribution of both peptide components to the overall surface charge. Complementary FTIR spectra for MSN, MSN@DC05, MSN‐CPP and MSN‐CPP@DC05 are shown in Figure S5

**FIGURE 5:**
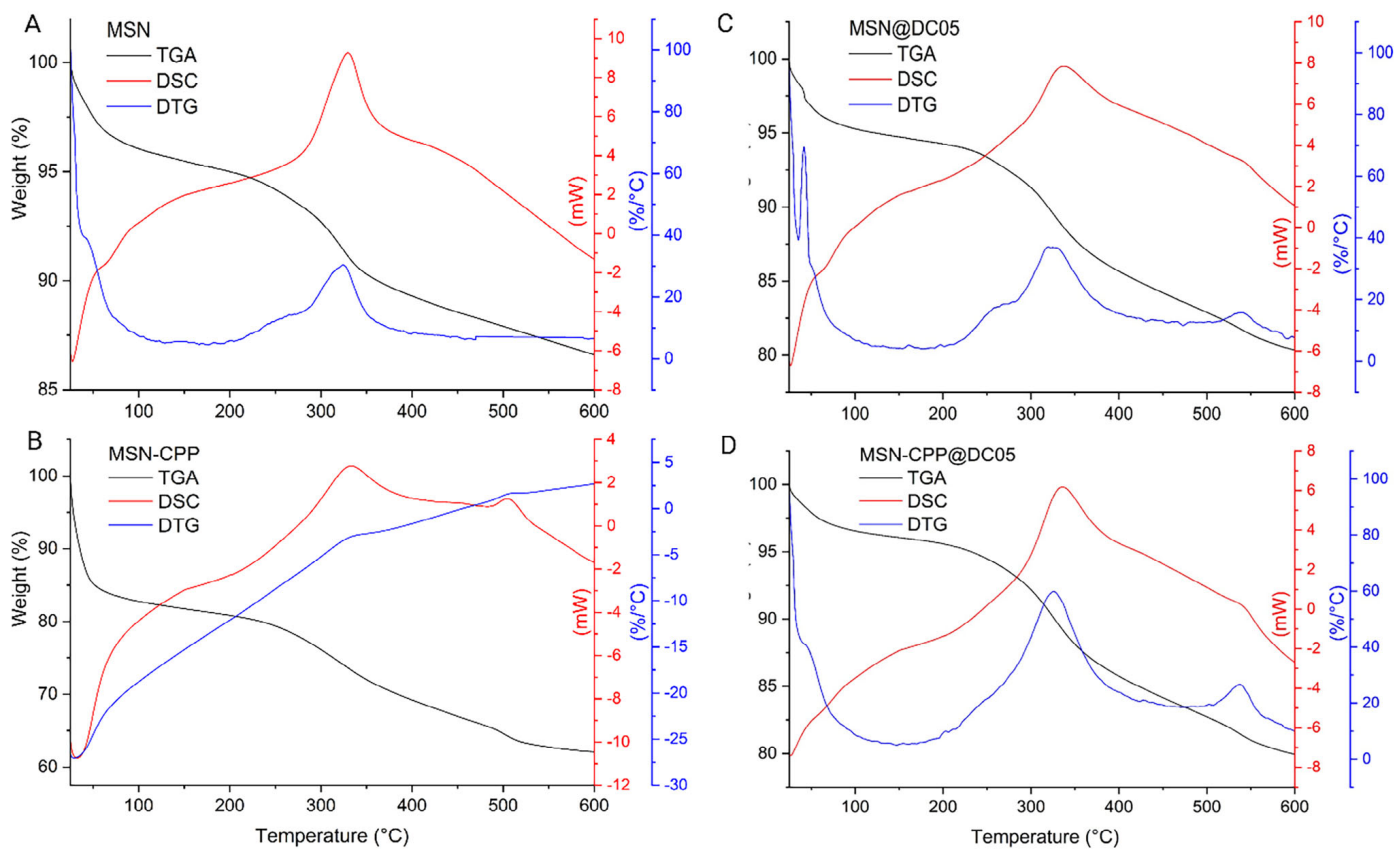
(A–D) Thermogravimetric analysis (TGA, black lines), derivative thermogravimetry (DTG, blue lines), and differential scanning calorimetry (DSC, red lines) profiles of MSN (A), MSN‐CPP (B), MSN@DC05 (C), and MSN‐CPP@DC05 (D).

The TG and DSC analysis (Figure 5A–D) revealed three thermal regions: (i) moisture desorption below 120°C, (ii) release of bound water and onset of organic decomposition between 120°C and 300°C, and (iii) pyrolysis/oxidation of organic residues and carbonization above 300°C. In DSC results the main features appear between 200°C and 600°C as overlapping exothermic events, consistent with oxidative degradation and pyrolysis of organic moieties.

In pristine MSN (Figure 5A, blue and black lines), the water loss below 120°C corresponds to the removal of physically adsorbed water (3%–4%) [89]. In the 200°C–300°C range, a minimal mass loss was observed, attributed to residual organic content from surfactant traces [90], while the remaining signal was associated with partial silica dehydroxylation (between 200°C and 600°C) corresponding to 8%–9% of mass loss (Figure 5A, blue and black lines).

For MSN‐CPP (Figure 5B, blue and black lines), the overall mass loss became more pronounced. The initial decrease (<120°C, 10%–12%) was associated with superficially bound water; between 120°C and 200°C, an additional 5%–6% loss was detected due to weakly bound water through hydrogen bonding or coordination; and between 200°C and 600°C, a significant fraction (18%–19%) was released, corresponding to strongly retained water overlapping with the onset of organic decomposition. The maximum peaks at 333°C and 510°C reflected the pyrolysis of free MPTMS groups and surface‐bound CPP [91] (Figure 5B, blue and black lines). This differential behavior can be rationalized considering the nature of CPP (Tuftsin‐Cys; Thr‐Lys‐Pro‐Arg‐Ahx‐Cys), whose sequence contains polar and charged residues (ε‐NH_3_^+^ of Lys, guanidinium of Arg, ‐OH of Thr, ‐SH of Cys) capable of establishing multiple interactions with water [92, 93]. Such interactions favor water retention in different states—free water (25°C–100°C), hydrogen‐bonded or coordinated water (100°C–200°C), and strongly retained water (>200°C)—resulting in a stepwise loss pattern and a shift of organic decomposition to higher temperatures, as evidenced by the maximum peak at 333°C [94, 95].

Regarding MSN@DC05 (Figure 5C, blue and black lines), water loss was reduced (<120°C, 4%–6%) and the main organic decomposition event between 200°C and 600°C (peaks centered at 325°C and 540°C) was assigned to the DC05 physically entrapped inside the pores (13%–14%). In addition, the MSN@DC05 exhibited reduced exposure to oxygen and lower molecular mobility, which shifted and broadened its thermal degradation compared with the free DC05, typically observed between 220°C and 250°C [96, 97].

In the MSN‐CPP@DC05 (Figure 5D, blue and black lines), water loss was lower than in the solely CPP‐functionalized MSN (<120°C, 6%–8%; 120°C–200°C, 3%–4%), and the degradation profile became more complex. A wide decomposition event between 200°C and 600°C with two maximum peaks at 325°C and 538°C can be attributed to the combined contribution of MPTMS, CPP and DC05. This result is consistent with a higher peptide fraction and the presence of peptides in distinct microenvironments, such as surface and pores [96, 97, 98]. The mass loss assigned to the peptides decomposition was 15%–16% (Figure 5D, blue and black lines). Comparison of the organic fractions referenced to the dry mass at 120°C shows that the incremental mass gain on going from MSN to MSN@DC05 is larger than the increment from MSN‐CPP to MSN‐CPP@DC05. This trend supports the lower encapsulation efficiency, loading capacity and Langmuir q values obtained for DC05 in MSN‐CPP@DC05 compared with MSN@DC05, and indicates that CPP functionalization reduces the maximum amount of DC05 that can be accommodated into the nanoplatform. A plausible explanation is the partial occupation of pore entrances and external surface by CPP, which reduces accessible surface area and pore volume and introduces steric hindrance and competition for adsorption sites. In the 350°C–600°C range, the thermal events for all peptide containing samples were associated with degradation of carbonaceous residues and strongly bound organic species. This pattern is consistent with the observations of Tengjisi et al. [99], who reported that peptide degradation in bioinspired silica nanoparticles is essentially complete around 600°C.

Furthermore, to confirm the successful fabrication of MSN‐CPP, Ellman's reagent assay was performed to quantify the free thiol groups accessible for conjugation. A significant difference was detected in thiol content between the CPP and MSN‐MPTMS groups, which highlights the impact of thiolation efficiency on subsequent peptide–nanoparticle interactions [48]. The free CPP presented a free thiol concentration of 5.78 ± 0.31 µg/mL, approximately six times greater than that of MSN‐MPTMS (0.93 ± 0.17 µg/mL). This considerable difference suggests that CPP retains a high density of reactive thiol groups, ensuring enough anchoring sites for conjugation onto MSN surfaces. The greater availability of free thiols in CPPs is crucial for optimizing conjugation efficiency, as it facilitates covalent attachment via disulfide bond formation or Michael addition reactions commonly used in thiol‐based surface chemistries [100].

In addition to thiol quantification, the efficiency of CPP conjugation onto MSN‐MPTMS was evaluated using an indirect approach. CPP concentration in the supernatant was quantified by a calibration curve, and the amount of CPP bound to the MSN was obtained by subtracting the supernatant value from the initial CPP concentration. Under these conditions, 782.18 ± 12.59 µg/mL CPP were calculated to be conjugated to the MSN surface, whereas 217.82 ± 12.59 µg/mL CPP remained in the supernatant, yielding a conjugation efficiency of 78.22 ± 1.26%. These results demonstrate that the conjugation process was highly efficient, allowing a substantial fraction of the CPP to be anchored onto the modified MSN surface, in agreement with previous reports showing that peptide‐functionalized nanoparticles exhibit enhanced conjugation yields and improved surface coverage [101]. The relatively low thiol concentration detected in MSN‐MPTMS confirmed that only a fraction of the MPTMS groups were surface‐exposed; nevertheless, the reactive thiols present were sufficient for effective peptide conjugation. The excess of free thiol groups in CPP also suggests that an optimized stoichiometric ratio between CPP and MSN‐MPTMS may be necessary to maximize conjugation efficiency while preventing steric hindrance or self‐polymerization.

Under the applied conjugation and loading conditions, the two peptides interact with distinct domains of the nanoplatform. CPP is the only peptide covalently grafted onto the external MSN‐MPTMS surface, reaching a grafting rate of 78.2 mg CPP g^−1^ MSN, whereas DC05 is incorporated through non‐covalent adsorption within the mesoporous network, achieving a loading capacity of 37.8 mg DC05 g^−1^ MSN at the condition used for biological assays (714 µg DC05 per 10 mg MSN‐CPP). These values confirm that CPP functionalizes the external surface while DC05 occupies the mesoporous interior, with no evidence of competitive displacement between peptides. TEM, DLS and TGA analyses further verified that the mesoporous architecture remains intact after dual functionalization, indicating that the nanoplatform preserves sufficient accessible volume to accommodate both peptides without steric competition.

#### 3.6.2 In Vitro Release Assays

**FIGURE 6:**
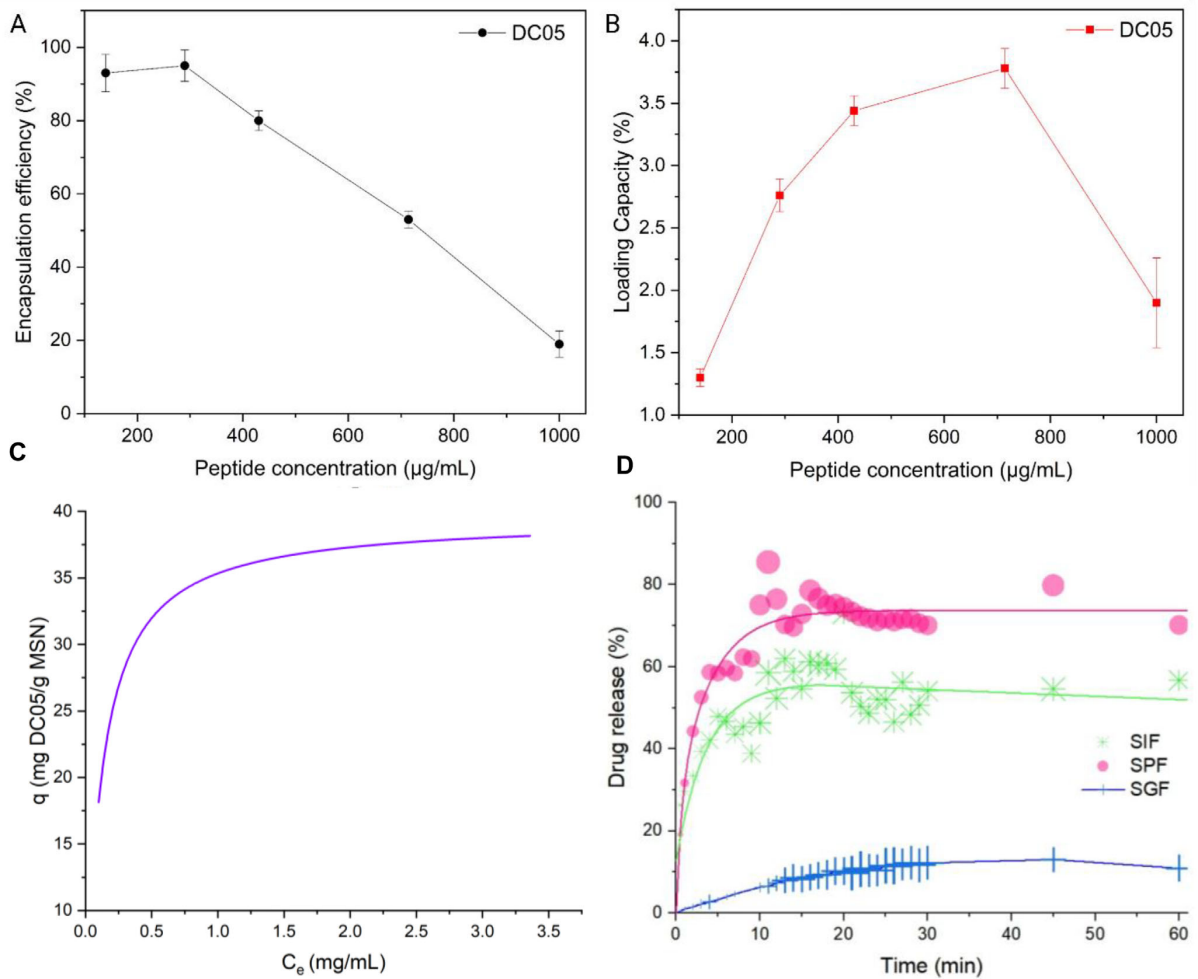
(A) Encapsulation efficiency as a function of DC05 concentration. (B) Loading capacity as a function of DC05 concentration (C) Langmuir adsorption isotherm of the peptide on MSN, showing the amount adsorbed per gram of MSN (𝑞) as a function of the equilibrium concentration of non‐adsorbed peptide (𝐶_𝑒_). Data were fitted to a two‐parameter Langmuir model to estimate 𝑞_max_ and 𝐾. The highest concentration point is shown but was not included in the fit. (D) Release profile of MSN‐CPP@DC05 in simulated biological environments: simulated pulmonary fluid (SPF, pH 7.4), simulated intestinal fluid (SIF, pH 6.8), and simulated gastric fluid (SGF, pH 1.2).

The EE% of the MSN‐CPP@DC05 was strongly dependent on the initial DC05 concentration. At low amounts (140–290 µg), the EE reached 93.0 ± 5.1% and 95.0 ± 4.3% Figure 6A). However, as the concentration increased, the EE progressively decreased, reaching 80.0% ± 2.7% at and 53.0% ± 2.3% at 430 and 714 µg, respectively, and decreased to a minimum of 19.0% ± 3.6% at 1000 µg. This reduction in EE% at higher concentrations suggests saturation of the available binding sites on the MSN surface [102]. Excess of peptide generates overcrowding effects, in which multiple molecules compete for limited adsorption sites, thereby reducing retention efficiency. Additionally, once a critical concentration is reached, steric hindrance and electrostatic repulsion between peptides may further hinder encapsulation [103]. In contrast, the loading capacity (LC%) exhibited a different behavior (Figure 6B). Starting from 1.30% ± 0.07% with 140 µg of DC05, the LC progressively increased, reaching a maximum of 3.78% ± 0.16% at 714 µg, which indicates the existence of an optimal concentration range that maximizes the DC05 content retained in the MSN. However, at 1000 µg, the LC decreased to 1.90% ± 0.36%, suggesting that excessive concentrations may induce adverse phenomena such as aggregation, surface desaturation, or structural destabilization of the nanoparticles [104]. Under such conditions, some of the weakly adsorbed peptides may be released into the supernatant, reducing the amount retained in the nanoplatform [102]. Overall, these results suggest that DC05 encapsulation in MSN follows a saturation‐dependent mechanism, in which an optimal concentration simultaneously maximizes both the EE% and the LC% before reaching a decrease associated with aggregation or desorption.

The adsorption profile of the peptide onto the MSN surface increased as a function of the initial peptide concentration, reaching a plateau at higher concentrations (Figure 6C). The calculated equilibrium concentrations (𝐶𝑒) ranged from near‐zero to 3.38 mg/mL, and the corresponding adsorption values (𝑞) varied from 13.0 to 37.8 mg/g MSN@DC05. The first four data points followed a saturation trend compatible with monolayer adsorption, whereas the highest concentration deviated from this behavior, consistent with surface saturation and partial peptide desorption. Fitting of the adsorption data to the Langmuir model yielded a maximum adsorption capacity (𝑞_max_) of 39.5 ± 4.9 mg/g MSN@DC05 and an affinity constant (𝐾) of 8.49 ± 4.26 mL/mg. The model accurately described the initial adsorption region, but did not reproduce the final data point, which is expected because the Langmuir formalism assumes a homogeneous distribution of adsorption sites and a single monolayer. At the highest concentration, competition between adsorbed molecules, partial multilayer formation, or rearrangement of peptide aggregates likely results in non‐Langmuir behavior, leading to the observed deviation. For this reason, only the concentration range consistent with monolayer adsorption was used to extract the Langmuir parameters. The Langmuir fit provided estimates of the 𝑞_max_ and the 𝐾, which describe the saturation limit and the concentration dependence of peptide binding within the monolayer region.

The release profile of MSN‐CPP@DC05 in three simulated biological environments (SPF, SIF, and SGF) revealed that the observed differences in release are primarily driven by the composition of the surrounding medium rather than by an intrinsic pH‐dependent release mechanism (Figure 6D). The fastest release occurs in SPF (pH 7.4, pulmonary conditions), where over 70% of the peptide is released within 10 min, suggesting high solubility and rapid desorption under pulmonary‐like conditions. In SIF (pH 6.8, intestinal conditions), release is slower (∼50% over 30 min), indicating that medium‐specific factors such as ionic strength and buffer composition influence peptide–nanoparticle interactions. While the low peptide release observed under acidic conditions (SGF, pH 1.2)—less than 10% even after 60 min—may suggest that the MSN offer some degree of protection, it is also plausible that a fraction of DC05 was released and rapidly degraded due to the harsh environment. Unlike native defensins such as plectasin, which exhibit remarkable acid stability due to their disulfide‐stabilized structure, DC05 is a linear derivative lacking disulfide bridges and is therefore more prone to acid‐induced degradation [105]. The minimal detection under these conditions may thus reflect both limited release and partial peptide breakdown. However, a dedicated stability study of DC05 in SGF was not performed in this work. For this reason, the SGF data are interpreted as apparent release profiles rather than a full mass balance of intact peptide.

The ability of MSN to modulate the apparent peptide release as a result of the surrounding medium composition, rather than through an intrinsic release‐control mechanism, presents a valuable strategy for targeted drug delivery, particularly for pulmonary and gastrointestinal applications [106, 107]. To evaluate whether the observed biological effects could be attributed to premature peptide release, particle‐free supernatants obtained after short incubation periods were tested on macrophages. No significant cytotoxic effects were observed (Figure S6). The rapid release in SPF suggests that MSNs could be optimized for inhalable AMP therapies, whereas the more gradual release profile observed in SIF makes them suitable for oral drug formulations. Protection against premature degradation in SGF further enhances the bioavailability of encapsulated peptides, a critical factor in peptide‐based therapeutics [108]. These findings suggest that the MSN‐CPP@DC05 has favorable release behavior, with limited peptide release under acidic gastric conditions and more sustained release in intestinal and physiological environments. Although only the nanoplatform MSN‐CPP@DC05 was tested, the observed profile is consistent with previous reports on MSN‐based delivery nanoplatforms and their capacity to protect and gradually release bioactive molecules [109, 110, 111]. Thus, the combined architecture of the silica matrix and its surface functionalization likely play a role in modulating the release kinetics of DC05.

#### 3.6.3 Nanoparticle Uptake and Anti‐*Mtb* Studies

**FIGURE 7:**
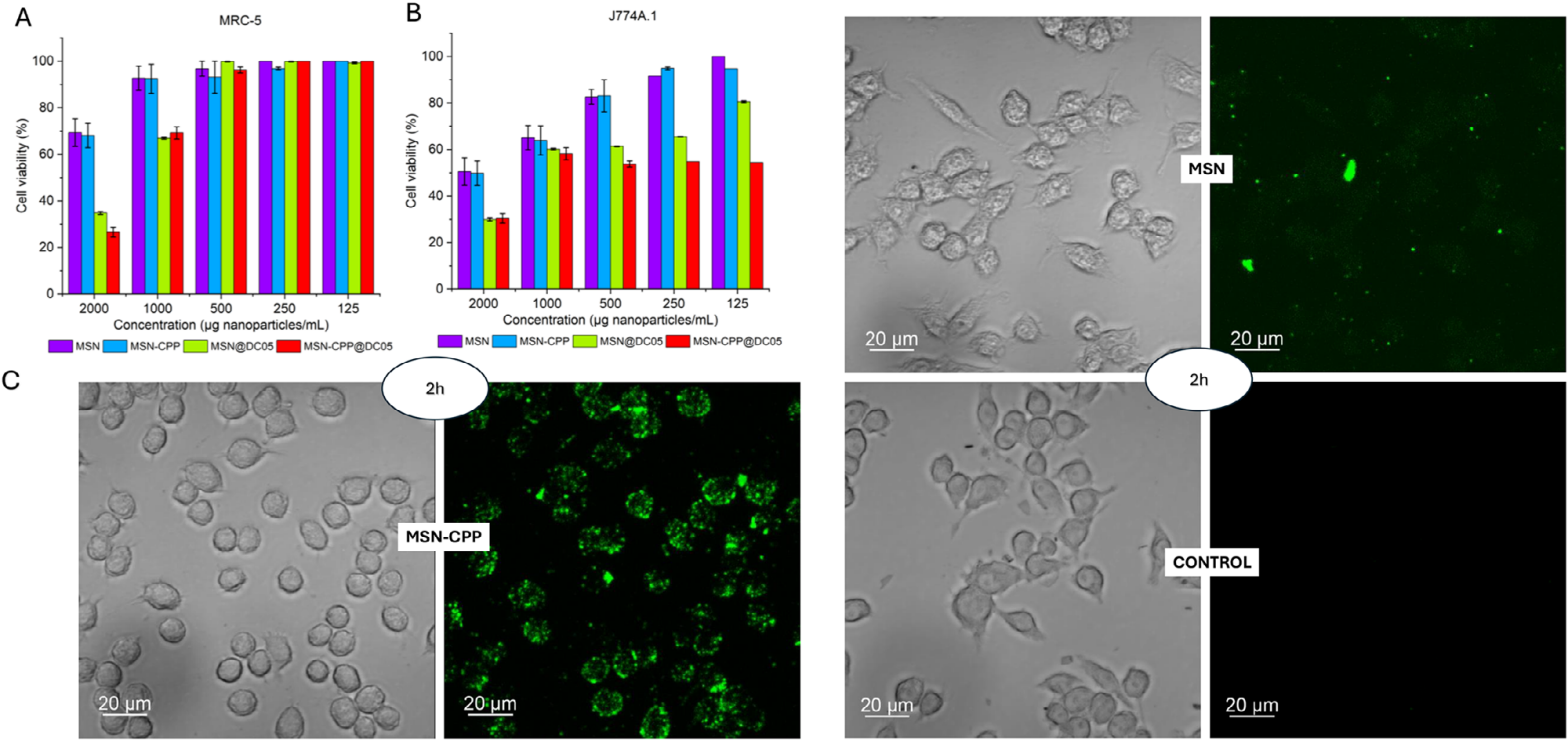
(A) Cell viability of MRC‐5 fibroblasts and (B) J774A.1 macrophages after 24 h of incubation with increasing concentrations (125 to 2000 µg/mL) of MSNs, MSN‐CPP, MSN@DC05, and MSN‐CPP@DC05. (C) Early internalization profile of MSN‐FITC‐CPP and MSN‐FITC in RAW 264.7 macrophages at 2 h. Scale bars = 20 µm (green: nanoparticles).

Cytotoxicity Cytotoxicity was evaluated in human fibroblasts (MRC‐5) and murine macrophages (J774A.1) across nanoparticle concentrations ranging from 125 to 2000 µg/mL (Figure 7A,B). In MRC‐5 cells, all formulations were well tolerated, with viability remaining above 80% even at the highest doses tested. Pristine MSN and MSN‐CPP displayed minimal toxicity, whereas DC05‐loaded systems (MSN@DC05 and MSN‐CPP@DC05) produced only a slight reduction in viability at 2000 µg/mL. In macrophages, a more pronounced concentration‐dependent decrease in viability was observed. Pristine MSN maintained high biocompatibility up to 1000 µg/mL, while CPP conjugation resulted in a modest additional reduction at higher concentrations, consistent with the known membrane activity of cationic CPPs [112]. DC05 loading further decreased macrophage viability, with MSN@DC05 and MSN‐CPP@DC05 showing the lowest values at 2000 µg/mL, while all formulations remained well tolerated at concentrations ≤500 µg/mL. DC05‐loaded systems exhibited greater cytotoxicity in macrophages than in fibroblasts across the tested range. The encapsulation of DC05 within MSN and MSN‐CPP increased macrophage viability compared with the free peptide, which is consistent with a more gradual apparent peptide release in the surrounding medium and reduced nonspecific interactions with cellular components. The higher macrophage tolerance observed for the encapsulated formulations relative to free DC05 is compatible with diminished direct peptide–membrane contact and reduced peak exposure associated with the buffer‐dependent release behavior of the mesoporous matrix [113].

CLSM experiments (Figure 7C) revealed that the CPP enables efficient MSN‐FITC‐CPP uptake by RAW 264.7 macrophages, suggesting a potentially greater benefit in the context of intracellular infections. These findings support the need for intramacrophage infection assays to assess the therapeutic advantage conferred by CPP conjugation more precisely [114]. Notably, in *Mtb*‐related studies, candidates with MIC_90_ values above 25 µg/mL are generally not considered strong therapeutic leads [114, 115]. Thus, the observed MIC_90_ for MSN‐CPP@DC05 indicates that this nanoplatform is a promising candidate for further in vivo validation, reinforcing the advantages of MSN in stabilizing and delivering antimicrobial peptides such as DC05 and highlighting how CPP conjugation enhances cellular uptake and could overcome the barriers posed by the complex *Mtb* cell wall [116, 117].

**FIGURE 8:**
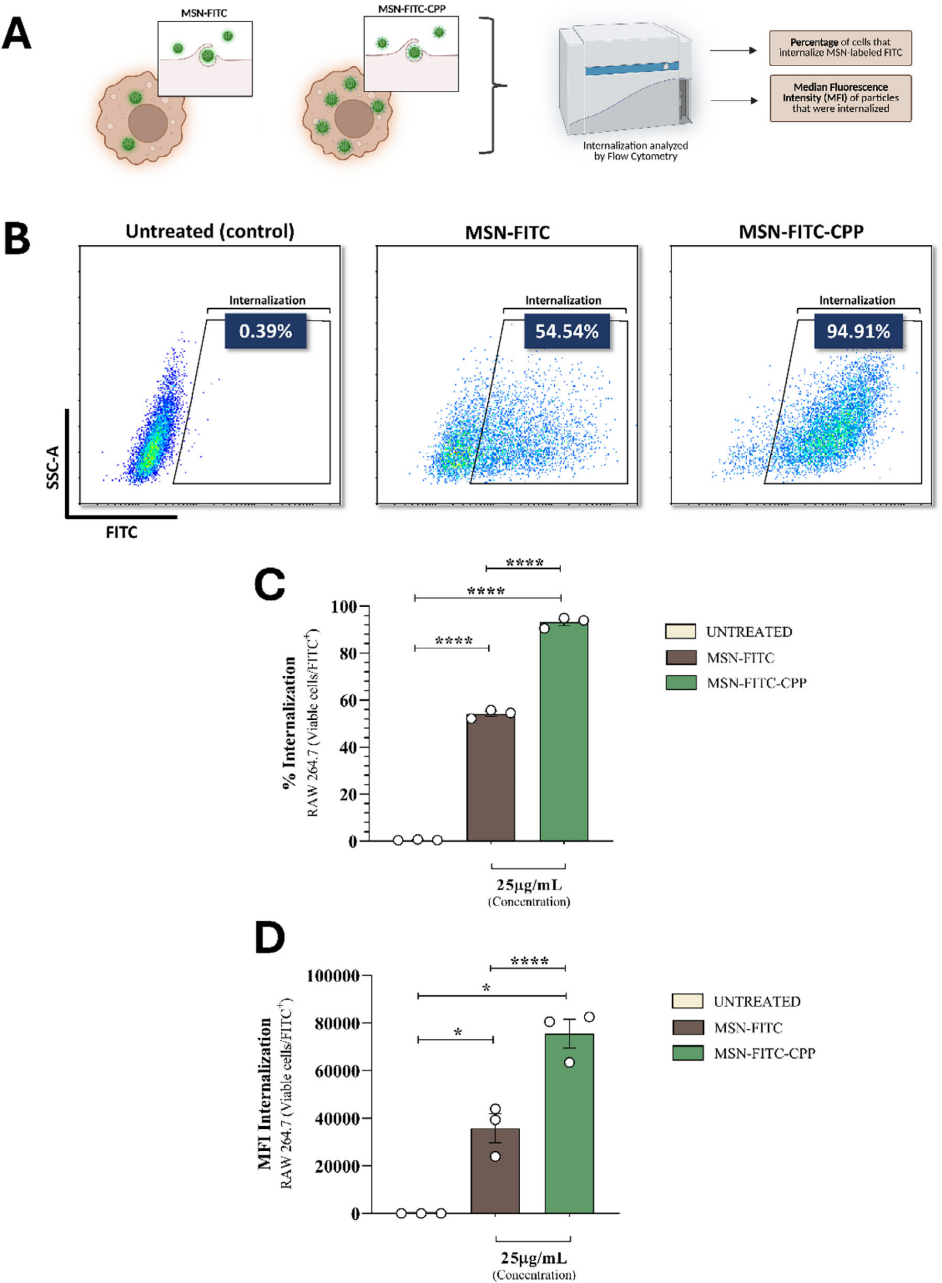
(A) Schematic representation of the flow cytometry assay used to assess nanoparticle uptake. (B) Representative flow cytometry dot plots showing untreated cells (control) and cells exposed to MSN‐FITC or MSN‐FITC‐CPP. (C) Percentage of FITC^+^ cells. (D) MFI of FITC‐labeled MSN in RAW 264.7 cells. Data are expressed as mean ± SEM. Each symbol represents an individual experiment (*n* = 3 biological replicates). ^*^ *p* ≤ 0.0449; ****p < 0.0001. Created with BioRender.com.

To quantitatively complement the qualitative CLSM observations, flow cytometry was performed after 2 h of incubation at 25 µg mL^−1^, confirming the superior cellular uptake of MSN‐FITC‐CPP by macrophages (Figure 8A). Representative dot plots showed a clear increase in fluorescence in the FITC channel following exposure to labeled nanoparticles, with the strongest shift observed for the CPP‐functionalized formulation. The percentage of viable FITC^+^ cells increased from 54.12% ± 1.04% for MSN‐FITC to 93.08 ± 1.35% for MSN‐FITC‐CPP (Figure 8B,C). Likewise, the MFI increased from 35,779 ± 6,070 for MSN‐FITC to 75,505 ± 6,046 for MSN‐FITC‐CPP (Figure 8D). These differences were statistically significant and indicate that CPP functionalization increased both the fraction of macrophages that internalized nanoparticles and the amount of internalized material per cell. This quantitative result is consistent with the trend previously observed by CLSM and agrees with previous reports showing that silica nanoparticle uptake is more robustly characterized by combining microscopy with flow cytometry, while the biological behavior of mesoporous silica nanoparticles depends strongly on surface functionalization [118]. In particular, recent studies have shown that macrophage uptake of MSNs can vary substantially according to surface chemistry and the resulting bio–nano interface [52, 118], whereas CPP‐functionalized MSN systems have been associated with enhanced cellular entry and improved intracellular delivery performance [45, 119]. Within this framework, the ∼1.7‐fold increase in FITC^+^ cells and the ∼2.1‐fold increase in MFI observed here for MSN‐FITC‐CPP relative to MSN‐FITC support the interpretation that CPP conjugation effectively enhances macrophage association/internalization, reinforcing the rationale for using CPP‐decorated MSNs as intracellular carriers in anti‐tuberculosis applications.

**FIGURE 9:**
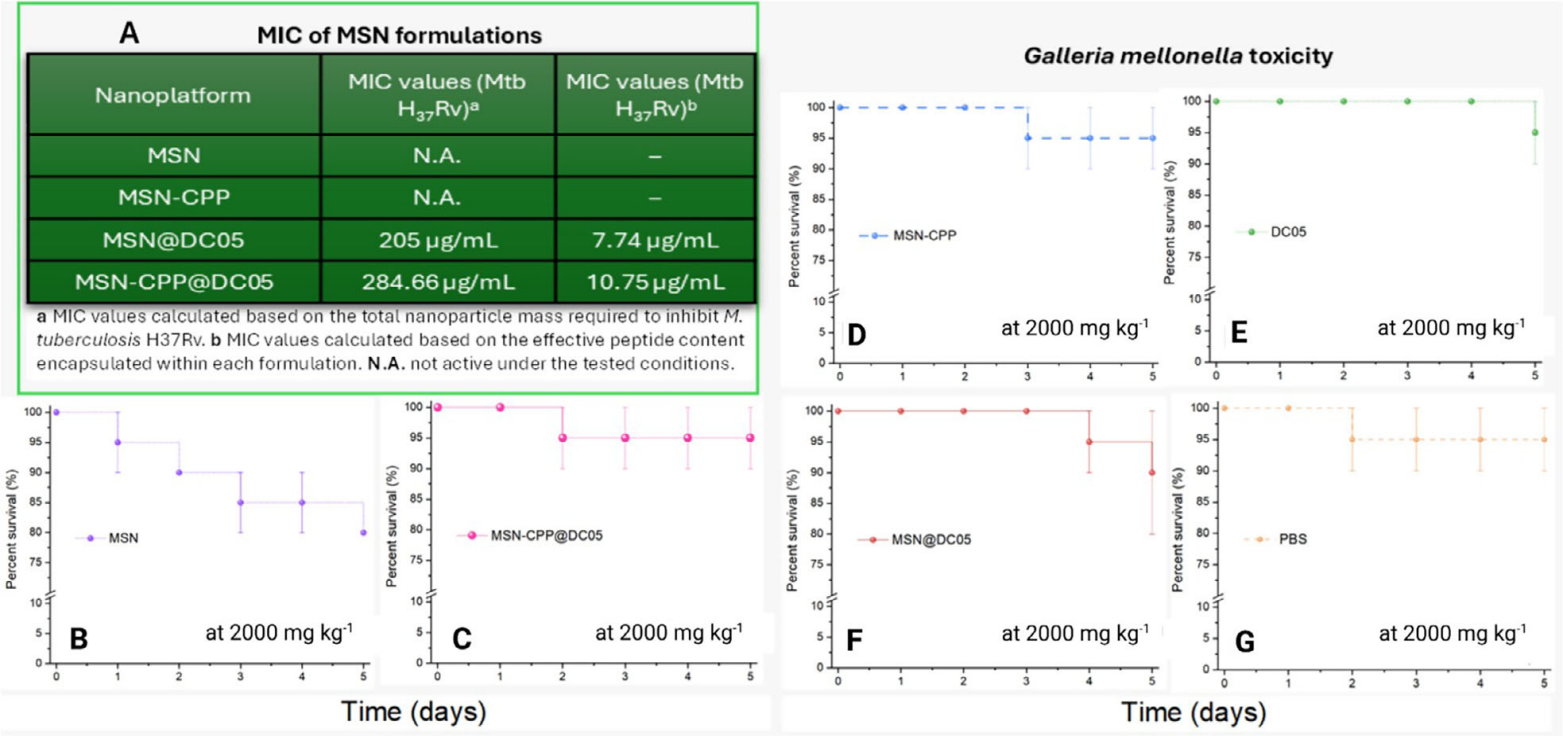
Antimycobacterial activity and in vivo tolerability of MSN‐based nanoplatforms. (A) MIC of MSN, MSN‐CPP, MSN@DC05, and MSN‐CPP@DC05 against Mtb H37Rv. (B–H) Survival curves of *G. mellonella* larvae treated with nanoparticle‐based formulations at 2000 mg kg^−1^ of nanoparticles and with free DC05 at 75.6 mg kg^−1^ (15.1 µg per larva) over 5 days. Each panel corresponds to an independent treatment group: (B) MSN, (C) MSN‐CPP@DC05, (D) MSN‐CPP, (E) free DC05, (F) MSN@DC05, and (H) PBS control. Data represent mean ± SD (*n* = 10 larvae per group).

In the presence of *Mtb* H37Rv (Figure 9A), neither pristine MSNs nor MSN‐CPP were active within the tested range, indicating that antimicrobial activity depended entirely on the DC05 payload. Free DC05 displayed an MIC of 12.46 ± 0.06 µm (23.34 µg/mL). In contrast, the MSN@DC05 formulation required 205 µg/mL of nanoparticles to achieve complete inhibition, corresponding to 7.74 µg/mL (4.13 ± 0.64 µm) of peptide‐equivalent concentration. The CPP‐functionalized formulation (MSN‐CPP@DC05) showed further enhancement, with an MIC of 284.66 µg/mL, equivalent to 10.75 µg/mL (5.74 ± 0.15 µm) of encapsulated DC05. Free CPP remained inactive at the tested concentrations. These data confirm that nanoencapsulation—and, to a greater extent, CPP functionalization—improves the apparent potency relative to free DC05.

Selectivity was assessed using cytotoxicity measurements in MRC‐5 fibroblasts and J774A.1 macrophages (Figure 7). All nanoformulations maintained fibroblast viability above 80% up to 2000 µg/mL, indicating that IC_50_ values were not reached within the tested range. In macrophages, a clearer concentration‐dependent decrease in viability was observed, particularly for DC05‐loaded formulations; however, IC_50_ values could not be determined precisely from the experimental range. Even so, both MSN@DC05 and MSN‐CPP@DC05 remained well tolerated at concentrations ≤500 µg/mL, distinguishing them from free DC05, which showed lower macrophage viability at equivalent peptide doses. CPP coating produced an additional reduction in viability at the highest nanoparticle concentrations, consistent with the known membrane‐interactive properties of cationic CPPs. Overall, the encapsulated formulations preserved a favorable safety profile, and macrophage tolerance was consistently higher for encapsulated DC05 than for the free peptide.

To extend the safety assessment beyond mammalian cell lines, we evaluated acute toxicity in the *G. mellonella* larval model using a dose of 2000 mg kg^−1^ of nanoparticles (corresponding to 75.6 mg kg^−1^ of DC05 in the DC05‐containing groups; Figure 9B–H), a reliable alternative for AMP safety studies, particularly in *Mtb* drug discovery [120]. Survival trajectories for PBS, free DC05, and all nanoplatforms (MSN, MSN‐CPP, MSN@DC05, MSN‐CPP@DC05) were largely superimposable on the vehicle control over five days. By day 5, survival remained high (≥90%–95%) for MSN‐CPP, MSN@DC05, MSN‐CPP@DC05, DC05, and PBS, with a modest reduction for pristine MSN (∼80%–85%), the only group showing divergence. The larvae showed no systemic melanization, loss of the righting reflex, anorexia, or premature pupation, suggesting the absence of stress responses or innate immune overactivation; only mild, localized cuticular darkening was occasionally observed at the injection site [121, 122]. Early deaths, confined to the first 24–48 h, were attributed to handling or injection trauma rather than treatment. Overall, the results of the *G. mellonella* assay support a favorable safety profile for DC05 and its nanoplatforms, which is consistent with the cell‐based data: MSN‐CPP@DC05 did not introduce detectable toxicity, whereas the minor reduction with pristine MSN was nonprogressive. This aligns with the findings of Corrêa et al. [123], who reported that both MSN and lycopene‐loaded MSN are well tolerated in *G. mellonella*, with 93.3% viability even at 2000 mg/kg. Thus, this model provides an orthogonal line of evidence for the biocompatibility of MSN‐CPP@DC05 within antimycobacterial dose ranges.

#### 3.6.4 From Molecular Interactions to Morphological Disruption

**FIGURE 10:**
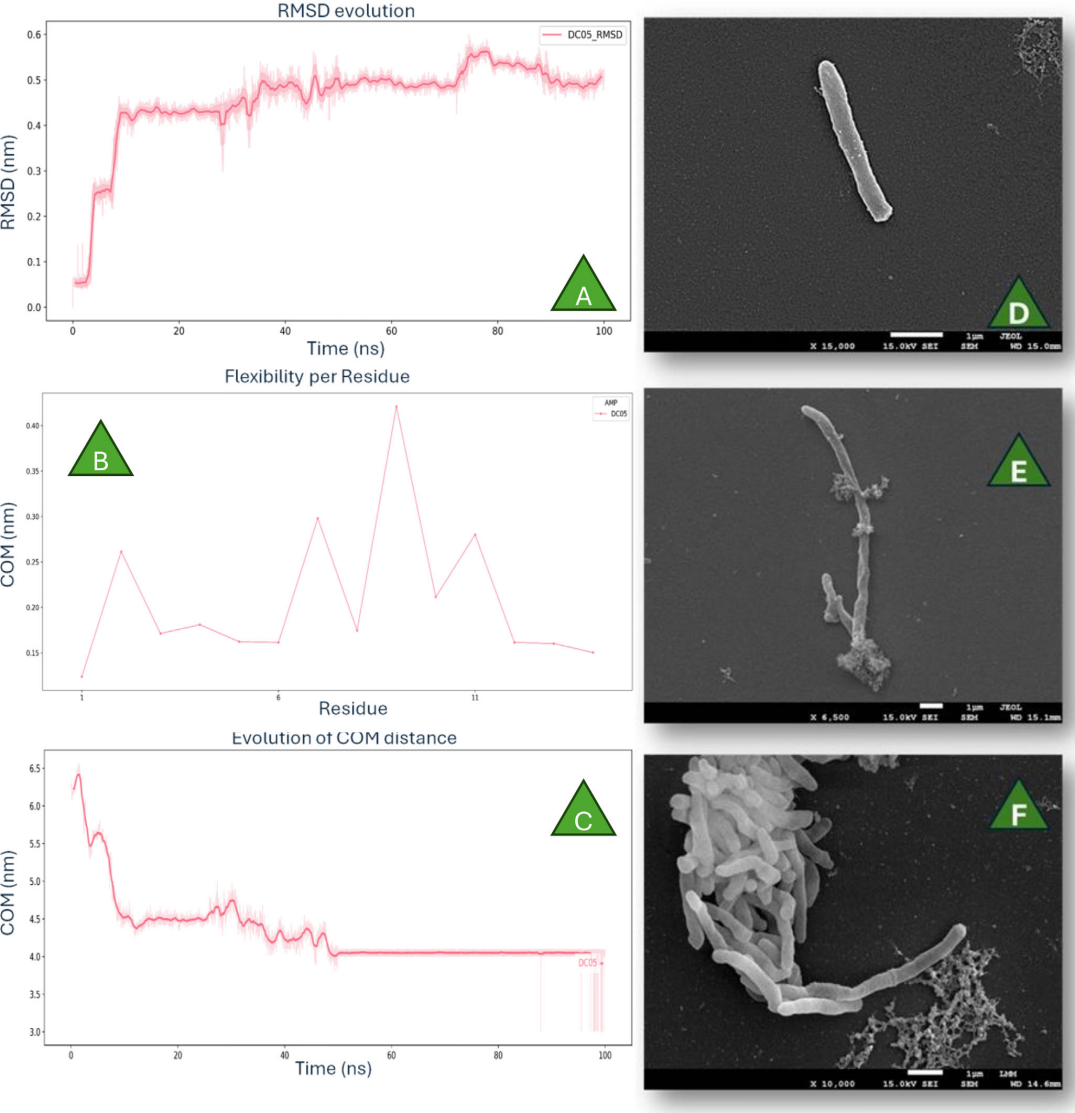
(A) Root‐mean‐square deviation (RMSD) of DC05 during 100 ns of MD simulation. (B) Root‐mean‐square fluctuation (RMSF) analysis per residue. (C) COM distance between DC05 and the membrane over time. (D) SEM image of untreated Mtb. (E) SEM image of Mtb treated with DC05 peptide. (F) SEM image of Mtb treated with MSN‐CPP@DC05.

The antimicrobial mechanism of DC05 against *Mtb* was investigated through a combined approach of MD simulations and SEM, providing complementary insights at both the molecular and cellular levels. The MD simulations were specifically designed to elucidate the initial peptide–membrane interactions that drive the mechanism of action of DC05, offering molecular‐resolution information beyond what can be obtained from morphological assays. The RMSD analysis of DC05 over a 100 ns simulation indicated two distinct conformational transitions before stabilization (Figure 10A). During the initial 5 ns, the RMSD rapidly increased to ∼0.25 nm, suggesting DC05 rearrangement upon contact with the α‐mycolic acid membrane [124, 125]. A second transition phase, from 5 to 10 ns, increased to ∼0.40–0.45 nm, likely reflecting deeper insertion or reorientation within the lipid environment [126, 127]. Beyond 10 ns, the RMSD plateaued between 0.45 and 0.5 nm, indicating stable embedding within the membrane interface, with minor fluctuations attributed to transient local interactions [128]. Importantly, extending the simulation from 50 to 100 ns did not yield additional structural rearrangements, confirming that the system reaches convergence early; thus, the 100‐ns trajectory is shown as representative of the equilibrated state. Residue‐specific flexibility analysis (RMSF, Figure 10B) highlighted Arg7 and Phe9 as regions of increased mobility (>0.29 nm), which is consistent with their roles in membrane engagement [129]. These residues are known to promote peptide‒lipid interactions through electrostatic and hydrophobic forces, as supported by prior studies on membrane‐active peptides [130, 131, 132]. The center of mass (COM) distance profile (Figure 10C) further supported a two‐phase mechanism: initial rapid adsorption, with the distance decreasing from ∼6.5 to ∼4.5 nm in the first 10 ns, followed by a gradual approach to ∼4.0 nm, suggesting sustained association and potential partial insertion into the hydrophobic membrane core [133, 134]. These findings provide a mechanistic rationale for the observed antimicrobial activity, suggesting that DC05 disrupts the integrity of the *Mtb* membrane by targeting its lipid‐rich outer layer, particularly the α‐mycolic acid domains. These simulations were conducted using the free peptide because this configuration represents the biologically relevant state of DC05 after its intracellular release from the MSN‐CPP nanoplatform before contacting the mycolic‐acid membrane of *Mtb*.

The in silico predictions of membrane perturbation were corroborated by SEM analysis of *Mtb* treated with DC05 and its nanoparticle formulation. SEM images of untreated *Mtb* (Figure 10D) revealed intact, elongated bacilli with smooth surfaces, indicative of a healthy, undisturbed cell envelope [63]. In contrast, *Mtb* exposed to DC05 peptide alone (Figure 10E) displayed substantial morphological damage, including collapsed structures, surface irregularities, and evidence of membrane rupture [135]. These alterations are consistent with peptide‐induced membrane destabilization, as predicted by MD simulations. Notably, *Mtb* treated with MSN‐CPP@DC05 exhibited even more pronounced morphological disruptions (Figure 10F), characterized by collapsed cell bodies, irregular contours, and clustered aggregates [136]. These findings suggest that the multifunctional nanoplatform not only retains the membrane‐disruptive capacity of DC05 but also may enhance its local concentration and stability at the bacterial surface, resulting in increased damage. The presence of CPPs on the MSN surface likely facilitated targeted uptake into macrophages, while the sustained release of DC05s from the nanocarrier contributed to prolonged antimicrobial action.

Together, the molecular dynamics simulations and SEM analyses provide a cohesive multiscale model of action, demonstrating that DC05 exerts its antimycobacterial activity by targeting the mycolic acid‐rich membrane of *Mtb*. At the molecular level, the peptide binds and inserts into the lipid bilayer, a process driven by key residues such as Arg7 and Phe9, which facilitate electrostatic and hydrophobic interactions with membrane components [132]. This interaction translates at the cellular level into visible membrane deformation, collapse, and potential lysis, as evidenced by the morphological changes observed via SEM analyses [137]. Moreover, MSN‐CPP@DC05enhances this mechanism by possibly improving peptide stability, targeting, and intracellular accumulation at the infection site, resulting in more pronounced morphological damage [138]. Collectively, these findings support a membrane‐disruptive mechanism as the primary mode of action of DC05 and highlight the synergistic benefits of MSN for optimizing peptide‐based therapeutics against *Mtb* [139].

## 4 Conclusions and Future Perspectives

This study demonstrated that the use of MSN‐CPP@DC05 represents a promising strategy against Mtb. Compared with free DC05, the nanoplatform maintained physicochemical stability, displayed an SBF‐dependent release pattern, improved antimicrobial performance, and preserved a favorable safety margin in both mammalian cells and the *G. mellonella* model. These results reinforce the importance of silica‐based nanocarriers as adaptable nanoplatforms for peptide delivery. Future investigations should evaluate the efficacy of *Mtb* infection in macrophages and develop murine models that better recapitulate TB pathology. Studies on intracellular trafficking, peptide stability under acidic conditions, and long‐term biosafety will provide a clearer view of the therapeutic potential of these materials. In parallel, dedicated experiments will be carried out to formally verify the targeting effect of tuftsin, including comparative uptake in target and non‐target cells, competition with free tuftsin, and in vivo biodistribution after pulmonary administration. Future work will also incorporate quantitative analyses of intracellular uptake to complement the qualitative confocal microscopy observations and refine the understanding of tuftsin‐mediated targeting and internalization. The release profile in simulated body fluid indicates that the current nanosuspension is not suitable for intravenous or oral use, so downstream development will prioritize locally acting pulmonary formulations (such as nano‐in‐micro dry powders or nebulizable nanosuspensions) aimed at maximizing local lung exposure while minimizing systemic circulation, and will include in vivo pharmacokinetic and toxicological studies to quantify systemic exposure, confirm safety margins, and support rational dose selection, including evaluation in combination with current antitubercular drugs. These considerations outline a coherent path for advancing multifunctional silica‐based nanocarriers toward preclinical development in the context of TB therapy.

## Ethics Statement

Hemolysis assays with human blood were conducted with the approval of the Research Ethics Committee of Universidade Estadual Paulista under approval number 4.253.480, associated with the project *“Síntese, caracterização e estudos de diferentes peptídeos bioativos”*.

## Conflicts of Interest

The authors declare no conflicts of interest.

## Supporting information




**Supporting File**: adhm71107‐sup‐0001‐SuppMat.docx.

## Data Availability

The data that support the findings of this study are available from the corresponding author upon reasonable request.

## References

[1] World Health Organization , Global Tuberculosis Report 2024, World Health Organization, Geneva, ISBN: 978‐92‐4‐010153‐1.

[2] F. M. Demarqui , C. S. Carnero Canales , R. T. A. Machado , et al., “[Fe(phen) _3_] ^2+^ and [Fe(phen) _3_] ^2+^ ‐Loaded Nanostructured Lipid System: in Silico, in Vitro, and in Vivo Efficacy against Mycobacterium Tuberculosis,” ACS Omega 10 (2025): 59145–59158, 10.1021/acsomega.5c08350. 41502661 PMC12771237

[3] J. L. Khawbung , D. Nath , and S. Chakraborty , “Drug Resistant Tuberculosis: A Review,” Comparative Immunology, Microbiology and Infectious Diseases 74 (2021): 101574, 10.1016/j.cimid.2020.101574. 33249329

[4] C. S. Carnero Canales , J. I. Marquez Cazorla , R. M. Marquez Cazorla , et al., “Breaking Barriers: the Potential of Nanosystems in Antituberculosis Therapy,” Bioactive Materials 39 (2024): 106–134. 38783925 10.1016/j.bioactmat.2024.05.013 PMC11112550

[5] C. A. Roque‐Borda , Q. Zhang , T. P. T. Nguyen , et al., “Synergistic Combinations of Antimicrobial Peptides and Conventional Antibiotics: A Strategy to Delay Resistance Emergence in World Health Organization Priority Bacteria,” Pharmacological Reviews 78 (2026): 100104. 41389440 10.1016/j.pharmr.2025.100104

[6] C. S. Carnero Canales , J. I. Marquez Cazorla , R. M. Marquez Cazorla , R. M. Sábio , H. A. Santos , and F. R. Pavan , “Combating Gram‐Negative Infections: The Role of Antimicrobial Peptides and Nanotechnology in Overcoming Antibiotic Resistance,” Materials Today Bio 35 (2025): 102381, 10.1016/j.mtbio.2025.102381. PMC12546895 41142413

[7] C. S. Carnero Canales , J. I. Marquez Cazorla , R. M. Marquez Cazorla , et al., “Redefining Therapies for Drug‐Resistant Tuberculosis: Synergistic Effects of Antimicrobial Peptides,” Nanotechnology, and Computational Design Adv Healthc Mater (2026): 03964, 10.1002/adhm.202503964. PMC13068362 41549858

[8] C. A. Roque‐Borda , L. M. D. G. Primo , H. Franzyk , P. R. Hansen , and F. R. Pavan , “Recent Advances in the Development of Antimicrobial Peptides against ESKAPE Pathogens,” Heliyon 10 (2024): e31958, 10.1016/j.heliyon.2024.e31958. 38868046 PMC11167364

[9] C. S. Carnero Canales , C. A. Roque‐Borda , J. I. M. Cazorla , et al., “Forging a New Frontier: Antimicrobial Peptides and Nanotechnology Converging to Conquer Gastrointestinal Pathogens,” Small 21 (2025): 2501431, 10.1002/smll.202501431. 40384187 PMC12232244

[10] W. Weisany , S. Yousefi , S. P. Soufiani , D. Pashang , D. J. McClements , and M. Ghasemlou , “Mesoporous Silica Nanoparticles: a Versatile Platform for Encapsulation and Delivery of Essential Oils for Food Applications,” Advances in Colloid and Interface Science 325 (2024): 103116, 10.1016/j.cis.2024.103116. 38430728

[11] J. Yan , P. Siwakoti , S. Shaw , S. Bose , G. Kokil , and T. Kumeria , “Porous Silicon and Silica Carriers for Delivery of Peptide Therapeutics,” Drug Delivery and Translational Research 14 (2024): 3549–3567, 10.1007/s13346-024-01609-7. 38819767 PMC11499345

[12] R. M. Sábio , A. B. Meneguin , A. Martins dos Santos , A. S. Monteiro , and M. Chorilli , “Exploiting Mesoporous Silica Nanoparticles as Versatile Drug Carriers for Several Routes of Administration,” Microporous and Mesoporous Materials 312 (2021): 110774, 10.1016/j.micromeso.2020.110774.

[13] C. Geibel , J. Theiner , M. Wolter , M. Kramer , W. Lindner , and M. Lämmerhofer , “Controllable Organosilane Monolayer Density of Surface Bonding Using Silatranes for Thiol Functionalization of Silica Particles for Liquid Chromatography and Validation of Microanalytical Method for Elemental Composition Determination,” Journal of Chromatography A 1653 (2021): 462418, 10.1016/j.chroma.2021.462418. 34340056

[14] M. Dowaidar , “Cell‐penetrating Peptides with Nanoparticles Hybrid Delivery Vectors and Their Uptake Pathways,” Mitochondrion 78 (2024): 101906, 10.1016/j.mito.2024.101906. 38797356

[15] F. Wan , F. Wong , J. J. Collins , and C. de la Fuente‐Nunez , “Machine Learning for Antimicrobial Peptide Identification and Design,” Nature Reviews Bioengineering 2 (2024): 392–407, 10.1038/s44222-024-00152-x. PMC11756916 39850516

[16] C. de la Fuente‐Nunez , “AI in Infectious Diseases: the Role of Datasets,” Drug Resistance Updates 73 (2024): 101067, 10.1016/j.drup.2024.101067. 38387282 PMC11537278

[17] C. A. Brizuela , G. Liu , J. M. Stokes , and C. de la Fuente‐Nunez , “AI Methods for Antimicrobial Peptides: Progress and Challenges,” Microbial Biotechnology 18 (2025): 70072, 10.1111/1751-7915.70072. PMC11702388 39754551

[18] B. Manavalan , S. Basith , T. H. Shin , L. Wei , and G. Lee , “AtbPpred: a Robust Sequence‐Based Prediction of Anti‐Tubercular Peptides Using Extremely Randomized Trees,” Computational and Structural Biotechnology Journal 17 (2019): 972–981, 10.1016/j.csbj.2019.06.024. 31372196 PMC6658830

[19] E. Tenland , N. Krishnan , A. Rönnholm , et al., “A Novel Derivative of the Fungal Antimicrobial Peptide Plectasin is Active against Mycobacterium Tuberculosis,” Tuberculosis 113 (2018): 231–238, 10.1016/j.tube.2018.10.008. 30514507 PMC6289163

[20] S. Inoue , S. Yoshimoto , and K. Hori , “A New Target of Multiple Lysine Methylation in Bacteria,” Journal of Bacteriology 207 (2025): 1, 10.1128/jb.00325-24. PMC11784438 39660925

[21] D. Purcell , M. A. Packer , and M. Hayes , “Identification of Bioactive Peptides from a Laminaria Digitata Protein Hydrolysate Using in Silico and in Vitro Methods to Identify Angiotensin‐1‐Converting Enzyme (ACE‐1) Inhibitory Peptides,” Marine Drugs 21 (2023): 90, 10.3390/md21020090. 36827131 PMC9967564

[22] D. E. V. Pires , T. L. Blundell , and D. B. Ascher , “pkCSM: Predicting Small‐Molecule Pharmacokinetic and Toxicity Properties Using Graph‐Based Signatures,” Journal of Medicinal Chemistry 58 (2015): 4066–4072, 10.1021/acs.jmedchem.5b00104. 25860834 PMC4434528

[23] E. Gasteiger , C. Hoogland , and A. Gattiker , The Proteomics Protocols Handbook (Humana Press, 2005), 571–607, 10.1385/1592598900.

[24] EMBL‐EBI , pLDDT: Understanding local confidence (2023) (available at https://www.ebi.ac.uk/training/online/courses/alphafold/inputs‐and‐outputs/evaluating‐alphafolds‐predicted‐structures‐using‐confidence‐scores/plddt‐understanding‐local‐confidence/).

[25] J. Abramson , J. Adler , J. Dunger , et al., “Accurate Structure Prediction of Biomolecular Interactions With AlphaFold 3,” Nature 630 (2024): 493–500. 38718835 10.1038/s41586-024-07487-w PMC11168924

[26] J. Yang and Y. Zhang , “I‐TASSER Server: New Development for Protein Structure and Function Predictions,” Nucleic Acids Research 43 (2015): W174–W181, 10.1093/nar/gkv342. 25883148 PMC4489253

[27] R. Bansal , S. Mohagaonkar , A. Sen , U. Khanam , and B. Rathi , “In‐Silico Study of Peptide‐protein Interaction of Antimicrobial Peptides Potentially Targeting SARS and SARS‐CoV‐2 Nucleocapsid Protein,” In Silico Pharmacology 9 (2021): 46. 34336545 10.1007/s40203-021-00103-z PMC8315091

[28] A. Irum , R. Abdul , A. Haroon , et al., “A Computational Structural Analysis of Functional Attributes of Hypodermin A and B Proteins: A Way Forward for Vaccine Development—PubMed,” Pak J Pharm Sci 31 (2018): 2443–2451. 30473516

[29] L. M. D. G. Primo , C. A. Roque‐Borda , C. S. Carnero Canales , et al., “Antimicrobial Peptides Grafted onto the Surface of N‐acetylcysteine‐chitosan Nanoparticles Can Revitalize Drugs against Clinical Isolates of Mycobacterium Tuberculosis,” Carbohydrate Polymers 323 (2024): 121449, 10.1016/j.carbpol.2023.121449. 37940311

[30] A. Kotynia , A. Marciniak , W. Kamysz , D. Neubauer , and E. Krzyżak , “Interaction of Positively Charged Oligopeptides with Blood Plasma Proteins,” International Journal of Molecular Sciences 24 (2023): 2836, 10.3390/ijms24032836. 36769160 PMC9918186

[31] C. A. Roque‐Borda , O. J. Ramirez Delgado , L. M. Duran Gleriani Primo , et al., “Integrating Docking, Dynamics, and Assays to Predict Antimicrobial Peptide Interactions with Mycolic Acid Membranes in Mycobacterium Tuberculosis,” ACS Measurement Science Au 5 (2025): 981–1000, 10.1021/acsmeasuresciau.5c00126. 41425329 PMC12715740

[32] R. B. Merrifield , “Solid Phase Peptide Synthesis. I. The Synthesis of a Tetrapeptide,” Journal of the American Chemical Society 85 (1963): 2149–2154, 10.1021/ja00897a025.

[33] J. C. Palomino , A. Martin , M. Camacho , H. Guerra , J. Swings , and F. Portaels , “Resazurin Microtiter Assay Plate: Simple and Inexpensive Method for Detection of Drug Resistance in Mycobacterium Tuberculosis,” Antimicrobial Agents and Chemotherapy 46 (2002): 2720–2722, 10.1128/AAC.46.8.2720-2722.2002. 12121966 PMC127336

[34] A. P. B. Silva , C. A. Roque‐Borda , C. S. Carnero Canales , et al., “Activity of Bacteriophage D29 Loaded on Nanoliposomes Against Macrophages Infected with Mycobacterium Tuberculosis,” Diseases 11 (2023): 150. 37987261 10.3390/diseases11040150 PMC10660732

[35] K. R. Caleffi‐Ferracioli , R. C. R. Amaral , F. O. Demitto , et al., “Morphological Changes and Differentially Expressed Efflux Pump Genes in Mycobacterium Tuberculosis Exposed to a Rifampicin and Verapamil Combination,” Tuberculosis 97 (2016): 65–72, 10.1016/j.tube.2015.12.010. 26980498

[36] D. R. Serrano , L. Hernández , L. Fleire , et al., “Hemolytic and Pharmacokinetic Studies of Liposomal and Particulate Amphotericin B Formulations,” International Journal of Pharmaceutics 447 (2013): 38–46, 10.1016/j.ijpharm.2013.02.038. 23438978

[37] D. M. Maron and B. N. Ames , “Revised Methods for the Salmonella Mutagenicity Test,” Mutation Research/Environmental Mutagenesis and Related Subjects 113 (1983): 173–215, 10.1016/0165-1161(83)90010-9. 6341825

[38] R. M. Sábio , S. H. Santagneli , M. Gressier , et al., “Near‐infrared/Visible‐emitting Nanosilica Modified with Silylated Ru(II) and Ln(III) Complexes,” Nanotechnology 31 (2020): 035602, 10.1088/1361-6528/ab494f. 31569083

[39] R. M. Sábio , S. H. Santagneli , M. Gressier , et al., “Luminescent Nanohybrids Based on Silica and Silylated Ru(II)—Yb(III) Heterobinuclear Complex: New Tools for Biological media Analysis,” Nanotechnology 31 (2020): 085709, 10.1088/1361-6528/ab55c3. 31703226

[40] Y. Wang , H.‐Y. Huang , L. Yang , Z. Zhang , and H. Ji , “Cetuximab‐modified Mesoporous Silica Nano‐medicine Specifically Targets EGFR‐mutant Lung Cancer and Overcomes Drug Resistance,” Scientific Reports 6 (2016): 25468, 10.1038/srep25468. 27151505 PMC4858690

[41] C.‐A. Cheng , W. Chen , L. Zhang , H. H. Wu , and J. I. Zink , “A Responsive Mesoporous Silica Nanoparticle Platform for Magnetic Resonance Imaging‐Guided High‐Intensity Focused Ultrasound‐Stimulated Cargo Delivery with Controllable Location, Time, and Dose,” Journal of the American Chemical Society 141 (2019): 17670–17684, 10.1021/jacs.9b07591. 31604010

[42] L. Mauline , M. Gressier , C. Roques , et al., “Bifunctional Silica Nanoparticles for the Exploration of Biofilms of Pseudomonas aeruginosa,” Biofouling 29 (2013): 775–788, 10.1080/08927014.2013.798866. 23805884

[43] E. Tenland , A. Pochert , N. Krishnan , et al., “Effective Delivery of the Anti‐mycobacterial Peptide NZX in Mesoporous Silica Nanoparticles,” PLoS ONE 14 (2019): 0212858, 10.1371/journal.pone.0212858. PMC6391042 30807612

[44] J. P. Martins , P. Figueiredo , S. Wang , et al., “Neonatal Fc Receptor‐targeted Lignin‐encapsulated Porous Silicon Nanoparticles for Enhanced Cellular Interactions and Insulin Permeation across the Intestinal Epithelium,” Bioact Mater 9 (2022): 299–315. 34820572 10.1016/j.bioactmat.2021.08.007 PMC8586719

[45] N. Shadmani , P. Makvandi , M. Parsa , et al., “Enhancing Methotrexate Delivery in the Brain by Mesoporous Silica Nanoparticles Functionalized with Cell‐Penetrating Peptide Using in Vivo and Ex Vivo Monitoring,” Molecular Pharmaceutics 20 (2023): 1531–1548, 10.1021/acs.molpharmaceut.2c00755. 36763486

[46] G. Quan , X. Pan , Z. Wang , et al., “Lactosaminated Mesoporous Silica Nanoparticles for Asialoglycoprotein Receptor Targeted Anticancer Drug Delivery,” Journal of Nanobiotechnology 13 (2015): 7, 10.1186/s12951-015-0068-6. 25643602 PMC4333889

[47] L. Jia , J. Shen , Z. Li , et al., “Successfully Tailoring the Pore Size of Mesoporous Silica Nanoparticles: Exploitation of Delivery Systems for Poorly Water‐soluble Drugs,” International Journal of Pharmaceutics 439 (2012): 81–91, 10.1016/j.ijpharm.2012.10.011. 23078857

[48] D. Liu , J. Li , H. Pan , et al., “Potential Advantages of a Novel Chitosan‐N‐acetylcysteine Surface Modified Nanostructured Lipid Carrier on the Performance of Ophthalmic Delivery of Curcumin,” Scientific Reports 6 (2016): 1–14. 27350323 10.1038/srep28796 PMC4923878

[49] J. Li , D. Liu , G. Tan , Z. Zhao , X. Yang , and W. Pan , “A Comparative Study on the Efficiency of Chitosan‐N‐acetylcysteine, Chitosan Oligosaccharides or Carboxymethyl Chitosan Surface Modified Nanostructured Lipid Carrier for Ophthalmic Delivery of Curcumin,” Carbohydrate Polymers 146 (2016): 435–444, 10.1016/j.carbpol.2016.03.079. 27112894

[50] J. M. Galdopórpora , C. Martinena , E. Bernabeu , et al., “Inhalable Mannosylated Rifampicin–Curcumin Co‐Loaded Nanomicelles with Enhanced in Vitro Antimicrobial Efficacy for an Optimized Pulmonary Tuberculosis Therapy,” Pharmaceutics 14 (2022): 959, 10.3390/pharmaceutics14050959. 35631546 PMC9145552

[51] C. A. Roque‐Borda , M. M. S. de Saraiva , W. D. Macedo Junior , et al., “Chitosan and HPMCAS Double‐Coating as Protective Systems for Alginate Microparticles Loaded with Ctx(Ile21)‐Ha Antimicrobial Peptide to Prevent Intestinal Infections,” Biomaterials 293 (2023): 121978, 10.1016/j.biomaterials.2022.121978. 36580719

[52] J. Wen , C. Lei , S. Hua , et al., “Regulation of Macrophage Uptake through the Bio‐nano Interaction Using Surface Functionalized Mesoporous Silica Nanoparticles with Large Radial Pores,” Journal of Materials Chemistry B 13 (2025): 137–150, 10.1039/D4TB01124D. 39575665

[53] L. S. Derengowski , C. De‐Souza‐Silva , S. V. Braz , et al., “Antimicrobial Effect of Farnesol, a Candida albicans Quorum Sensing Molecule, on Paracoccidioides Brasiliensis Growth and Morphogenesis,” Annals of Clinical Microbiology and Antimicrobials 8 (2009): 13, 10.1186/1476-0711-8-13. 19402910 PMC2681445

[54] E. Allegra , R. W. Titball , J. Carter , and O. L. Champion , “Galleria Mellonella Larvae Allow the Discrimination of Toxic and Non‐toxic Chemicals,” Chemosphere 198 (2018): 469–472, 10.1016/j.chemosphere.2018.01.175. 29425947

[55] K. D. Saint Jean , K. D. Henderson , C. L. Chrom , L. E. Abiuso , L. M. Renn , and G. A. Caputo , “Effects of Hydrophobic Amino Acid Substitutions on Antimicrobial Peptide Behavior,” Probiotics and Antimicrobial Proteins 10 (2018): 408–419, 10.1007/s12602-017-9345-z. 29103131

[56] K. J. Cutrona , B. A. Kaufman , D. M. Figueroa , and D. E. Elmore , “Role of Arginine and Lysine in the Antimicrobial Mechanism of Histone‐Derived Antimicrobial Peptides,” FEBS Letters 589 (2015): 3915–3920, 10.1016/j.febslet.2015.11.002. 26555191 PMC4713009

[57] A. Aliashkevich and F. Cava , “LD‐Transpeptidases: the Great Unknown among the Peptidoglycan Cross‐Linkers,” The FEBS Journal 289 (2022): 4718–4730, 10.1111/febs.16066. 34109739

[58] A. C. Spencer and S. S. Panda , “DNA Gyrase as a Target for Quinolones,” Biomedicines 11 (2023): 371, 10.3390/biomedicines11020371. 36830908 PMC9953508

[59] S. Maher and D. J. Brayden , “Overcoming Poor Permeability: Translating Permeation Enhancers for Oral Peptide Delivery,” Drug Discovery Today: Technologies 9 (2012): e113–e119, 10.1016/j.ddtec.2011.11.006. 24064271

[60] J. H. Lin and M. Yamazaki , “Role of P‐Glycoprotein in Pharmacokinetics,” Clinical Pharmacokinetics 42 (2003): 59–98, 10.2165/00003088-200342010-00003. 12489979

[61] R. Watanabe , T. Esaki , H. Kawashima , et al., “Predicting Fraction Unbound in Human Plasma from Chemical Structure: Improved Accuracy in the Low Value Ranges,” Molecular Pharmaceutics 15 (2018): 5302–5311, 10.1021/acs.molpharmaceut.8b00785. 30259749

[62] A. M. Ginsberg , “Drugs in Development for Tuberculosis,” Drugs 70 (2010): 2201–2214, 10.2165/11538170-000000000-00000. 21080738

[63] D. L. Campos , C. S. C. Canales , F. M. Demarqui , et al., “Screening of Novel Narrow‐Spectrum Benzofuroxan Derivatives for the Treatment of Multidrug‐Resistant Tuberculosis through in Silico, In Vitro, and In Vivo Approaches,” Frontiers in Microbiology 15 (2024): 1487829, 10.3389/fmicb.2024.1487829. 39464394 PMC11502347

[64] I. L. Elisha , F. S. Botha , B. Madikizela , L. J. McGaw , and J. N. Eloff , “Acetone Leaf Extracts of some South African Trees with High Activity against Escherichia coli Also Have Good Antimycobacterial Activity and Selectivity Index,” BMC Complementary and Alternative Medicine 17 (2017): 327, 10.1186/s12906-017-1831-z. 28629354 PMC5477271

[65] G. Degiacomi , J. C. Sammartino , V. Sinigiani , P. Marra , A. Urbani , and M. R. Pasca , “In Vitro Study of Bedaquiline Resistance in Mycobacterium Tuberculosis Multi‐Drug Resistant Clinical Isolates,” Frontiers in Microbiology 11 (2020): 559469, 10.3389/fmicb.2020.559469. 33042066 PMC7527418

[66] K. Sikora , M. Jaśkiewicz , D. Neubauer , et al., “Counter‐ion Effect on Antistaphylococcal Activity and Cytotoxicity of Selected Antimicrobial Peptides,” Amino Acids 50 (2018): 609–619, 10.1007/s00726-017-2536-9. 29307075 PMC5917001

[67] J. Read and S. Brenner , Encyclopedia of Genetics (Elsevier, 2001), 1189.

[68] X. Ye , H. Zhang , X. Luo , et al., “Characterization of the Hemolytic Activity of Mastoparan Family Peptides from Wasp Venoms,” Toxins 15 (2023): 591, 10.3390/toxins15100591. 37888622 PMC10611374

[69] M. Lata , V. Telang , P. Gupta , et al., “Evolutionary and in Silico Guided Development of Novel Peptide Analogues for Antibacterial Activity against ESKAPE Pathogens,” Current Research in Microbial Sciences 4 (2023): 100183, 10.1016/j.crmicr.2023.100183. 37032813 PMC10073642

[70] P. V. Dubovskii and R. G. Efremov , “The Role of Hydrophobic /Hydrophilic Balance in the Activity of Structurally Flexible vs. rigid Cytolytic Polypeptides and Analogs Developed on Their Basis,” Expert Review of Proteomics 15 (2018): 873–886, 10.1080/14789450.2018.1537786. 30328726

[71] K. Jin , “Developing Cyclic Peptide‐based Drug Candidates: an Overview,” Future Medicinal Chemistry 12 (2020): 1687–1690, 10.4155/fmc-2020-0171. 32972246

[72] A. Gaurav , P. Bakht , M. Saini , S. Pandey , and R. Pathania , “Role of Bacterial Efflux Pumps in Antibiotic Resistance, Virulence, and Strategies to Discover Novel Efflux Pump Inhibitors,” Microbiology 169 (2023): 001333, 10.1099/mic.0.001333. 37224055 PMC10268834

[73] P. A. Klenotic , M. A. Moseng , C. E. Morgan , and E. W. Yu , “Structural and Functional Diversity of Resistance–Nodulation–Cell Division Transporters,” Chemical Reviews 121 (2021): 5378–5416, 10.1021/acs.chemrev.0c00621. 33211490 PMC8119314

[74] M. Laws , P. Jin , and K. M. Rahman , “Efflux Pumps in Mycobacterium Tuberculosis and Their Inhibition to Tackle Antimicrobial Resistance,” Trends in Microbiology 30 (2022): 57–68, 10.1016/j.tim.2021.05.001. 34052094

[75] L. Rodrigues , J. Ramos , I. Couto , L. Amaral , and M. Viveiros , “Ethidium Bromide Transport across Mycobacterium Smegmatiscell‐wall: Correlation with Antibiotic Resistance,” BMC Microbiology 11 (2011): 35, 10.1186/1471-2180-11-35. 21332993 PMC3051877

[76] M. T. Botelho , G. G. Militão , M. Brinkmann , and G. D. A. Umbuzeiro , “Toxicity and Mutagenicity Studies of <Scp>6PPD</Scp>‐quinone in a Marine Invertebrate Species and Bacteria,” Environmental and Molecular Mutagenesis 64 (2023): 335–341. 37402651 10.1002/em.22560

[77] H. Bugda , B. Guven Ezer , and E. Rencuzogullari , “In Vitro Screening of Genotoxicity and Mutagenicity of Pyriproxyfen in human Lymphocytes and Salmonella Typhimurium TA98 and TA100 Strains,” Drug and Chemical Toxicology 46 (2023): 955–961, 10.1080/01480545.2022.2113096. 35982527

[78] K. T. Angula , L. J. Legoabe , and R. M. Beteck , “Chemical Classes Presenting Novel Antituberculosis Agents Currently in Different Phases of Drug Development: a 2010–2020 Review,” Pharmaceuticals 14 (2021): 461, 10.3390/ph14050461. 34068171 PMC8152995

[79] E. Cordelli , M. Bignami , and F. Pacchierotti , “Comet Assay: a Versatile but Complex Tool in Genotoxicity Testing,” Toxicology Research 10 (2021): 68–78, 10.1093/toxres/tfaa093. 33613974 PMC7885189

[80] R. Abbasi , G. Shineh , M. Mobaraki , S. Doughty , and L. Tayebi , “Structural Parameters of Nanoparticles Affecting Their Toxicity for Biomedical Applications: a Review,” Journal of Nanoparticle Research 25 (2023): 43, 10.1007/s11051-023-05690-w. 36875184 PMC9970140

[81] T. D. C. Ribeiro , R. M. Sábio , M. T. Luiz , et al., “Curcumin‐Loaded Mesoporous Silica Nanoparticles Dispersed in Thermo‐Responsive Hydrogel as Potential Alzheimer Disease Therapy,” Pharmaceutics 14 (2022): 1976, 10.3390/pharmaceutics14091976. 36145723 PMC9504573

[82] M. Haripriyaa and K. Suthindhiran , “Pharmacokinetics of Nanoparticles: Current Knowledge, Future Directions and Its Implications in Drug Delivery,” Future Journal of Pharmaceutical Sciences 9 (2023): 113, 10.1186/s43094-023-00569-y.

[83] N. Hoshyar , S. Gray , H. Han , and G. Bao , “The Effect of Nanoparticle Size on in Vivo Pharmacokinetics and Cellular Interaction,” Nanomedicine 11 (2016): 673–692, 10.2217/nnm.16.5. 27003448 PMC5561790

[84] S. A. A. Rizvi and A. M. Saleh , “Applications of Nanoparticle Systems in Drug Delivery Technology,” Saudi Pharmaceutical Journal 26 (2018): 64–70, 10.1016/j.jsps.2017.10.012. 29379334 PMC5783816

[85] S. Lungare , K. Hallam , and R. K. S. Badhan , “Phytochemical‐loaded Mesoporous Silica Nanoparticles for Nose‐to‐brain Olfactory Drug Delivery,” International Journal of Pharmaceutics 513 (2016): 280–293, 10.1016/j.ijpharm.2016.09.042. 27633279

[86] N. S. Elbialy , S. F. Aboushoushah , B. F. Sofi , and A. Noorwali , “Multifunctional Curcumin‐loaded Mesoporous Silica Nanoparticles for Cancer Chemoprevention and Therapy,” Microporous and Mesoporous Materials 291 (2020): 109540, 10.1016/j.micromeso.2019.06.002.

[87] B. Beitzinger , R. Schmid , C. Jung , et al., “Confinement and Polarity Effects on the Peptide Packing Density on Mesoporous Silica Nanoparticles,” Langmuir 40 (2024): 4294–4305, 10.1021/acs.langmuir.3c03513. 38346113 PMC10905996

[88] N. Boehnke , K. J. Dolph , V. M. Juarez , J. M. Lanoha , and P. T. Hammond , “Electrostatic Conjugation of Nanoparticle Surfaces with Functional Peptide Motifs,” Bioconjugate Chemistry 31 (2020): 2211–2219, 10.1021/acs.bioconjchem.0c00384. 32786506 PMC7895459

[89] A. A. Abd‐Elrahman , M. A. El Nabarawi , D. H. Hassan , and A. A. Taha , “Ketoprofen Mesoporous Silica Nanoparticles SBA‐15 Hard Gelatin Capsules: Preparation and in Vitro /in Vivo Characterization,” Drug Delivery 23 (2016): 3387–3398, 10.1080/10717544.2016.1186251. 27167529

[90] S. Anas , R. Metz , M. A. Sanoj , R. V. Mangalaraja , and S. Ananthakumar , “Sintering of Surfactant Modified ZnO–Bi2O_3_ Based Varistor Nanopowders,” Ceramics International 36 (2010): 2351–2358, 10.1016/j.ceramint.2010.07.017.

[91] A. Ulu , S. A. A. Noma , S. Koytepe , and B. Ates , “Magnetic Fe_3_ O_4_ @MCM‐41 Core–shell Nanoparticles Functionalized with Thiol Silane for Efficient <Scp>L</Scp>‐asparaginase Immobilization,” Artif Cells Nanomed Biotechnol 46 (2018): 1035–1045. 29873527 10.1080/21691401.2018.1478422

[92] M. A. Khan , “Targeted Drug Delivery Using Tuftsin‐bearing Liposomes: Implications in the Treatment of Infectious Diseases and Tumors,” Current Drug Targets 22 (2021): 770–778. 33243117 10.2174/1389450121999201125200756

[93] J. C. Nissen , D. L. Selwood , and S. E. Tsirka , “Tuftsin Signals through Its Receptor Neuropilin‐1 via the Transforming Growth Factor Beta Pathway,” Journal of Neurochemistry 127 (2013): 394–402, 10.1111/jnc.12404. 24033337 PMC3805743

[94] P. Bala , B. K. Samantaray , and S. K. Srivastava , “Dehydration Transformation in Ca‐montmorillonite,” Bulletin of Materials Science 23 (2000): 61–67, 10.1007/BF02708614.

[95] D. Awotwe‐Otoo , C. Agarabi , D. Keire , et al., “Physicochemical Characterization of Complex Drug Substances: Evaluation of Structural Similarities and Differences of Protamine Sulfate from Various Sources,” The AAPS Journal 14 (2012): 619–626, 10.1208/s12248-012-9375-0. 22678712 PMC3385841

[96] R. Narayan , U. Y. Nayak , A. M. Raichur , and S. Garg , “Mesoporous Silica Nanoparticles: a Comprehensive Review on Synthesis and Recent Advances,” Pharmaceutics 10 (2018): 118, 10.3390/pharmaceutics10030118. 30082647 PMC6160987

[97] F. Basile , S. Zhang , S. K. Kandar , and L. Lu , “Mass Spectrometry Characterization of the Thermal Decomposition/Digestion (TDD) at Cysteine in Peptides and Proteins in the Condensed Phase,” Journal of the American Society for Mass Spectrometry 22 (2011): s13361–011–0222–0229, 10.1007/s13361-011-0222-9. PMC3195377 21952765

[98] W. Xue‐ying , W. Ya‐zhen , D. Yu‐tao , L. Tian‐yu , and Z. Li‐wu , “Preparation and Thermal Decomposition Kinetics of Novel Silane Coupling Agent with Mercapto Group,” Journal of Nanomaterials 2019 (2019): 1–9, 10.1155/2019/6089065.

[99] Y. H. Tengjisi , G. Yang , C. Fu , Y. Liu , and C.‐X. Zhao , “Biomimetic Core–Shell Silica Nanoparticles Using a Dual‐functional Peptide,” Journal of Colloid and Interface Science 581 (2021): 185–194, 10.1016/j.jcis.2020.07.107. 32771730

[100] B. D. Mather , K. Viswanathan , K. M. Miller , and T. E. Long , “Michael Addition Reactions in Macromolecular Design for Emerging Technologies,” Progress in Polymer Science 31 (2006): 487–531, 10.1016/j.progpolymsci.2006.03.001.

[101] J. Hu , D. Xiao , and X. Zhang , “Advances in Peptide Functionalization on Mesoporous Silica Nanoparticles for Controlled Drug Release,” Small 12 (2016): 3344–3359, 10.1002/smll.201600325. 27152737

[102] T. M. Albayati , S. M. Alardhi , A. H. Khalbas , et al., “Comprehensive Review of Mesoporous Silica Nanoparticles: Drug Loading, Release, and Applications as Hemostatic Agents,” ChemistrySelect 9 (2024): 202400450, 10.1002/slct.202400450.

[103] S. L. Perry and D. J. McClements , “Recent Advances in Encapsulation, Protection, and Oral Delivery of Bioactive Proteins and Peptides Using Colloidal Systems,” Molecules (Basel, Switzerland) 25 (2020): 1161, 10.3390/molecules25051161. 32150848 PMC7179163

[104] C. D. Spicer , C. Jumeaux , B. Gupta , and M. M. Stevens , “Peptide and Protein Nanoparticle Conjugates: Versatile Platforms for Biomedical Applications,” Chemical Society Reviews 47 (2018): 3574–3620, 10.1039/C7CS00877E. 29479622 PMC6386136

[105] H. A. Rothan , Z. Mohamed , A. M. Suhaeb , N. A. Rahman , and R. Yusof , “Antiviral Cationic Peptides as a Strategy for Innovation in Global Health Therapeutics for Dengue Virus: High Yield Production of the Biologically Active Recombinant Plectasin Peptide,” OMICS: A Journal of Integrative Biology 17 (2013): 560–567, 10.1089/omi.2013.0056. 24044366 PMC3814901

[106] Y.‐D. Deng , X.‐D. Zhang , X.‐S. Yang , et al., “Subacute Toxicity of Mesoporous Silica Nanoparticles to the Intestinal Tract and the Underlying Mechanism,” Journal of Hazardous Materials 409 (2021): 124502, 10.1016/j.jhazmat.2020.124502. 33229260

[107] X. Li , M. Xue , O. G. Raabe , et al., “Aerosol Droplet Delivery of Mesoporous Silica Nanoparticles: a Strategy for respiratory‐based Therapeutics,” Nanomedicine: Nanotechnology, Biology and Medicine 11 (2015): 1377–1385, 10.1016/j.nano.2015.03.007. 25819886 PMC4494876

[108] G. Zhao , Y. Chen , Y. He , et al., “Succinylated Casein‐coated Peptide‐mesoporous Silica Nanoparticles as an Antibiotic against Intestinal Bacterial Infection,” Biomaterials Science 7 (2019): 2440–2451, 10.1039/C9BM00003H. 30939184

[109] O. A. Saputra , W. A. Lestari , V. Kurniansyah , et al., “Organically Surface Engineered Mesoporous Silica Nanoparticles Control the Release of Quercetin by pH Stimuli,” Scientific Reports 12 (2022): 20661, 10.1038/s41598-022-25095-4. 36450792 PMC9712501

[110] C. Qu , W. Shu , F. Xie , et al., “Dendrimer‐Modified Silica Nanoparticles for Efficient Enrichment of Low‐Concentration Peptides,” Applied Biochemistry and Biotechnology 194 (2022): 3419–3434, 10.1007/s12010-022-03892-x. 35366184

[111] S.‐W. Chen , T. T. A. Hong , C.‐T. Chiang , L.‐K. Chau , and C.‐J. Huang , “Versatile Thiol‐ and Amino‐Functionalized Silatranes for in situ Polymerization and Immobilization of Gold Nanoparticles,” Journal of the Taiwan Institute of Chemical Engineers 132 (2022): 104129, 10.1016/j.jtice.2021.10.029.

[112] P. E. G. Thorén , D. Persson , P. Lincoln , and B. Nordén , “Membrane Destabilizing Properties of Cell‐Penetrating Peptides,” Biophysical Chemistry 114 (2005): 169–179. 15829350 10.1016/j.bpc.2004.11.016

[113] Z. Gounani , M. A. Asadollahi , J. N. Pedersen , et al., “Mesoporous Silica Nanoparticles Carrying Multiple Antibiotics Provide Enhanced Synergistic Effect and Improved Biocompatibility,” Colloids and Surfaces B: Biointerfaces 175 (2019): 498–508, 10.1016/j.colsurfb.2018.12.035. 30572158

[114] C. Shleider Carnero Canales , J. Marquez Cazorla , A. H. Furtado Torres , et al., “Advances in Diagnostics and Drug Discovery against Resistant and Latent Tuberculosis Infection,” Pharmaceutics 15 (2023): 2409, 10.3390/pharmaceutics15102409. 37896169 PMC10610444

[115] J. Palomino and A. Martin , “Drug Resistance Mechanisms in Mycobacterium Tuberculosis,” Antibiotics 3 (2014): 317–340, 10.3390/antibiotics3030317. 27025748 PMC4790366

[116] G. S. Cruz , A. T. dos Santos , E. H. S. de Brito , and G. Rádis‐Baptista , “Cell‐Penetrating Antimicrobial Peptides with Anti‐Infective Activity against Intracellular Pathogens,” Antibiotics 11 (2022): 1772. 36551429 10.3390/antibiotics11121772 PMC9774436

[117] G. del Rio , M. A. Trejo Perez , and C. A. Brizuela , “Antimicrobial Peptides with Cell‐penetrating Activity as Prophylactic and Treatment Drugs,” Bioscience Reports 42 (2022): BSR20221789, 10.1042/BSR20221789. 36052730 PMC9508529

[118] P. M. Perrigue , A. Henschke , B. F. Grześkowiak , et al., “Cellular Uptake and Retention Studies of Silica Nanoparticles Utilizing Senescent Fibroblasts,” Scientific Reports 13 (2023): 475, 10.1038/s41598-022-26979-1. 36627308 PMC9832065

[119] B. Xu , S. Li , R. Shi , and H. Liu , “Multifunctional Mesoporous Silica Nanoparticles for Biomedical Applications,” Signal Transduction and Targeted Therapy 8 (2023): 435, 10.1038/s41392-023-01654-7. 37996406 PMC10667354

[120] M. Asai , Y. Li , J. Spiropoulos , et al., “Galleria Mellonella as an Infection Model for the Virulent Mycobacterium Tuberculosis H37Rv,” Virulence 13 (2022): 1543–1557, 10.1080/21505594.2022.2119657. 36052440 PMC9481108

[121] M. Asai , Y. Li , J. S. Khara , B. D. Robertson , P. R. Langford , and S. M. Newton , “Galleria Mellonella: an Infection Model for Screening Compounds against the Mycobacterium Tuberculosis Complex,” Frontiers in Microbiology 10 (2019): 2630, 10.3389/fmicb.2019.02630. 31824448 PMC6882372

[122] M. Asai , Y. Li , S. M. Newton , B. D. Robertson , and P. R. Langford , “Galleria Mellonella –intracellular Bacteria Pathogen Infection Models: the Ins and Outs,” FEMS Microbiology Reviews 47 (2023): fuad011, 10.1093/femsre/fuad011. 36906279 PMC10045907

[123] G. C. Carvalho , G. D. Marena , G. R. Leonardi , et al., “Lycopene, Mesoporous Silica Nanoparticles and Their Association: a Possible Alternative against Vulvovaginal Candidiasis?,” Molecules 27 (2022): 8558, 10.3390/molecules27238558. 36500650 PMC9738730

[124] A. Cordomí , O. Edholm , and J. J. Perez , “Effect of Different Treatments of Long‐Range Interactions and Sampling Conditions in Molecular Dynamic Simulations of Rhodopsin Embedded in a Dipalmitoyl Phosphatidylcholine Bilayer,” Journal of Computational Chemistry 28 (2007): 1017–1030, 10.1002/jcc.20579. 17269123

[125] K. Kubiak‐Ossowska , G. Burley , S. V. Patwardhan , and P. A. Mulheran , “Spontaneous Membrane‐Translocating Peptide Adsorption at Silica Surfaces: a Molecular Dynamics Study,” The Journal of Physical Chemistry B 117 (2013): 14666–14675, 10.1021/jp409130s. 24176015 PMC3871889

[126] N. Ivanova and A. Ivanova , “Influence of the Dimensionality of the Periodic Boundary Conditions on the Transport of a Drug–peptide Complex across Model Cell Membranes,” Journal of Biomolecular Structure and Dynamics 40 (2022): 5345–5356, 10.1080/07391102.2020.1870157. 33416039

[127] R. Guidelli and L. Becucci , “Functional Activity of Peptide Ion Channels in Tethered Bilayer Lipid Membranes: Review,” Electrochemical Science Advances 2 (2022): 2100180, 10.1002/elsa.202100180.

[128] M. Li , R. Schroder , U. Ozuguzel , et al., “Molecular Insight into Lipid Nanoparticle Assembly from NMR Spectroscopy and Molecular Dynamics Simulation,” Molecular Pharmaceutics 22 (2025): 2193–2212. 40135901 10.1021/acs.molpharmaceut.4c01437

[129] S. A. Mohid , P. Sharma , A. Alghalayini , et al., “A Rationally Designed Synthetic Antimicrobial Peptide Against Pseudomonas‐Associated Corneal Keratitis: Structure‐function Correlation,” Biophysical Chemistry 286 (2022): 106802, 10.1016/j.bpc.2022.106802. 35605494 PMC9167779

[130] Z. Deng , X. Lu , C. Xu , B. Yuan , and K. Yang , “Lipid‐specific Interactions Determine the Organization and Dynamics of Membrane‐active Peptide Melittin,” Soft Matter 16 (2020): 3498–3504, 10.1039/D0SM00046A. 32215386

[131] I. Kabelka and R. Vácha , “Advances in Molecular Understanding of α‐Helical Membrane‐Active Peptides,” Accounts of Chemical Research 54 (2021): 2196–2204, 10.1021/acs.accounts.1c00047. 33844916

[132] M.‐A. Sani and F. Separovic , “How Membrane‐Active Peptides Get into Lipid Membranes,” Accounts of Chemical Research 49 (2016): 1130–1138, 10.1021/acs.accounts.6b00074. 27187572

[133] S. Mori , M. Shionyu , K. Shimamoto , and K. Nomura , “Bacterial Glycolipid Acting on Protein Transport Across Membranes,” Chembiochem 25 (2024): 202300808, 10.1002/cbic.202300808. 38400776

[134] P. Whitley , B. Grau , J. C. Gumbart , L. Martínez‐Gil , and I. Mingarro , “Folding and Insertion of Transmembrane Helices at the ER,” International Journal of Molecular Sciences 22 (2021): 12778, 10.3390/ijms222312778. 34884581 PMC8657811

[135] K. U. Rao , P. Li , C. Welinder , et al., “Mechanisms of a Mycobacterium Tuberculosis Active Peptide,” Pharmaceutics 15 (2023): 540, 10.3390/pharmaceutics15020540. 36839864 PMC9958537

[136] C. Maringolo Ribeiro , C. Augusto Roque‐Borda , M. Carolina Franzini , et al., “Liposome‐siderophore Conjugates Loaded with Moxifloxacin Serve as a Model for Drug Delivery against Mycobacterium Tuberculosis,” International Journal of Pharmaceutics 655 (2024): 124050, 10.1016/j.ijpharm.2024.124050. 38537924

[137] V. W. Rowlett , V. K. P. S. Mallampalli , A. Karlstaedt , et al., “Impact of Membrane Phospholipid Alterations in Escherichia coli on Cellular Function and Bacterial Stress Adaptation,” Journal of Bacteriology 199 (2017): 13, 10.1128/JB.00849-16. PMC5472821 28439040

[138] W. Lin , S. Fan , K. Liao , et al., “Engineering Zinc Oxide Hybrid Selenium Nanoparticles for Synergetic Anti‐Tuberculosis Treatment by Combining Mycobacterium Tuberculosis Killings and Host Cell Immunological Inhibition,” Frontiers in Cellular and Infection Microbiology 12 (2023): 1074533, 10.3389/fcimb.2022.1074533. 36776549 PMC9908760

[139] J. V. Campos , J. T. C. Pontes , C. S. C. Canales , C. A. Roque‐Borda , and F. R. Pavan , “Advancing Nanotechnology: Targeting Biofilm‐Forming Bacteria with Antimicrobial Peptides,” BME Frontiers 6 (2025): 0104, 10.34133/bmef.0104. 40041091 PMC11876546

