# Supplementary material for: Dual‐Peptide Nanoplatform: Mesoporous Silica Nanoparticles Functionalized With a Cell‐Penetrating Peptide and Loaded With Rationally Designed Antimicrobial Peptides for Tuberculosis Therapy
Source: Adv Healthc Mater. 2026 Mar 29;15(21):e04285. doi: 10.1002/adhm.202504285 (PMC13241472; doi:10.1002/adhm.202504285)
Supplement: Supplementary file 1 — Supporting File: adhm71107‐sup‐0001‐SuppMat.docx. [file ADHM-15-0-s001.docx]

**Dual-Peptide Nanoplatform: Mesoporous Silica Nanoparticles Functionalized with a Cell-Penetrating Peptide and Loaded with Rationally Designed Antimicrobial Peptides for Tuberculosis Therapy**

Christian S. Carnero Canales ^1¥^, Cesar Augusto Roque-Borda^1,¥,^, Ana Carolina Cerqueira Negri^1^, Oswaldo Ramirez Delgado^1^, Letícia Oliveira Catarin Nunes^1,2^, Flávia Aparecida Resende^3^, Hernane Barud^3^, Norival Alves Santos-Filho^1,2^, Saulo Santesso Garrido^2^, Karen Cristina Oliveira^4^, Alexandra Ivo de Medeiros^4^, Rafael Miguel Sábio^1*^, Fernando Rogério Pavan^1*^

^1^ Tuberculosis Research Laboratory, School of Pharmaceutical Sciences, São Paulo State University (UNESP), Araraquara, 14800-903, Brazil.

^2^Institute of Chemistry, São Paulo State University (UNESP), Araraquara, 14800-060, Brazil.

^3^ Department of Biological and Health Sciences, University of Araraquara (UNIARA), Araraquara, São Paulo, 14801-320, Brazil

^4^ Department of Biological Sciences, School of Pharmaceutical Sciences, São Paulo State University (UNESP), Araraquara, 14800-903, Brazil.

^¥^ The first authorship is shared

***** **Corresponding authors:**

* E-mail addresses: fernando.pavan@unesp.br (F.R. Pavan) and rafael.m.sabio@unesp.br (R. M. Sábio)


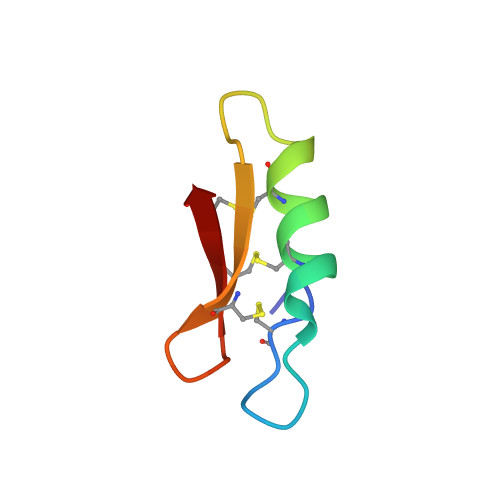


**Figure S1.** 3D structure of Plectasin showing a beta-sheet and an alpha-helix.

**Figure S2.** Ramachandran plots comparing DC05 peptide models generated with AlphaFold3 (A) and I-TASSER (B). (A) The AlphaFold3 model exhibits superior stereochemical quality, with the majority of residues located in the most favored regions (red), and no residues falling into disallowed regions, indicating a structurally reliable conformation. (B) The I-TASSER model shows a lower distribution of residues in favorable zones, with a notable fraction positioned in disallowed regions, suggesting potential conformational strain and lower structural integrity. These results support the selection of the AlphaFold3 model for downstream molecular docking and interaction studies.

**Table S1.** Comparison of Ramachandran Plot Statistics Between AlphaFold3 and I-TASSER Models.

| Category | AlphaFold3 | I-TASSER | Interpretation |
| --- | --- | --- | --- |
| Most Favored Regions [A, B, L] | 81.8% | 36.4% | The first model approaches the expected 90% threshold for a high-quality structure, whereas the second falls significantly below this benchmark. |
| Additionally Allowed Regions [a, b, l, p] | 9.1% | 36.4% | In the second model, a higher proportion of residues lie in "allowed" regions, suggesting potential flexibility but reduced stability. |
| Generously Allowed Regions [~a, ~b, ~l, ~p] | 9.1% | 0.0% | One residue in the first model occupies this region, while none are observed in the second model. |
| Disallowed Regions [XX] | 0.0% | 27.3% | The second model exhibits 27.3% of its residues in unfavorable regions, indicating critical structural flaws. |

**Table S2.** Comparison of G-Factors (Geometric and Conformational Quality).

| Parameter | AlphaFold3 | I-TASSER | Interpretation |
| --- | --- | --- | --- |
| Phi-Psi distribution | -0.72 | -2.42 | The second model shows critically low values, indicating severe deviations in φ and ψ angle distributions. |
| Chi1-Chi2 distribution | -0.05 | -0.74 | The first model exhibits near-normal values, while the second displays a slight deviation. |
| Chi1 only | -1.08 | -1.08 | Both models show atypical side-chain conformations for Chi1 angles. |
| Chi3 & Chi4 | 0.69 | 0.23 | Both values fall within the acceptable range. |
| Omega | 0.55 | -1.48 | The second model exhibits significant deviations in peptide bond torsional angles (Omega). |
| Dihedral angle average | 0.08 | -1.19 | The first model is structurally acceptable, whereas the second lies in an anomalous range. |
| Bond lengths | 0.44 | 0.50 | Both models display normal bond length parameters. |
| Bond angles | 0.58 | -1.04 | The second model shows substantial distortions in bond angles. |
| Overall average | 0.25 | -0.83 | The first model is within the normal range, while the second exhibits major structural deviations. |

**Table S3.** Detailed Information on Binding Affinities and Binding Pockets of DC05 with Different Receptors.

| Protein Complex | binding affinity ΔG (kcal/mol) | Kd (M) | Binding Strength | Residues of binding pockets |
| --- | --- | --- | --- | --- |
| LDT2_DC05 | -10.5 | 3.8 × 10⁻⁸ | Strongest | L203, N204, P286, Y308, V310, L385, I388, N205, T285, Y330, S331 |
| GyrB_DC05 | -10.0 | 9.6 × 10⁻⁸ | Very Strong | H539, L638, D639, D640, A644, L647, F648, L651, F662, I663, D669, V670, R192, V535, Q538, R634, R671, F672, D186 |
| Ag85B_DC05 | -8.1 | 1.8 × 10⁻⁶ | Moderate | Y210, L219, V253, R206, N245, A252, F254, N259 |
| GlfT2_DC05 | -8.2 | 1.6 × 10⁻⁶ | Moderate | I306, W309, T310, A405, I406, K445, W399, H413, K441, A442 |
| PknB_DC05 | -7.8 | 3.2 × 10⁻⁶ | Weak | F39, H125, A194, T237, G41, E233 |
| EmbC_DC05 | -7.8 | 3.0 × 10⁻⁶ | Weak | P102, E105, H309, E319, Y348, G106, R120, R292, Q343 |
| CmA2 | -7.4 | 6.5 × 10⁻⁶ | Weakest | K32, P37, F46, L53, E54, P249, A253, A257, D256, H261 |
| MspA_DC05 | -7.3 | 6.8 × 10⁻⁶ | Weakest | T61, D134, I167, R165, G171, Y177, E127 |

**Table S4.** Interaction Counts per Complex and Classification

| Receptor | Hydrophobic | Hydrogen Bonds | Salt Bridges | Pi-Cation | Pi-Stacking | Total |
| --- | --- | --- | --- | --- | --- | --- |
| Ag85B | 4 | 5 | 0 | 0 | 0 | 9 |
| GyrB | 13 | 9 | 1 | 2 | 0 | 25 |
| LDT2 | 12 | 8 | 0 | 0 | 0 | 20 |
| EmbC | 7 | 8 | 2 | 0 | 0 | 17 |
| CmaA2 | 8 | 2 | 2 | 1 | 0 | 13 |
| PknB | 6 | 3 | 2 | 0 | 1 | 12 |
| MspA | 3 | 4 | 1 | 1 | 0 | 9 |
| GlfT2 | 7 | 6 | 0 | 1 | 0 | 14 |

**Table S5.** Detailed Interaction Information

| Target | Residues (Receptor-Ligand) | Distance (Å) | Category | Interaction Type |
| --- | --- | --- | --- | --- |
| Ag85B | TYR210 (A) - PHE9 (D) | 3.60 | Hydrophobic | Hydrophobic Interaction |
|  | LEU219 (A) - ARG7 (D) | 3.79 | Hydrophobic | Hydrophobic Interaction |
|  | VAL253 (A) - TRP11 (D) | 3.84 | Hydrophobic | Hydrophobic Interaction |
|  | VAL253 (A) - LYS12 (D) | 3.69 | Hydrophobic | Hydrophobic Interaction |
|  | ARG206 (A) - LYS12 (D) | 3.05 | Hydrogen Bond | Conventional Hydrogen Bond |
|  | ASN245 (A) - ARG13 (D) | 1.98 | Hydrogen Bond | Conventional Hydrogen Bond |
|  | ALA252 (A) - LYS12 (D) | 3.43 | Hydrogen Bond | Conventional Hydrogen Bond |
|  | PHE254 (A) - TRP11 (D) | 2.79 | Hydrogen Bond | Conventional Hydrogen Bond |
|  | ASN259 (A) - GLY8 (D) | 3.06 | Hydrogen Bond | Conventional Hydrogen Bond |
| GyrB | HIS539 (A) - LYS6 (D) | 2.48 | Hydrophobic | Hydrophobic Interaction |
|  | LEU638 (A) - TYR14 (D) | 3.21 | Hydrophobic | Hydrophobic Interaction |
|  | ASP639 (A) - TYR14 (D) | 3.85 | Hydrophobic | Hydrophobic Interaction |
|  | ASP640 (A) - TYR14 (D) | 3.46 | Hydrophobic | Hydrophobic Interaction |
|  | ALA644 (A) - TYR14 (D) | 3.54 | Hydrophobic | Hydrophobic Interaction |
|  | LEU647 (A) - LYS12 (D) | 3.92 | Hydrophobic | Hydrophobic Interaction |
|  | PHE648 (A) - TYR3 (D) | 3.55 | Hydrophobic | Hydrophobic Interaction |
|  | LEU651 (A) - TRP11 (D) | 3.06 | Hydrophobic | Hydrophobic Interaction |
|  | PHE662 (A) - TRP11 (D) | 3.26 | Hydrophobic | Hydrophobic Interaction |
|  | PHE662 (A) - TRP11 (D) | 2.82 | Hydrophobic | Hydrophobic Interaction |
|  | ILE663 (A) - PHE9 (D) | 3.92 | Hydrophobic | Hydrophobic Interaction |
|  | ASP669 (A) - VAL10 (D) | 3.70 | Hydrophobic | Hydrophobic Interaction |
|  | VAL670 (A) - PHE9 (D) | 2.87 | Hydrophobic | Hydrophobic Interaction |
|  | ARG192 (A) - LYS12 (D) | 3.18 | Hydrogen Bond | Conventional Hydrogen Bond |
|  | VAL535 (A) - LYS6 (D) | 1.90 | Hydrogen Bond | Conventional Hydrogen Bond |
|  | GLN538 (A) - ARG4 (D) | 2.50 | Hydrogen Bond | Conventional Hydrogen Bond |
|  | ARG634 (A) - TRP2 (D) | 3.08 | Hydrogen Bond | Conventional Hydrogen Bond |
|  | ASP639 (A) - GLY1 (D) | 3.39 | Hydrogen Bond | Conventional Hydrogen Bond |
|  | ARG671 (A) - TRP2 (D) | 2.81 | Hydrogen Bond | Conventional Hydrogen Bond |
|  | ARG671 (A) - GLY8 (D) | 2.29 | Hydrogen Bond | Conventional Hydrogen Bond |
|  | PHE672 (A) - GLY8 (D) | 2.37 | Hydrogen Bond | Conventional Hydrogen Bond |
|  | PHE672 (A) - GLY8 (D) | 3.48 | Hydrogen Bond | Conventional Hydrogen Bond |
|  | ASP186 (A) - LYS12 (D) | 5.34 | Salt Bridge | Salt Bridge |
|  | HIS539 (A) - LYS6 (D) | 3.42 | Pi-Cation | Pi-Cation Interaction |
|  | ARG671 (A) - TRP2 (D) | 4.26 | Pi-Cation | Pi-Cation Interaction |
| LDT2 | LEU203 (A) - LYS6 (D) | 3.53 | Hydrophobic | Hydrophobic Interaction |
|  | ASN204 (A) - LYS6 (D) | 3.55 | Hydrophobic | Hydrophobic Interaction |
|  | ASN204 (A) - ARG7 (D) | 3.85 | Hydrophobic | Hydrophobic Interaction |
|  | PRO286 (A) - TYR14 (D) | 1.63 | Hydrophobic | Hydrophobic Interaction |
|  | PRO286 (A) - TYR14 (D) | 2.75 | Hydrophobic | Hydrophobic Interaction |
|  | TYR308 (A) - TRP2 (D) | 3.17 | Hydrophobic | Hydrophobic Interaction |
|  | TYR308 (A) - TRP2 (D) | 2.58 | Hydrophobic | Hydrophobic Interaction |
|  | TYR308 (A) - VAL10 (D) | 3.54 | Hydrophobic | Hydrophobic Interaction |
|  | TYR308 (A) - ARG4 (D) | 3.29 | Hydrophobic | Hydrophobic Interaction |
|  | VAL310 (A) - TRP2 (D) | 2.51 | Hydrophobic | Hydrophobic Interaction |
|  | LEU385 (A) - TYR3 (D) | 3.64 | Hydrophobic | Hydrophobic Interaction |
|  | ILE388 (A) - LYS6 (D) | 3.71 | Hydrophobic | Hydrophobic Interaction |
|  | ASN204 (A) - LYS6 (D) | 3.36 | Hydrogen Bond | Conventional Hydrogen Bond |
|  | ASN205 (A) - ARG7 (D) | 3.08 | Hydrogen Bond | Conventional Hydrogen Bond |
|  | ASN205 (A) - ARG7 (D) | 2.92 | Hydrogen Bond | Conventional Hydrogen Bond |
|  | THR285 (A) - GLY1 (D) | 3.57 | Hydrogen Bond | Conventional Hydrogen Bond |
|  | TYR308 (A) - ARG4 (D) | 3.15 | Hydrogen Bond | Conventional Hydrogen Bond |
|  | TYR330 (A) - ARG4 (D) | 2.97 | Hydrogen Bond | Conventional Hydrogen Bond |
|  | SER331 (A) - ARG4 (D) | 2.69 | Hydrogen Bond | Conventional Hydrogen Bond |
|  | ILE388 (A) - LYS6 (D) | 2.25 | Hydrogen Bond | Conventional Hydrogen Bond |
| EmbC | PRO102 (A) - TRP2 (D) | 3.87 | Hydrophobic | Hydrophobic Interaction |
|  | GLU105 (A) - ARG4 (D) | 3.64 | Hydrophobic | Hydrophobic Interaction |
|  | HIS309 (A) - LYS12 (D) | 3.91 | Hydrophobic | Hydrophobic Interaction |
|  | GLU319 (A) - LYS12 (D) | 3.73 | Hydrophobic | Hydrophobic Interaction |
|  | GLU319 (A) - LYS12 (D) | 3.29 | Hydrophobic | Hydrophobic Interaction |
|  | TYR348 (A) - PHE9 (D) | 3.78 | Hydrophobic | Hydrophobic Interaction |
|  | TYR348 (A) - PHE9 (D) | 3.28 | Hydrophobic | Hydrophobic Interaction |
|  | GLY106 (A) - ARG4 (D) | 1.86 | Hydrogen Bond | Conventional Hydrogen Bond |
|  | GLY106 (A) - ARG4 (D) | 1.76 | Hydrogen Bond | Conventional Hydrogen Bond |
|  | ARG120 (A) - ARG4 (D) | 3.00 | Hydrogen Bond | Conventional Hydrogen Bond |
|  | ARG292 (A) - ARG13 (D) | 3.07 | Hydrogen Bond | Conventional Hydrogen Bond |
|  | HIS309 (A) - LYS12 (D) | 2.30 | Hydrogen Bond | Conventional Hydrogen Bond |
|  | GLU319 (A) - ARG13 (D) | 1.76 | Hydrogen Bond | Conventional Hydrogen Bond |
|  | GLN343 (A) - ARG13 (D) | 1.32 | Hydrogen Bond | Conventional Hydrogen Bond |
|  | GLN343 (A) - ARG13 (D) | 2.82 | Hydrogen Bond | Conventional Hydrogen Bond |
|  | GLU105 (A) - ARG4 (D) | 3.83 | Salt Bridge | Salt Bridge |
|  | GLU319 (A) - LYS12 (D) | 3.47 | Salt Bridge | Salt Bridge |
| CmaA2 | LYS32 (A) - LYS12 (D) | 3.93 | Hydrophobic | Hydrophobic Interaction |
|  | PRO37 (A) - TRP2 (D) | 3.19 | Hydrophobic | Hydrophobic Interaction |
|  | PHE46 (A) - PHE9 (D) | 2.66 | Hydrophobic | Hydrophobic Interaction |
|  | LEU53 (A) - PHE9 (D) | 3.61 | Hydrophobic | Hydrophobic Interaction |
|  | GLU54 (A) - VAL10 (D) | 3.58 | Hydrophobic | Hydrophobic Interaction |
|  | PRO249 (A) - PHE9 (D) | 3.53 | Hydrophobic | Hydrophobic Interaction |
|  | ALA253 (A) - PHE9 (D) | 3.46 | Hydrophobic | Hydrophobic Interaction |
|  | ALA257 (A) - TRP11 (D) | 3.01 | Hydrophobic | Hydrophobic Interaction |
|  | LEU53 (A) - GLY8 (D) | 3.34 | Hydrogen Bond | Conventional Hydrogen Bond |
|  | GLU54 (A) - ARG13 (D) | 4.38 | Salt Bridge | Salt Bridge Interaction |
|  | ASP256 (A) - LYS6 (D) | 4.31 | Salt Bridge | Salt Bridge Interaction |
|  | HIS261 (A) - TYR3 (D) | 4.77 | Pi-Cation | Pi-Cation Interaction |
| PknB | PHE39 (A) - TYR14 (D) | 3.64 | Hydrophobic | Hydrophobic Interaction |
|  | PHE39 (A) - TYR14 (D) | 2.98 | Hydrophobic | Hydrophobic Interaction |
|  | HIS125 (A) - TYR3 (D) | 3.91 | Hydrophobic | Hydrophobic Interaction |
|  | ALA194 (A) - TRP2 (D) | 3.80 | Hydrophobic | Hydrophobic Interaction |
|  | ALA194 (A) - ARG13 (D) | 3.47 | Hydrophobic | Hydrophobic Interaction |
|  | THR237 (A) - PHE9 (D) | 3.17 | Hydrophobic | Hydrophobic Interaction |
|  | GLY41 (A) - LYS12 (D) | 3.14 | Hydrogen Bond | Conventional Hydrogen Bond |
|  | HIS125 (A) - ARG4 (D) | 2.60 | Hydrogen Bond | Conventional Hydrogen Bond |
|  | GLY232 (A) - LYS6 (D) | 2.39 | Hydrogen Bond | Conventional Hydrogen Bond |
|  | GLU233 (A) - LYS6 (D) | 5.48 | Salt Bridge | Salt Bridge |
|  | GLU233 (A) - ARG7 (D) | 2.05 | Salt Bridge | Salt Bridge |
|  | HIS125 (A) - TYR3 (D) | 5.02 | Pi-Stacking | Pi-Stacking Interaction |
| MspA | THR61 (B) - PHE9 (D) | 3.04 | Hydrophobic | Hydrophobic Interaction |
|  | ASP134 (B) - PHE9 (D) | 3.67 | Hydrophobic | Hydrophobic Interaction |
|  | ILE167 (B) - VAL10 (D) | 3.04 | Hydrophobic | Hydrophobic Interaction |
|  | ARG165 (B) - ARG13 (D) | 2.69 | Hydrogen Bond | Conventional Hydrogen Bond |
|  | GLY171 (B) - ARG4 (D) | 2.54 | Hydrogen Bond | Conventional Hydrogen Bond |
|  | TYR177 (B) - ARG13 (D) | 1.80 | Hydrogen Bond | Conventional Hydrogen Bond |
|  | TYR177 (B) - ARG13 (D) | 1.06 | Hydrogen Bond | Conventional Hydrogen Bond |
| GlfT2 | ILE306 (A) - TRP11 (D) | 2.79 | Hydrophobic | Hydrophobic Interaction |
|  | TRP309 (A) - LYS12 (D) | 3.45 | Hydrophobic | Hydrophobic Interaction |
|  | THR310 (A) - TRP11 (D) | 3.94 | Hydrophobic | Hydrophobic Interaction |
|  | ALA405 (A) - LYS12 (D) | 3.03 | Hydrophobic | Hydrophobic Interaction |
|  | ALA405 (A) - TYR14 (D) | 3.85 | Hydrophobic | Hydrophobic Interaction |
|  | ILE406 (A) - TYR14 (D) | 2.83 | Hydrophobic | Hydrophobic Interaction |
|  | LYS445 (A) - TYR3 (D) | 3.35 | Hydrophobic | Hydrophobic Interaction |
|  | TRP399 (A) - ARG13 (D) | 2.61 | Hydrogen Bond | Conventional Hydrogen Bond |
|  | HIS413 (A) - LYS12 (D) | 3.04 | Hydrogen Bond | Conventional Hydrogen Bond |
|  | LYS441 (A) - ARG4 (D) | 3.40 | Hydrogen Bond | Conventional Hydrogen Bond |
|  | ALA442 (A) - TYR3 (D) | 3.15 | Hydrogen Bond | Conventional Hydrogen Bond |
|  | LYS445 (A) - GLY1 (D) | 2.75 | Hydrogen Bond | Conventional Hydrogen Bond |
|  | LYS445 (A) - GLY1 (D) | 2.77 | Hydrogen Bond | Conventional Hydrogen Bond |
|  | LYS445 (A) - TYR3 (D) | 3.74 | Pi-Cation | Pi-Cation Interaction |

**Table S6.** Key Residues of the DC05 Peptide and Their Participation in Critical Interactions.

| Peptide Residue | Key Interactions | Complexes Where Involved |
| --- | --- | --- |
| Trp2 (W2) | - Hydrophobic | GyrB, LDT2, PknB, CmaA2 |
|  | - Hydrogen Bonds |  |
| Tyr3 (Y3) | - Hydrophobic | LDT2, PknB, GlfT2, CmaA2 |
|  | - Pi-Cation |  |
| Arg4 (R4) | - Hydrogen Bonds | GyrB, LDT2, EmbC |
|  | - Salt Bridges |  |
| Lys6 (K6) | - Hydrophobic | GyrB, LDT2, CmaA2 |
|  | - Hydrogen Bonds |  |
| Arg7 (R7) | - Hydrophobic | Ag85B, LDT2 |
|  | - Hydrogen Bonds |  |
| Phe9 (F9) | - Hydrophobic | Ag85B, EmbC, CmaA2, PknB |
|  | - Pi-Stacking |  |
| Trp11 (W11) | - Hydrophobic | Ag85B, GyrB, CmaA2 |
|  | - Hydrogen Bonds |  |
| Lys12 (K12) | - Hydrophobic | Ag85B, GyrB, EmbC, GlfT2, CmaA2 |
|  | - Hydrogen Bonds |  |
| Arg13 (R13) | - Hydrogen Bonds | Ag85B, EmbC, MspA, GlfT2, CmaA2 |
|  | - Salt Bridges |  |
| Tyr14 (Y14) | - Hydrophobic | LDT2, GlfT2, GyrB |
|  | - Pi-Cation |  |


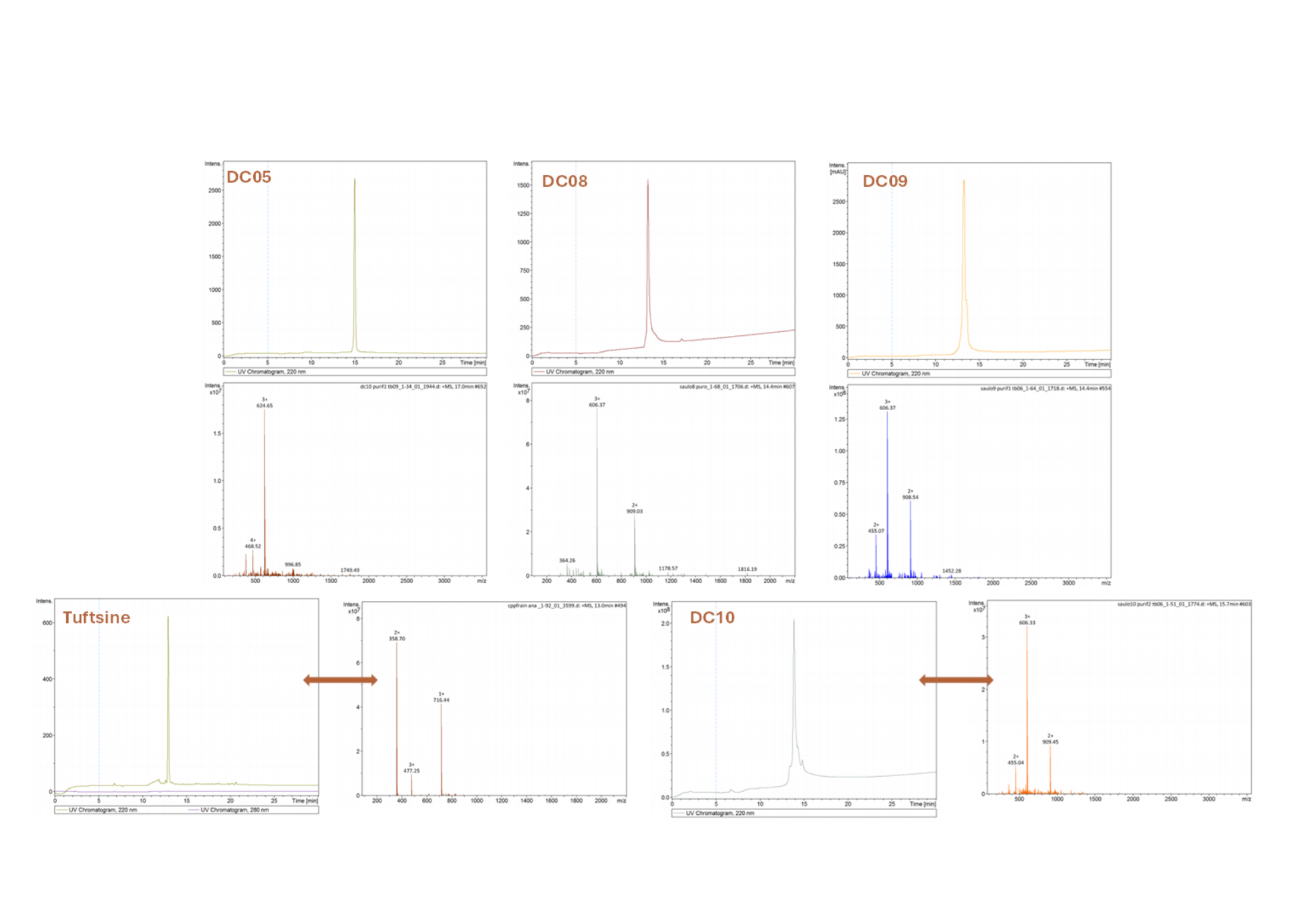


**Figure S3.** LC/MS Profile of the Synthesized Peptides. The theoretical mass corresponds to the mass obtained by the mass spectrum, suggesting that the synthesis process was performed correctly. The spectrum profile indicates an acceptable purity.

**Table S7.** Predicted pharmacokinetic properties of Plectasin analogs based on *in silico* analysis

| Pharmacokinetic properties | | | DC05 | DC08 | DC09 | DC10 |  |
| --- | --- | --- | --- | --- | --- | --- | --- |
| Properties | **Model name** | **Units** | **Expected value** | | | | |
| Absorption | Water solubility | Numeric (log mol/L) | -2.892 | -2.892 | -2.892 | -2.892 |  |
|  | Caco2 permeability | Numerical (log Papp at 10-6 cm/s) | -1.318 | -1.337 | -1.335 | -1.169 |  |
|  | Intestinal absorption (human) | Numerical (% absorbed) | 0 | 0 | 0 | 0 |  |
|  | Skin permeability | Numerical (log Kp) | -2.735 | -2.735 | -2.735 | -2.735 |  |
|  | P-glycoprotein substrate | Categorical (Yes/No) | Yes | Yes | Yes | Yes |  |
|  | P-glycoprotein I inhibitor |  | No | No | No | No |  |
|  | P-glycoprotein II inhibitor |  | No | No | No | No |  |
| Distribution | VDss (human) | Numerical (log L/kg) | 0.01 | 0.01 | 0.01 | 0.01 |  |
|  | Unbound fraction (human) | Numerical (Fu) | 0.381 | 0.381 | 0.381 | 0.381 |  |
|  | BBB permeability | Numerical (log BB) | -4.974 | -5.191 | -5.193 | -5.06 |  |
|  | CNS permeability | Numerical (log PS) | -10.092 | -10.529 | -10.523 | -10.205 |  |
| Metabolism | CYP2D6 substrate | Categorical (Yes/No) | No | No | No | No |  |
|  | CYP3A4 substrate |  | Yes | Yes | Yes | Yes |  |
|  | CYP1A2 inhibitor |  | No | No | No | No |  |
|  | CYP2C19 inhibitor |  | No | No | No | No |  |
|  | CYP2C9 inhibitor |  | No | No | No | No |  |
|  | CYP2D6 inhibitor |  | No | No | No | No |  |
|  | CYP3A4 inhibitor |  | No | No | No | No |  |
| Excretion | Total clearance | Categorical (Yes/No) | -1.319 | -1.471 | -1.445 | -0.961 |  |
|  | OCT2 renal substrate | Categorical (Yes/No) | No | No | No | No |  |
| Toxicity | Maximum tolerated dose (human) | Numerical (log ml/min/kg) | 0.438 | 0.438 | 0.438 | 0.438 |  |
|  | AMES toxicity | Categorical (Yes/No) | No | No | No | No |  |
|  | Acute oral toxicity in rats (LD50) | Numerical (mol/kg) | 2.482 | 2.482 | 2.482 | 2.482 |  |
|  | Chronic oral toxicity in rats (LOAEL) | Numerical  (log mg/kg bw/dia) | 20.164 | 17.693 | 17.446 | 16.943 |  |
|  | T. Pyriformis Toxicity | Numeric (log ug/L) | 0.285 | 0.285 | 0.285 | 0.285 |  |
|  | Minnow Toxicity | Numeric (log mM) | 31.35 | 32.555 | 34.938 | 33.526 |  |
|  | Hepatotoxicity | Categorical (Yes/No) | No | No | No | Yes |  |
|  | skin sensitization |  | No | No | No | No |  |
|  | hERG I inhibitor |  | No | No | No | No |  |
|  | hERG II inhibitor |  | No | No | No | Yes |  |

^* Permeability of the blood-brain barrier (BBB), central nervous system (CNS), Volume of distribution at steady state (VDss).^

**Table S8.** *In silico* predicted half-life of peptide sequences across different biological models

| AMP | Model | Peptide half-life |
| --- | --- | --- |
| DC05 | Mammalian reticulocytes | 30 H |
|  | Yeast | >20 H |
|  | *E.coli* | >10 H |
| DC08 | Mammalian reticulocytes | 2.8 H |
|  | Yeast | 10 min |
|  | *E.coli* | 2min |
| DC09 | Mammalian reticulocytes | 30 H |
|  | Yeast | >20 H |
|  | *E.coli* | >10 H |
| DC10 | Mammalian reticulocytes | 1 H |
|  | Yeast | 2 min |
|  | *E.coli* | 2 min |


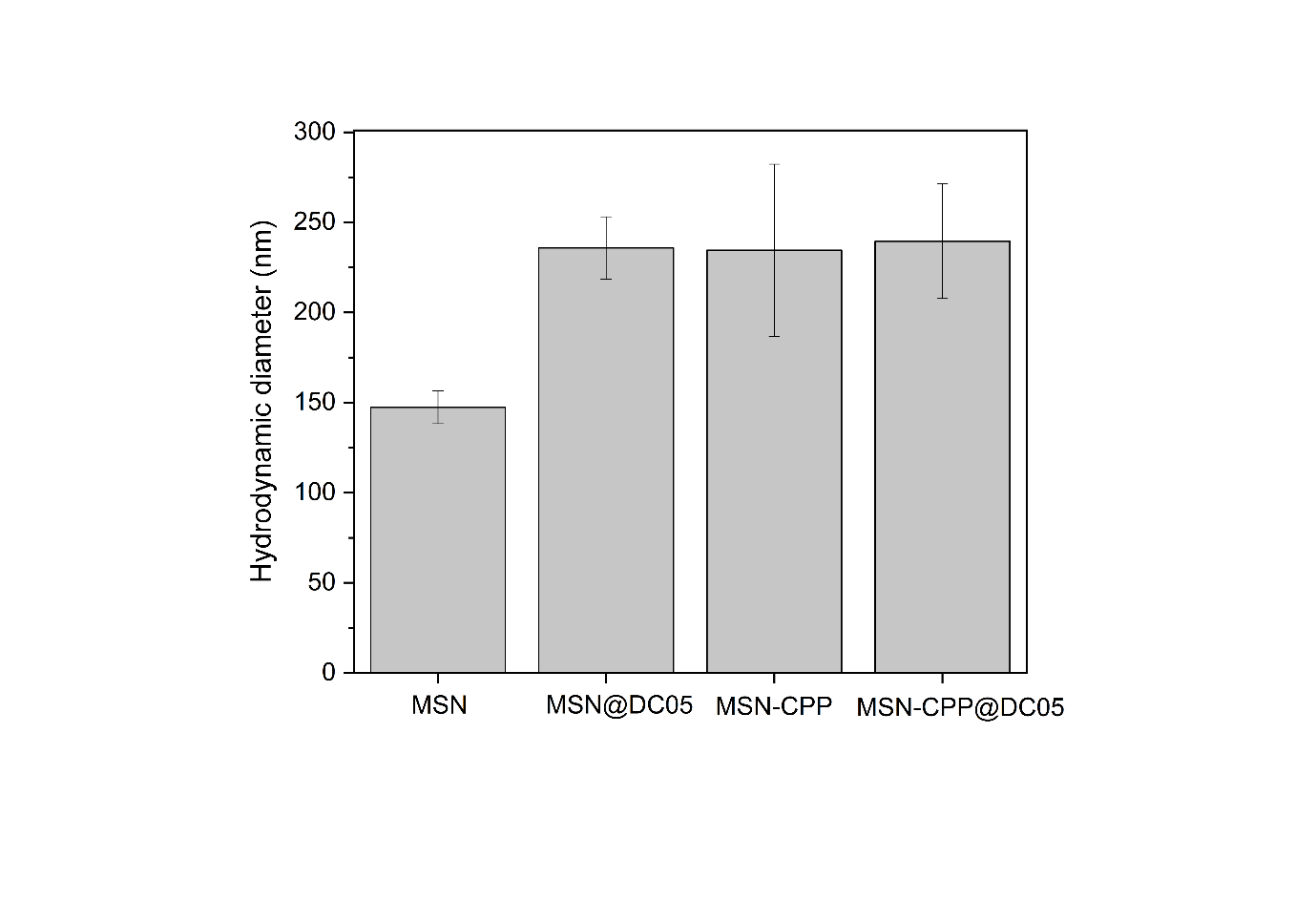


**Figure S4.** Hydrodynamic diameter in HEPES 10mM at 0.5 g mL⁻¹


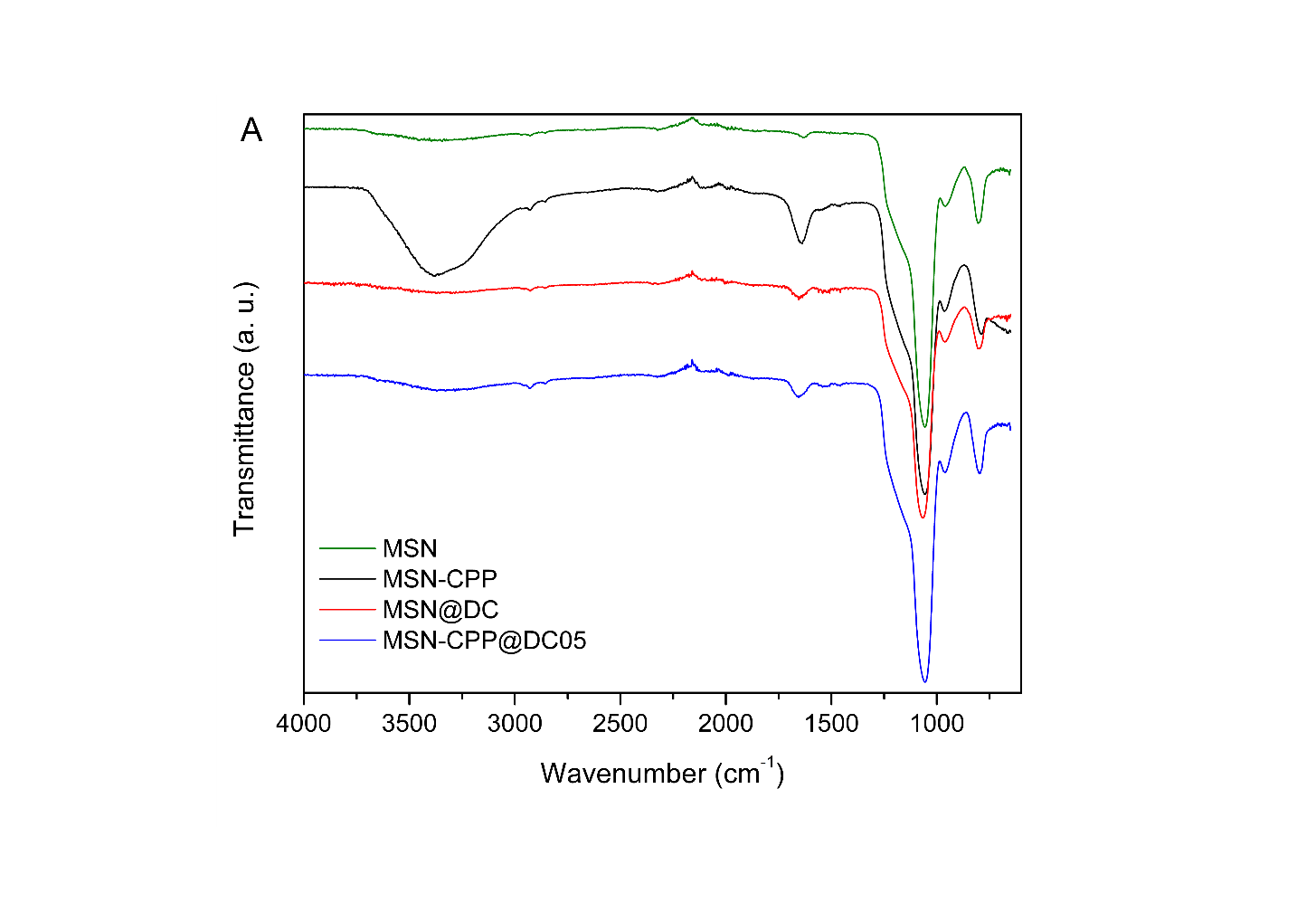


**Figure S5.** (**A**) FTIR spectra of MSNs, MSN-CPP, MSN@DC05, and MSN-CPP@DC05 recorded in KBr pellets.


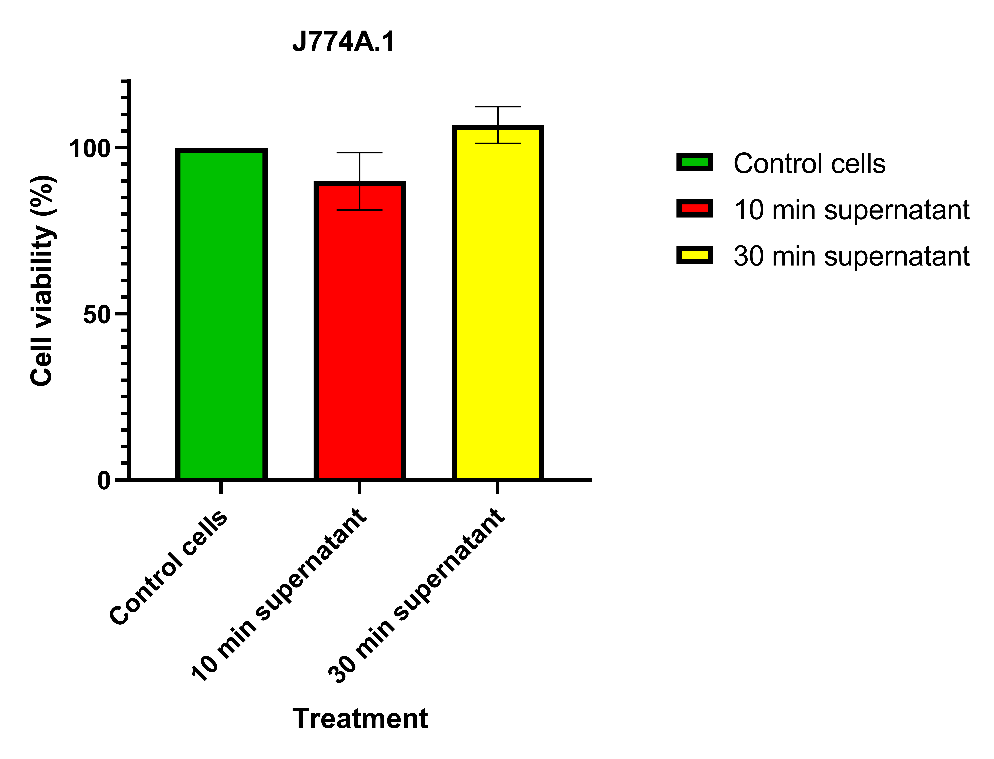


**Figure S6.** Premature peptide release assay. Cell viability of J774A.1 macrophages after exposure to particle-free supernatants obtained after incubation of MSN-CPP@DC05 nanoparticles (1 mg mL⁻¹) in RPMI-1640 for 10 and 30 min under cell-free conditions. After incubation, suspensions were centrifuged at 15,000 × g for 15 min and the resulting supernatants were collected. For the cell assay, 100 µL of supernatant were added to wells containing 100 µL of RPMI-1640 medium, and macrophages were treated following the cytotoxicity protocol described in Section 2.3.3 of the main text. Cell viability was determined using the resazurin assay and expressed as percentage relative to untreated cells. Data represent mean ± SD (n = 3). No significant differences were observed between groups (one-way ANOVA with Dunnett’s test, p > 0.05).
